# Association of Prehospital Plasma With Survival in Patients With Traumatic Brain Injury: A Secondary Analysis of the PAMPer Cluster Randomized Clinical Trial

**DOI:** 10.1001/jamanetworkopen.2020.16869

**Published:** 2020-10-15

**Authors:** Danielle S. Gruen, Francis X. Guyette, Joshua B. Brown, David O. Okonkwo, Ava M. Puccio, Insiyah K. Campwala, Matthew T. Tessmer, Brian J. Daley, Richard S. Miller, Brian G. Harbrecht, Jeffrey A. Claridge, Herb A. Phelan, Matthew D. Neal, Brian S. Zuckerbraun, Mark H. Yazer, Timothy R. Billiar, Jason L. Sperry

**Affiliations:** 1 Department of Surgery and Critical Care Medicine, University of Pittsburgh, Pittsburgh, Pennsylvania; 2 Pittsburgh Trauma Research Center, Pittsburgh, Pennsylvania; 3 Department of Emergency Medicine, University of Pittsburgh, Pittsburgh, Pennsylvania; 4 Department of Neurological Surgery, University of Pittsburgh, Pittsburgh, Pennsylvania; 5 Department of Surgery, University of Tennessee Health Science Center, Knoxville; 6 Department of Surgery, Vanderbilt University Medical Center, Nashville, Tennessee; 7 Department of Surgery, University of Louisville, Louisville, Kentucky; 8 MetroHealth Medical Center, Case Western Reserve University, Cleveland, Ohio; 9 Department of Surgery, University of Texas Southwestern, Dallas; 10 Department of Pathology, University of Pittsburgh, Pittsburgh, Pennsylvania

## Abstract

**Importance:**

Prehospital plasma administration improves survival in injured patients at risk for hemorrhagic shock and transported by air ambulance. Traumatic brain injury (TBI) is a leading cause of death following trauma, but few early interventions improve outcomes.

**Objective:**

To assess the association between prehospital plasma and survival in patients with TBI.

**Design, Setting, and Participants:**

The Prehospital Air Medical Plasma (PAMPer) trial was a pragmatic, multicenter, phase 3, cluster randomized clinical trial involving injured patients who were at risk for hemorrhagic shock during air medical transport to a trauma center. The trial was conducted at 6 US sites with 9 level-I trauma centers (comprising 27 helicopter emergency services bases). The original trial analyzed 501 patients, including 230 patients who were randomized to receive plasma and 271 randomized to standard care resuscitation. This secondary analysis of a predefined subgroup included patients with TBI. Data analysis was performed from October 2019 to February 2020.

**Interventions:**

Patients were randomized to receive standard care fluid resuscitation or 2 units of thawed plasma.

**Main Outcomes and Measures:**

The primary outcome was mortality at 30 days. Patients with TBI were prespecified as a subgroup for secondary analysis and for measurement of markers of brain injury. The 30-day survival benefit of prehospital plasma in subgroups with and without TBI as diagnosed by computed tomography was characterized using Kaplan-Meier survival analysis and Cox proportional hazard regression.

**Results:**

In total, 166 patients had TBI (median [interquartile range] age, 43.00 [25.00-59.75] years; 125 men [75.3%]). When compared with the 92 patients who received standard care, the 74 patients with TBI who received prehospital plasma had improved 30-day survival even after adjustment for multiple confounders and assessment of the degree of brain injury with clinical variables and biomarkers (hazard ratio [HR], 0.55; 95% CI, 0.33-0.94; *P* = .03). Receipt of prehospital plasma was associated with improved survival among patients with TBI with a prehospital Glasgow Coma Scale score of less than 8 (HR, 0.56; 95% CI, 0.35-0.91) and those with polytrauma (HR, 0.50; 95% CI, 0.28-0.89). Patients with TBI transported from the scene of injury had improved survival following prehospital plasma administration (HR, 0.45; 95% CI, 0.26-0.80; *P* = .005), whereas patients who were transferred from an outside hospital showed no difference in survival for the plasma intervention (HR, 1.00; 95% CI, 0.33-3.00; *P* = .99).

**Conclusions and Relevance:**

These findings are exploratory, but they suggest that receipt of prehospital plasma is associated with improved survival in patients with computed tomography–positive TBI. The prehospital setting may be a critical period to intervene in the care of patients with TBI. Future studies are needed to confirm the clinical benefits of early plasma resuscitation following TBI and concomitant polytrauma.

**Trial Registration:**

ClinicalTrials.gov Identifier: NCT01818427

## Key Points

**Question:**

Is prehospital plasma administration associated with improved survival among severely injured patients with traumatic brain injury (TBI)?

**Findings:**

In this secondary analysis of a predefined subgroup of 166 severely injured patients with trauma from a cluster randomized clinical trial, prehospital plasma administration was associated with lower mortality in patients with TBI.

**Meaning:**

Future studies are needed to confirm these clinical benefits, but prehospital plasma administered soon after injury may improve survival for patients with TBI.

## Teaser

This secondary analysis of a cluster randomized clinical trial assesses the association between prehospital plasma administration and survival in patients with traumatic brain injury.

## Introduction

Traumatic injury is a leading cause of preventable death,^1,2^ which results primarily from hemorrhage and traumatic brain injury (TBI).^3^ Worldwide, it is estimated that there are 50 million cases of TBI each year, generating a global annual economic burden of approximately US $400 billion.^4^ Despite efforts to address this global health problem,^5,6^ there are limited therapies to lessen the morbidity and mortality associated with TBI.^4^ Increasingly, prehospital interventions following traumatic injury result in improved outcomes and survival,^7,8,9^ and this phase of care may be especially relevant to the treatment of TBI.^4,10,11^

The Prehospital Air Medical Plasma (PAMPer) trial^8^ demonstrated that prehospital plasma resuscitation improved 30-day survival by 10% in severely injured trauma patients at risk for hemorrhagic shock and transported by air ambulance. Several underlying mechanisms to explain this survival benefit are hypothesized, including volume expansion to restore perfusion, an alteration in the inflammatory response,^12,13^ a reduction in endothelial injury,^14,15^ and the prevention or mitigation of coagulopathy.^10^ Retrospective studies and animal models^16,17^ suggest that plasma administered soon after injury may improve neurological function after TBI. Prehospital interventions that mitigate secondary injury from hypotension, hypoxia, and hypocarbia have also been shown to improve TBI outcomes.^11,18,19,20^ Preliminary, unadjusted subgroup analysis in the PAMPer trial suggested a survival benefit in patients with severe head injury, as defined by Abbreviated Injury Severity (AIS) scores for the head.^8^ However, to our knowledge, the association of early plasma administration with survival has not been fully characterized in the subgroup of patients with TBI as confirmed by computed tomography (CT) while adjusting for potential confounding factors.

Identifying patients who benefit from early interventions enables the targeted allocation of resources to the most appropriate patients.^21,22,23^ Whether the beneficial effect of prehospital plasma differs across the spectrum of TBI is unknown. In this analysis, we sought to characterize the survival benefit associated with prehospital plasma among patients with TBI using data derived from a recently completed prehospital plasma clinical trial.

## Methods

We performed a post hoc secondary analysis of a prespecified subgroup from the PAMPer trial. The PAMPer trial was a pragmatic, multicenter, phase 3, cluster randomized clinical superiority trial designed to test the effect of administering plasma to severely injured trauma patients on air ambulances before arrival to definitive trauma care. We randomized patients to receive either standard care fluid resuscitation (crystalloid or crystalloid and packed red blood cells) or 2 units of thawed plasma followed by standard care fluid resuscitation. This study was approved under an Emergency Exception From Informed Consent protocol from the Human Research Protection Office of the US Army Medical Research and Material Command and by the appropriate institutional review boards as previously described.^8^ The full trial protocol is available in Supplement 1. Additional information regarding study inclusion and exclusion criteria and model analysis methods can be found in the eAppendix in Supplement 2. This study follows the Consolidated Standards of Reporting Trials (CONSORT) reporting guideline.

TBI was defined in the study protocol as a prespecified subgroup for a post hoc, secondary analysis. For the primary analysis, TBI was defined by AIS score for the head greater than 2.^8^ CT imaging is more accurate than head AIS score for determining the presence of TBI. In this study, we assessed imaging results and defined TBI as brain injury documented by CT scan. TBI was defined as any finding consistent with TBI as defined by a radiologist at initial head CT. We analyzed glial fibrillary acidic protein (GFAP) and ubiquitin C-terminal hydrolase (UCH-L1) to assess biological markers of TBI across trial groups as described elsewhere.^24,25^

### Statistical Analysis

We first evaluated the association of prehospital plasma with 30-day mortality across groups with and without TBI (binned as a dichotomous variable) using a generalized estimating equations model in the geepack software package version 1.2-1 (R Project for Statistical Computing).^26^ The plasma and TBI interaction was assessed for statistical significance, accounting for trial cluster effects and multiple confounders. We then performed Kaplan-Meier survival analysis comparing prehospital plasma vs standard care resuscitation across groups according to the presence of TBI. Log-rank *P* values were calculated and survival curves were built using the Survminer software package version 0.4.3 (R Project for Statistical Computing).^27^

To verify these unadjusted findings, we performed an analysis of survival with the use of a Cox proportional hazard with shared frailty model to evaluate the treatment with adjustment for possible confounding factors and site clustering on survival.^28^ We generated the model for the primary outcome and assessed prehospital differences across the groups when randomization for prehospital plasma occurred. We assessed whether our fitted Cox regression model adequately described the data using the Survival software package version 2.41-3 (R Project for Statistical Computing).^29^ We evaluated variance inflation factors with the rms software package version 5.1-3.1 (R Project for Statistical Computing)^30^ to ensure that the variance of our regression coefficients was not due to multicollinearity. This model met the proportional hazards assumption on the basis of Schoenfeld residuals.

We explored the association between prehospital plasma and both TBI severity and concomitant polytrauma by comparison of hazard ratios (HRs) from a fitted Cox proportional hazard model. We analyzed survival for patients with TBI transported from the scene and those transferred from an outside hospital as proxies for early vs late prehospital plasma fluid administration.

We analyzed data using R statistical software version 3.4.1 (R Project for Statistical Computing).^31^ The analysis code is publicly available on GitHub.^32^ Categorical variables are presented as frequencies and percentages and were tested using the Pearson χ^2^ test. Continuous variables are expressed as medians and interquartile ranges (IQRs) and were tested using Mann-Whitney *U* or Kruskal-Wallis test, as appropriate; all statistical tests were 2-sided. Statistical significance was determined at the *P* *<* .05 level. Data analysis was performed from October 2019 to February 2020.

## Results

**Figure 1.**
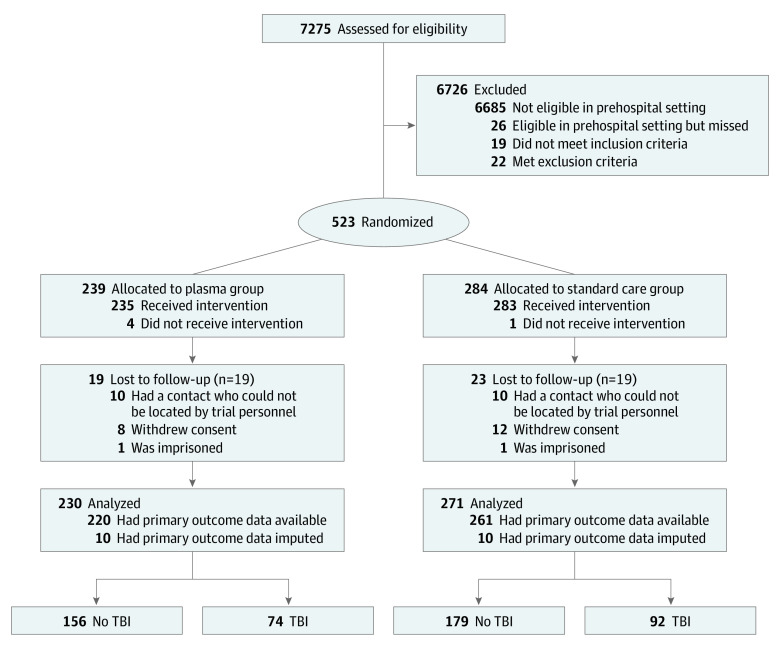
Patient Selection Flowchart TBI indicates traumatic brain injury.

Of the 501 PAMPer patients, 166 patients (median [IQR] age, 43.00 [25.00-59.75] years) sustained a TBI as indicated by the presence of brain injury on CT imaging. Patients with TBI were mostly male (125 men [75.3%]), all of them had blunt trauma injuries (100%), and they had a median (IQR) Injury Severity Score of 29 (22-38) (Figure 1 and Table 1).

**Table 1.** Comparison of Patient and Injury Characteristics and Outcomes Across Patients With and Without TBI

Variable	Patients, No. (%)		*P* value^a^
	No TBI (n = 335)	TBI (n = 166)	
Demographic characteristics			
Age, median (IQR), y	46.00 (31.00-59.50)	43.00 (25.00-59.75)	.46
Male	239 (71.3)	125 (75.3)	.41
Race			
White	287 (85.7)	148 (89.2)	.10
Black	35 (10.4)	8 (4.8)	
Asian	1 (0.3)	0	
Other	3 (0.9)	5 (3.0)	
Unknown	9 (2.7)	5 (3.0)	
Medications receiving			
Vitamin K antagonist	12 (4.4)	2 (1.8)	.33
Antiplatelets	27 (9.7)	11 (9.6)	>.99
Injury characteristics			
Injury Severity Score, median (IQR)	17 (9-25)	29 (22-38)	<.001
Abbreviated Injury Severity score, median (IQR)			
Head	0 (0-2)	4 (3-5)	<.001
Chest	2 (0-3)	3 (2-3)	.01
Abdomen	2 (0-3)	2 (0-2)	.09
Extremity	2 (0-3)	2 (0-3)	.47
Glasgow Coma Scale score			
Prehospital			
<8	95 (29.3)	123 (75.5)	<.001
8-12	34 (10.5)	13 (8.0)	
13-15	195 (60.2)	27 (16.6)	
Emergency department			
<8	144 (43.8)	132 (81.0)	<.001
8-12	5 (1.5)	8 (4.9)	
13-15	180 (54.7)	23 (14.1)	
Prehospital systolic blood pressure <70 mm Hg	164 (49.1)	82 (49.4)	>.99
Spinal cord injury	21 (6.3)	17 (10.3)	.16
Blunt injury	247 (73.7)	166 (100.0)	<.001
Blunt mechanism			
Fall	21 (8.5)	14 (8.4)	.32
Motor vehicle crash			
Occupant	137 (55.5)	89 (53.6)	
Motorcyclist	40 (16.2)	35 (21.1)	
Pedestrian or cyclist	13 (5.2)	15 (9.0)	
Unknown	2 (0.8)	1 (0.6)	
Struck by or against	16 (6.5)	3 (1.8)	
Other	18 (7.3)	9 (5.4)	
Prehospital			
Intubation	131 (39.1)	125 (75.3)	<.001
Cardiopulmonary resuscitation	19 (5.7)	12 (7.2)	.63
Plasma intervention	156 (46.6)	74 (44.6)	.75
Crystalloid fluid, median (IQR), mL	600.00 (0.00-1440.00)	812.50 (0.00-1475.00)	.35
Packed red blood cells, median (IQR), units	0.00 (0.00-1.50)	0.00 (0.00-1.00)	.93
Received packed red blood cells	115 (34.3)	59 (35.5)	.87
Transport time, median (IQR), min	41.00 (32.25-52.00)	42.50 (35.00-53.00)	.19
Transferred from facility	77 (23.1)	34 (20.6)	.60
Hospital			
International normalized ratio, median (IQR)	1.20 (1.10-1.40)	1.30 (1.10-1.60)	.001
Thromboelastography values, median (IQR)			
α Angle, °	70.30 (61.90-74.90)	67.80 (59.75-73.47)	.11
Activated clotting time, s	113.00 (97.00-128.00)	121.00 (100.00-136.00)	.31
Kappa, min	1.75 (1.20-2.79)	2.08 (1.50-3.10)	.08
Maximum amplitude, mm	58.00 (49.00-64.15)	55.90 (48.85-61.15)	.11
Lysis at 30 min, %	1.40 (0.00-20.00)	2.30 (0.00-34.38)	.19
Transfusion in 24 h, median (IQR), units	4.00 (1.00-13.00)	4.00 (2.00-13.00)	.27
Packed red blood cells in 24 h, median (IQR), units	3.00 (1.00-7.50)	3.50 (1.00-7.75)	.61
Plasma in 24 h, median (IQR), units	0.00 (0.00-3.00)	0.00 (0.00-4.00)	.20
Platelets in 24 h, median (IQR), units	0.00 (0.00-1.00)	0.00 (0.00-1.00)	.12
Crystalloid fluid in 24 h, median (IQR), mL	4300.00 (2297.50-6500.00)	4725.00 (3103.00-6753.25)	.20
Vasopressors in 24 h	139 (41.5)	103 (62.0)	<.001
Outcome			
30-d mortality	65 (19.4)	77 (46.4)	<.001
24-hour mortality	47 (14.0)	45 (27.1)	.001
Multiple organ failure	192 (57.3)	109 (65.7)	.09
Intensive care unit length of stay, median (IQR), d	3 (1-10)	7 (2-14)	<.001
Hospital length of stay, median (IQR), d	8 (3-21)	10 (2-20)	.97
Ventilator duration, median (IQR), d	2 (1-6)	4 (1-10)	<.001

Abbreviations: IQR, interquartile range; TBI, traumatic brain injury.

^a^ To calculate *P* values for between-group comparisons, we performed the Pearson χ^2^ test for categorical variables, the Mann-Whitney *U* test for nonparametric, continuous variables with 2 or fewer groups, and the Kruskal-Wallis test for nonparametric, continuous variables with more than 2 groups.

A comparison of patients with and without TBI revealed no significant differences in patient demographic characteristics. However, patients with TBI were generally more severely injured, had higher incidence of prehospital intubation, were more likely to receive in-hospital vasopressors, and had higher intensive care unit length of stay, more days on mechanical ventilation, and higher 24-hour and 30-day mortality rates (Table 1). Approximately one-half of the TBI cohort (86 patients [51.8%]) sustained a subdural hematoma or hemorrhage (eTable 1 in Supplement 2), and of the patients with TBI who did not survive to 30 days, approximately one-half (42 patients [54.5%]) had a cause of death attributed to TBI (eTable 2 in Supplement 2).

Patients with TBI were balanced across the 2 trial groups, with 74 in the plasma group and 92 in the standard care group. Among patients with TBI, demographic and injury characteristics did not differ across groups of the trial. There were no significant differences in fluid administration during the duration of prehospital transport other than the plasma intervention. However, in-hospital transfusion requirements did differ across trial groups. Patients with TBI who were treated with prehospital plasma received less crystalloid fluid, vasopressors, and packed red blood cells in the first 24 hours. In addition, patients with TBI who received prehospital plasma had lower international normalized ratios (median [IQR], 1.20 [1.10-1.40] vs 1.40 [1.20-1.80]). Patients with TBI who received prehospital plasma had lower unadjusted 24-hour mortality (16.2% vs 35.9%) and 30-day mortality (35.1% vs 55.4%). These patients also had higher incidence of multiple organ failure and intensive care unit and hospital length of stay (Table 2).

**Table 2.** Comparison of Patient and Injury Characteristics and Outcomes Among Patients with Traumatic Brain Injury Across Groups of the Prehospital Air Medical Plasma Trial

Variable	Patients, No. (%)		*P* value^a^
	Standard care (n = 92)	Plasma (n = 74)	
Demographic characteristics			
Age, median (IQR), y	44.00 (25.00-59.25)	42.50 (25.25-60.25)	.80
Male	75 (81.5)	50 (67.6)	.06
Race			
White	80 (87.0)	68 (91.9)	.67
Black	5 (5.4)	3 (4.1)	
Asian	0	0	
Other	3 (3.3)	2 (2.7)	
Unknown	4 (4.3)	1 (1.4)	
Medications receiving			
Vitamin K antagonist	1 (1.8)	1 (1.7)	>.99
Antiplatelets	3 (5.3)	8 (13.8)	.22
Injury characteristics			
Injury Severity Score, median (IQR)	29 (22-37)	29 (20-41)	.92
Abbreviated Injury Severity score, median (IQR)			
Head	4 (3-5)	3 (3-4)	.33
Chest	3 (2-3)	3 (2-30)	.23
Abdomen	0 (0-2)	2 (0-2)	.26
Extremity	2 (0-3)	2 (0-3)	.41
Glasgow Coma Scale score			
Prehospital			
<8	71 (79.8)	52 (70.3)	.37
8-12	6 (6.7)	7 (9.5)	
13-15	12 (13.5)	15 (20.3)	
Emergency department			
<8	77 (84.6)	55 (76.4)	.17
8-12	2 (2.2)	6 (8.3)	
13-15	12 (13.2)	11 (15.3)	
Prehospital systolic blood pressure <70 mm Hg	49 (53.3)	33 (44.6)	.34
Spinal cord injury	11 (12.0)	6 (8.2)	.60
Blunt injury	92 (100.0)	74 (100.0)	>.99
Blunt mechanism			
Fall	9 (9.8)	5 (6.8)	.86
Motor vehicle crash			
Occupant	51 (55.4)	38 (51.4)	
Motorcyclist	20 (21.7)	15 (20.3)	
Pedestrian or cyclist	6 (6.5)	9 (12.5)	
Unknown	0	1 (1.4)	
Struck by or against	2 (2.2)	1 (1.4)	
Other	4 (4.3)	5 (7.8)	
Prehospital			
Intubation	72 (78.3)	53 (71.6)	.42
Cardiopulmonary resuscitation	9 (9.8)	3 (4.1)	.27
Crystalloid fluid, median (IQR), mL	1000.00 (0.00-1500.00)	700.00 (0.00-1287.50)	.20
Packed red blood cells, median (IQR), units	0.00 (0.00-2.00)	0.00 (0.00-1.00)	.13
Received packed red blood cells	38 (41.3)	21 (28.4)	.12
Transport time, median (IQR), min	41.00 (33.75-50.25)	45.00 (36.00-54.25)	.08
Transferred from facility	12 (13.2)	22 (29.7)	.02
Hospital			
International normalized ratio, median (IQR)	1.40 (1.20-1.80)	1.20 (1.10-1.40)	.001
Thromboelastography values, median (IQR)			
α Angle, °	66.10 (56.97-71.97)	69.70 (62.48-75.03)	.10
Activated clotting time, s	121.00 (97.00-136.00)	121.00 (105.00-132.00)	.97
Kappa, min	2.15 (1.56-3.50)	1.80 (1.25-2.60)	.10
Maximum amplitude, mm	55.70 (47.23-59.83)	56.00 (52.25-63.30)	.14
Lysis at 30 min, %	2.15 (0.00-40.00)	2.75 (0.00-30.00)	.63
Transfusion in 24 h, median (IQR), units	5.00 (2.00-16.00)	3.00 (0.00-10.00)	.03
Packed red blood cells in 24 h, median (IQR), units	4.00 (2.00-8.00)	3.00 (0.00-6.00)	.03
Plasma in 24 h, median (IQR), units	0.00 (0.00-4.00)	0.00 (0.00-3.00)	.34
Platelets in 24 h, median (IQR), units	0.00 (0.00-1.00)	0.00 (0.00-1.00)	.47
Crystalloid fluid in 24 h, median (IQR), mL	4999.00 (3850.00-7000.00)	4312.50 (2280.75-5867.50)	.05
Vasopressors in 24 h	67 (72.8)	36 (48.6)	.002
Outcome			
30-d mortality	51 (55.4)	26 (35.1)	.01
24-hour mortality	33 (35.9)	12 (16.2)	.008
Multiple organ failure	52 (56.5)	57 (77.0)	.009
Intensive care unit length of stay, median (IQR), d	6 (1-12)	8 (3-15)	.04
Hospital length of stay, median (IQR), d	8 (1-19)	13 (6-20)	.05
Ventilator duration, median (IQR), d	4 (1-9)	5 (2-11)	.12

Abbreviation: IQR, interquartile range.

^a^ To calculate *P* values for between-group comparisons, we performed the Pearson χ^2^ test for categorical variables, the Mann-Whitney *U* test for nonparametric, continuous variables with 2 or fewer groups, and the Kruskal-Wallis test for nonparametric, continuous variables with more than 2 groups.

We next evaluated 30-day mortality across patients with and without TBI using a generalized estimating equations model to account for trial cluster effects. The plasma and TBI association was statistically significant even after accounting for multiple confounders, including age, head AIS score, sex, Injury Severity Score, prehospital resuscitation fluid requirements, mechanism of injury, severe prehospital shock (systolic blood pressure *<*70 mm Hg), prehospital Glasgow Coma Scale (GCS) score, and transport time (eTable 3 and eTable 4 in Supplement 2).

**Figure 2.**
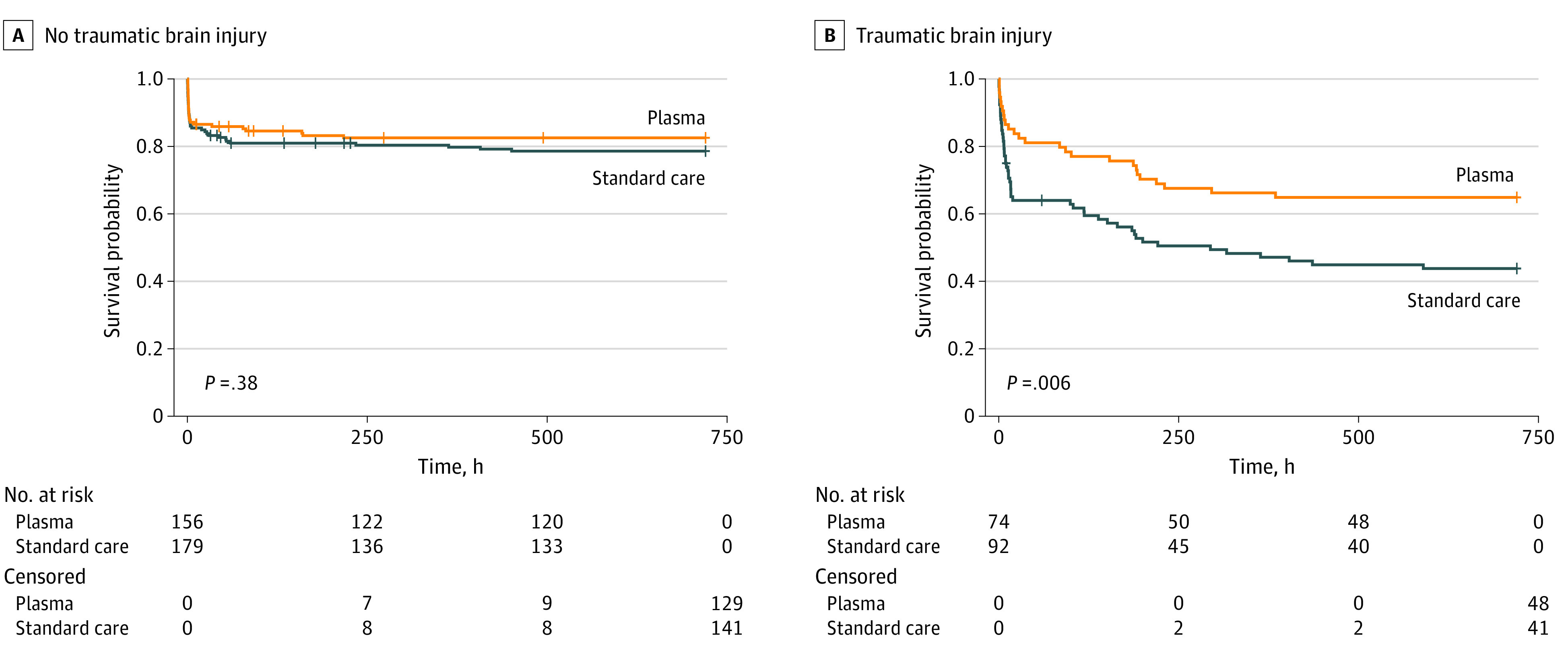
Unadjusted Kaplan-Meier Survival Analysis for 30-Day Survival Comparing Prehospital Plasma and Standard Care Patients With and Without Traumatic Brain Injury *P* values were calculated with log-rank testing.

We then performed Kaplan-Meier survival analysis comparing prehospital plasma patients vs standard care across subgroups with and without TBI (Figure 2). In the cohort of patients without TBI, there was no difference in 30-day survival across groups of the trial (HR, 0.80; 95% CI, 0.49-1.30; *P* = .38). In the cohort of patients with TBI, patients who received prehospital plasma had improved 30-day survival (HR, 0.52; 95% CI, 0.33-0.84; *P* = .006).

After analysis of 30-day survival with the use of an adjusted Cox proportional hazard model, the association between survival and prehospital plasma in the TBI subgroup remained statistically significant (HR, 0.55; 95% CI, 0.33-0.94; *P* = .03), representing a 45% lower risk of mortality (eTable 5 in Supplement 2). When patients without TBI were similarly analyzed, no significant association with survival was found for the plasma variable (HR, 0.67; 95% CI, 0.39-1.14; *P* = .14).

We assayed blood samples from patients for key markers of TBI. GFAP and UCH-L1 levels were both associated with TBI at 0 and 24 hours from admission (eFigure 1 in Supplement 2), indicating their utility as diagnostic markers of TBI. However, there was no difference in GFAP or UCH-L1 levels across groups of the trial (eFigure 2 in Supplement 2), suggesting no underlying differences in brain injury across trial groups.

**Figure 3.**
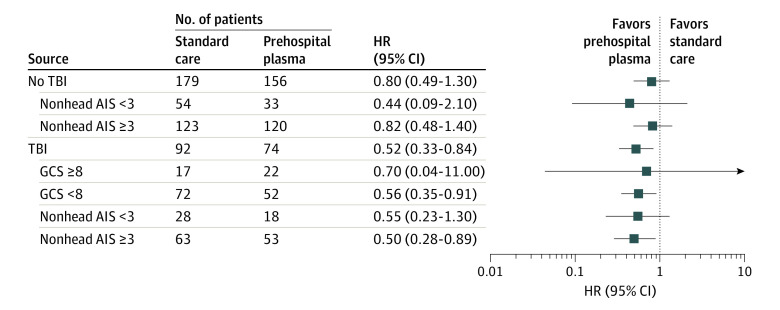
Hazard Ratios (HRs) for Each Subgroup Derived From a Cox Proportional Hazard Model HRs are reported for prehospital plasma compared with standard care. HRs less than or equal to 1 indicate a decreased risk of death; HRs greater than 1 indicate an increased risk of death. Traumatic brain injury (TBI) with mild to moderate traumatic injury in other body regions is defined by the presence of TBI and Abbreviated Injury Severity (AIS) score less than 3 in nonhead AIS categories (abdomen, chest, and extremity). Polytrauma with TBI is defined by the presence of TBI and AIS score of 3 or higher in the abdomen, chest, or extremity categories. GCS indicates Glasgow Coma Scale.

In an exploratory subgroup analysis of patients with TBI, we assessed whether initial neurological deficits or concomitant injury burden were associated with the survival benefit of plasma. We found that prehospital plasma was associated with the greatest survival benefit among patients with TBI with GCS score less than 8 (HR, 0.56; 95% CI, 0.35-0.91), for a 44% reduction in the risk of mortality, and among patients with TBI and polytrauma (HR 0.50, 95% CI 0.28-0.89), for a 50% reduction in the risk of mortality (Figure 3).

We also assessed the importance of transport origin as a proxy for the time between plasma administration and time of injury in the prehospital environment. In the cohort of patients transferred from an outside hospital, there was no difference in 30-day survival across groups of the trial (HR, 1.00; 95% CI, 0.33-3.00; *P* = .99) (eFigure 3 in Supplement 2). In the cohort of patients who were transported from the scene of injury, patients who received prehospital plasma had improved 30-day survival (HR, 0.45; 95% CI, 0.26-0.80; *P* = .005), suggesting that minimizing the time between injury and plasma administration is important.

## Discussion

Damage-control resuscitation, including prioritization of the early administration of blood products, improves survival.^8,9,33,34^ Prehospital resuscitation with blood products may prevent downstream complications of traumatic injury^8,35^ and may have implications for mitigating the secondary injury following TBI.^10^ The results of this analysis demonstrate a strong survival benefit associated with prehospital plasma in patients with TBI. This was more robust after adjustment and demonstrated a 45% lower risk of mortality for patients who received prehospital plasma. This secondary analysis suggests that patients with TBI who are administered prehospital plasma have greater survival than the overall PAMPer cohort. A persistent and guiding tenet of TBI treatment is the prevention of hypotension and secondary brain injury.^11,18,19,20,36^ Although the underlying mechanisms for the survival benefit associated with plasma remain unknown, these findings suggest that early resuscitation with blood products including plasma may benefit patients with TBI and risk of hemorrhagic shock.

Improvements in TBI care have primarily focused on the in-hospital setting.^37^ Our results suggest that the benefit of interventions may be even more robust if delivered in the prehospital setting, as close to the time of injury as feasible. Early plasma resuscitation may act by attenuating endothelial damage and inflammation,^13,14,15,38,39,40^ systems affected by the complex molecular derangements following TBI. Evidence from 2 retrospective studies^41,42^ suggests a survival benefit associated with early in-hospital plasma administration among patients with TBI. The postulated benefits of plasma in patients with burn injuries include a restoration of intravascular volume and reduction of burn injury–associated endotheliopathy.^43^

The association between prehospital plasma and survival may depend on the severity of TBI and the presence of concomitant polytrauma. Preliminary data from an exploratory subgroup analysis suggest that patients with impaired neurological function (GCS score *<*8) who received prehospital plasma had an associated 44% reduction in the risk of mortality (HR, 0.56; 95% CI, 0.35-0.91). Our subgroup analysis also reveals that receipt of prehospital plasma among patients with TBI with polytrauma (concomitant injury burden) and the inherently higher risk of hemorrhage was associated with a 50% reduction in the risk of mortality (HR, 0.50; 95% CI, 0.28-0.89). However, these results are exploratory and should be assessed in a larger, more appropriately powered trial. These preliminary findings are nonetheless consistent with the hypothesis that prehospital plasma improves survival by minimizing the detrimental effects of shock and endothelial injury, as well as reducing the effects of secondary brain injury.^44^ During both prehospital and emergency department resuscitation, injured patients often receive large volumes of crystalloid fluid. Although this may improve perfusion, it may worsen inflammation, coagulopathy, endotheliopathy, and tissue oxygenation. Another benefit of early plasma administration may be a reduced reliance on crystalloid resuscitative fluid. Our exploratory analyses suggest that patients transported directly from the scene derived a greater benefit from prehospital plasma than those transferred from other hospitals. Origin of transport (from the scene or referral emergency department) may represent a surrogate for the interval between injury and plasma intervention. Although other confounding variables may contribute to these benefits of plasma, the greatest benefit was associated with plasma administration early after injury.

Mortality from both hemorrhage and TBI occurs early after injury but follows a different time course for each cause. Hemorrhage causes most early (*<*6 hours of hospital admission) deaths, after which the proportion of deaths due to TBI dramatically increases.^45^ The survival curves for the plasma and standard care groups of patients with TBI separate prior to 6 hours following hospital admission, indicating that plasma appears to have beneficial effects early in the pathophysiology of polytrauma with TBI.^45^ Although there was no apparent difference in thrombelastography parameters across groups, patients with TBI did have reduced international normalized ratio values, consistent with the overall study findings.^8^ Future studies are needed to determine the mechanisms behind this survival benefit. Nonetheless, the finding that improved survival is associated with prehospital plasma is a critical first step in optimizing resuscitation for hypotensive patients with TBI with severe injuries.

Circulating markers of brain injury (GFAP and UCH-L1) measured at hospital admission and 24 hours after admission corroborated the CT findings and are an objective means for TBI diagnostics, as previously reported.^24^ TBI marker concentrations also suggested no underlying brain injury differences across groups of the trial. This is the first analysis, to our knowledge, to use these biomarkers in this capacity.

In this analysis, patients without TBI did not have a plasma-associated survival benefit. Although it is possible that the benefit of plasma is only for patients with TBI, study limitations or differences between these groups may also contribute to this result. Patients with TBI had a greater incidence of polytrauma, greater injury severity, and overall greater mortality compared with patients without TBI. However, in our analysis, the plasma variable was significant even after accounting for differences in patient and injury characteristics.

Overall, prehospital plasma was associated with improved 30-day survival among patients with TBI. We observed a greater incidence of multiple organ failure and longer intensive care unit and hospital length of stay in the plasma group, suggesting that patients who have a survival benefit are also more likely to develop organ dysfunction or have a longer recovery time. This could result from adverse events associated with prehospital plasma administration. However, these findings may represent a survival bias. We interpret these results to mean that plasma reduced mortality for a group of unexpected survivors, patients who would have died had they not received this intervention.^46^ These patients may also require more aggressive postinjury care. Similarly, the observed association between prehospital packed red blood cells and mortality may be due to the fluid intervention. Alternatively, we hypothesize that more severely injured patients are more likely to receive prehospital packed red blood cells, consistent with the primary analysis.^8^

### Limitations

Several limitations are inherent to a secondary analysis of a prospective, randomized clinical trial and should be considered in the interpretation of our results. First, the study was not blinded, and CT was used to diagnose TBI, factors that may result in unanticipated bias. Misclassification or misdiagnosis of TBI are potential limitations of this study. The limitations of sampling and variability in prehospital and in-hospital factors (treatment before the arrival of emergency medical services, prehospital times, and physician-level differences) also introduce bias. In addition, this study was not randomized across groups with and without TBI, and patients who received prehospital plasma also received less crystalloid fluid and blood component products. To address these issues, we adjusted for possible confounding variables across groups including patient and injury characteristics. We did not adjust for multiple comparisons. We are unable to determine whether procedures (eg, medication administration) or non-TBI factors (eg, hypotension or alcohol use) influenced variables in our analysis such as GCS score. However, we use the earliest (initial, prehospital) GCS scores as a proxy for brain injury severity. Our analysis may be underpowered to detect differences in small subgroups (eg, TBI with mild traumatic injury to other body regions) or to suggest that plasma has no beneficial effects for patients without TBI. Despite being smaller, the TBI subgroup had a greater survival benefit associated with prehospital plasma. The Cox proportional hazard model is adjusted for baseline covariates; however, there is a risk of residual imbalance and unmeasured confounding. There may be unknown differences across groups with and without TBI that are not accounted for in the current analysis. However, our biomarker results suggest that these findings do not result from major head injury differences. Although these markers did not differ across groups of the trial, this does not exclude potential differences in other unmeasured variables. In the primary trial, the time of injury was unable to be defined; thus, there may be variable time of randomization to time of injury. We quantified the time of randomization as a proxy for early or late prehospital plasma administration by transport origin and assume that there was a longer time interval if transferred from an outside hospital. During prehospital transport from the scene of an accident, without the benefit of CT imaging, we are unable to determine whether a patient has a brain injury. Although patients with TBI who received plasma have a higher frequency of multiple organ failure and longer length of stay, this may be due to survival bias. The application of these results may be limited by the fact that AIS score, Injury Severity Score, and head CT imaging are not available in the prehospital setting. Furthermore, this study was not designed to assess long-term functional neurological outcomes, although this may be an important area of future investigation.

## Conclusions

Early administration of prehospital plasma to patients with TBI is associated with improved survival, particularly among those with polytrauma and risk of hemorrhagic shock. Future studies are needed to confirm the clinical benefits of early plasma resuscitation. Our results are exploratory, but the prehospital setting may be a critical time to intervene in the care of patients with TBI.

## Notes

Supplement 1. **Trial Protocol**

Supplement 2. **eAppendix.** Supplemental Methods **eTable 1.** Traumatic Brain Injury (TBI) Subtypes **eTable 2.** Causes of Death for Patients With CT-Positive Traumatic Brain Injury (TBI) **eTable 3.** Generalized Estimating Equations (GEE) Model Coefficients Without Adjustment for Confounders **eTable 4.** Generalized Estimating Equations (GEE) Model Coefficients **eTable 5.** Cox Proportional Hazard Model for 30-Day Mortality Among Patients With TBI **eFigure 1.** TBI Biomarker Concentration in Patients With and Without TBI at 0, 24, and 72 Hours Post Admission **eFigure 2.** TBI Biomarker Concentration Compared Across the Trial Arms at 0, 24, and 72 Hours Post Admission **eFigure 3.** Unadjusted Kaplan Meier 30-Day Survival Analysis Comparing Plasma and Standard Care Arms Among Patients With TBI and Grouped by Transport Origin **eReferences**

Supplement 3. **Data Sharing Protocol**
