# Supplementary material for: Association of Prehospital Plasma With Survival in Patients With Traumatic Brain Injury: A Secondary Analysis of the PAMPer Cluster Randomized Clinical Trial
Source: JAMA Netw Open. 2020 Oct 15;3(10):e2016869. doi: 10.1001/jamanetworkopen.2020.16869 (PMC7563075; doi:10.1001/jamanetworkopen.2020.16869)

Clinical Protocol

University of Pittsburgh

Departments of Critical Care, Surgery and Emergency Medicine
MACRO (Multidisciplinary Acute Care Research Organization)

Prehospital Air Medical Plasma (PAMPer)
Phase III Multicenter, Prospective, Randomized, Open-label, Interventional Trial
Protocol Version 3 date: January 17, 2017

Funding for the study is provided by the Department of Defense (DOD) Telemedicine and Advanced Technology Research Center (TATRC).

IND Sponsor: **Jason L. Sperry MD, MPH**
Assistant Professor of Surgery and Critical Care Medicine
University of Pittsburgh Medical Center
3500 Fifth Avenue, Suite 303
Pittsburgh, Pa. 15213
412 802 8270
sperryjl@upmc.edu

Table of Contents

I. Study Site Investigator Information ... 4
Investigator, University of Pittsburgh/UPMC ... 4
Sub-Investigators, University of Pittsburgh/UPMC ... 4
Investigators at other participating institutions/study sites ... 4
Medical Monitor ... 4

II. Scope of Work ... 5

III. Roles and Responsibilities ... 5
Study timelines ... 5
Pre-trial and Year one ... 5
Year Two ... 6
Year Three ... 6
Year Four ... 6

IV. Study Site Information ... 6

V. Study Information ... 7

VI. Study Design ... 7
Background and significance ... 7
Preliminary studies ... 10
Objectives and hypothesis ... 12

VII. Research Design and Methods ... 15
Study design and setting ... 15
Study population ... 15
Inclusion and exclusion criteria ... 15
Randomization ... 15
Intervention ... 16
Standard of Care ... 17
Standard Operating Procedures (SOPs) ... 17
Blinding ... 18
Outcome Variables ... 19
Primary Outcomes ... 19
Secondary Outcomes ... 19
Clinical Outcomes ... 19
Twenty four hour blood transfusion requirements ... 19
In-hospital Mortality ... 19
Multiple Organ Failure ... 19
Nosocomial Infection ... 20
Acute Lung Injury ... 20
Blood component transfusion requirements ... 20
Coagulation Parameters ... 21
Cytokine and Protein C pathway measures ... 21

VIII. Human Subjects ... 21
Screening and enrollment ... 22
Informed consent and notification ... 22
HIPAA ... 23

IX. Sample Storage 23

X. Data 23

XI. Analysis plan 24
Primary hypothesis 24
Secondary hypothesis 25
Predefined subgroup analysis 26
Randomization of ineligible subjects 26
Non- adherence 26
Sample size justification and power analysis 26

XII. Safety monitoring 29
Adverse events 29
Data Safety Monitoring Board (DSMB) 32
Interim analysis 33
Quality control, assurance and confidentiality 35

XIII. Study limitations 35

XIV. Timetable 36

XV. Additional regulatory requirements USAMRMC 36

XVI. Literature Review 36

Clinical Protocol -Appendix 1: Response to Requirements for Exception From Consent For Emergency Research 43

Clinical Protocol -Appendix 2: Reporting Requirements for USAMRMC HRPO 46

Clinical Protocol -Appendix 3: Call Tracker for LAR – Incoming/Outgoing 47

Clinical Protocol -Appendix 4: Blood Product Maintenance 49

Clinical Protocol -Appendix 5: Donor-Patient "Lookback" 54

Clinical Protocol -Appendix 6: Data Safety Monitoring Board (DSMB) Overview 60

Clinical Protocol -Appendix 7: Reporting Requirements 67

Clinical Protocol - Appendix 8: Harmonized Protocol 68

Clinical Protocol - Appendix 9: PAMPer Roles and Responsibilities 85

I. Investigator Information

A. Investigators at the University of Pittsburgh/UPMC:

Jason L. Sperry MD, MPH
Assistant Professor of Surgery and Critical Care Medicine
University of Pittsburgh Medical Center
3500 Fifth Avenue, Suite 303
Pittsburgh, PA 15213
412 802 8270
sperryjl@upmc.edu

B. Sub-Investigators and Key Study Personal at the University of Pittsburgh/UPMC:

Frank X. Guyette MD, MS, MPH, Assistant Professor of Emergency Medicine, University of Pittsburgh
Darrell J. Triulzi MD, Professor of Pathology and Medicine, University of Pittsburgh
Mazen Zenati MD, MPH, PhD, Assistant Professor of Surgery and Epidemiology, University of Pittsburgh
Timothy R. Billiar MD, Chair, Department of Surgery, University of Pittsburgh
Derek Angus MD, MPH, Chair, Department of Critical Care Medicine, University of Pittsburgh
Andrew B. Peitzman MD, Vice-Chair,Chief, Division of General Surgery, University of Pittsburgh
Clifford Calloway MD, PhD, Associate Professor of Emergency Medicine, University of Pittsburgh
Mark Yazer, MD, Medical Director Centralized Transfusion Service**;** University of Pittsburgh
Barbara Early, RN, BSN, **CCRC**, MACRO CRC Director, University of Pittsburgh
A. Tyler Putnam, MD, Trauma and Acute Care Surgeon, Trauma and Emergency Services, UPCM Altoona

C. Investigators at other participating institutions:

**Case Western University -**	**Jeffrey A. Claridge, MD, MS**
**University of Louisville -**	**Brian G. Harbrecht, MD**
**University of Texas Southwestern-**	**Herb Phelan, MD**
**University of Tennessee-**	**Brian M. Daley, MD**
**Vanderbilt University-**	**Richard S. Miller, MD, FACS**
**Harris Methodist Hospital-**	**William Witham, MD**
**John Peter Smith Hospital-**	**Therese Duane, MD**

D. Research Monitor (Coordinating Center – University of Pittsburgh):

Louis Alarcon, MD
Medical Director, Trauma Surgery
F 1264 PUH
200 Lothrop St.
University of Pittsburgh
Pittsburgh, Pa 15213
412 647 1158
alarconl@upmc.edu

II. Scope of Work

A. The IND Sponsor and study site Investigators will through the execution of the trial:

1. Determine whether prehospital infusion of 2 units of AB plasma or low titer anti-B A plasma as compared to standard air medical care results in a reduction in 30 day mortality

2. Determine whether prehospital infusion of AB plasma or low titer anti-B A plasma as compared to standard air medical care results in a lower incidence of 24 hour transfusion requirements, in-hospital mortality, multiple organ failure, nosocomial infection and acute lung injury.
3. Determine whether prehospital infusion of AB plasma or low titer anti-B A plasma as compared to standard air medical care results in a reduction of blood component transfusion and resuscitation requirements over the first 24 hours post-injury.
4. Determine whether prehospital infusion of AB plasma or low titer anti-B A plasma as compared to standard air medical care results in improved coagulation measurements as determined by INR, PT, and thromboelastography (TEG) parameters.
5. Determine whether prehospital infusion of AB plasma or low titer anti-B A plasma as compared to standard air medical care results in lower levels of early IL-6 cytokine expression, reduced thrombomodulin and increased protein C levels.

III. Roles and Responsibilities

A. IND Sponsor: Dr. Jason L. Sperry will oversee all planning and execution of the Prehospital Air Medical Plasma (PAMPer) trial, which is a 4 year, multi-center, randomized, open label, clinical trial, and will assume the responsibilities of the IND Sponsor as addressed under 21 CFR Part 312, Subpart D.
B. Study Site Investigators: The study site Investigators will oversee the conduct of the PAMPer trial at their respective study sites, and will assume the responsibilities of Investigators as addressed under 21 CFR Part 312, Subpart D.
C. Coordinating Center: The University of Pittsburgh will serve as both the clinical outcome and data-coordinating center. The University of Pittsburgh Coordinating Center, under the auspices of the IND Sponsor, will be responsible for the education and training of participating center research staff and will oversee education and training of prehospital providers from participating centers. The University of Pittsburgh Coordinating Center, under the auspices of the IND Sponsor, will be responsible for sample acquisition, sample storage, data entry via web based platform, and maintenance of data integrity.
D. Study Timeline Responsibilities:
1. Pre-Trial Start Period and Year One:
University of Pittsburgh Coordinating Center: The IND Sponsor, participating study site Investigators, consultants, research staff and data management team will develop the clinical trial protocol, Investigator's Brochure, informed consent documents and notification letters, data collection forms, database, and manual of operations. An IND application will be submitted to the FDA in accordance with the provisions governing the conduct of this clinical trial under the Exception from the Requirement for Informed Consent for Emergency Research. A coordinating center protocol and separate study site clinical protocol will be initially submitted for review and approval by the University of Pittsburgh IRB, and the USAMRMC Office of Research Protections Human Research Protections Office. Once approved at these levels, the University of Pittsburgh Coordinating Center will send materials to subcontracted study sites for submission to their respective IRBs. Study sites will be added to the clinical trial protocol following approval by their responsible IRB, and the IND application and Coordinating Clinical Center protocol will be amended accordingly.

Participating Study Sites: Study site Investigators will coordinate and oversee the execution of the clinical trial at their respective institutions. They will finalize plans with each of their respective blood banks for the obtaining and rotation of plasma to and from the helicopter bases. They will each submit their plasma plan to the University of Pittsburgh Coordinating Center for approval. Once they have received an approved master clinical trial protocol, the study site investigators and their research team will submit the protocol to their respective IRB's and follow local and federal regulations and guidelines relevant to this research; i.e.: 21 CFR 50.24, Exception from Informed Consent Requirements for Emergency Research, the harmonized U.S. Department of Health and Human Services (HHS) regulations; 21 CFR Part 312, Investigational New Drug Application; and DOD Directive DODD 3216.02. No research at any site will commence until approval from the responsible IRB and the USAMRMC ORP HRPO has been received.

2. Year Two:

University of Pittsburgh Coordinating Center staff will conduct a study site initiation visit subsequent to responsible IRB approval of the clinical trial protocol and approval of the study site by the USAMRMC ORP HRPO. The Coordinating Center research staff will verify that the study team has received and reviewed the protocol, and also understands the relevant scientific background information. We will go over the study time line and accrual rate. Roles and responsibilities of all key personnel will be reviewed, along with a site delegation of duties log. We will review their ability to conduct the study according to the written protocol, federal and DOD regulations. We will ascertain their understanding of adverse event reporting and serious adverse event reporting. So as to address the training of study site staff who were unavailable at the time of the site initiation visit or who become involved at a later date, the Coordinating Center will implement a web-based training module. This will include testing which will serve as documentation that training requirements have been met.

Following a training period for participating study sites on enrollment procedures and TEG analysis, trial enrollment at the study site will begin. Blood samples will be batched for analysis, and prospective outcomes data will be entered and integrity verified. It is anticipated that 110 subjects will be enrolled in the first year. After the first 100 subjects have completed the clinical trial protocol, an initial interim analysis with be completed with a focus on safety.

Phase system analysts at the University of Pittsburgh Coordinating Center will create a web-based secure server to link sites with relevant information; a pass-word protected, electronic Case Report Form for data entry; and training modules for the conduct of the clinical trial protocol including electronic data entry. Personnel will need to pass a test on the portal as evidence of their training and knowledge of the protocol and procedures.

Each enrolling study site will be monitored on an annual basis by the research staff of the University of Pittsburgh Coordinating Center. Study sites having difficulty with addressing the provisions associated with the Exception from the Requirement for Informed Consent for Emergency Research, underperforming sites, or sites with multiple protocol deviations will be reassessed for ability to continue. If unable to improve, they may be replaced.

Two formal interim analyses of efficacy will be performed when 33% and 67% of the expected number of primary events had accrued (about one month after 1/3 and 2/3 of subject accruals).

An estimated additional 150 patients will be enrolled (total=260) by end of second year, and blood samples will be batch analyzed for cytokine and protein C constituents. Continued prospective data collection and integrity verification for clinical outcomes will occur.

3. Year Three:

An estimated additional 150 patients will be enrolled (total=410) by end of third year, and blood samples will be batched analyzed for cytokine and protein C constituents. Continued prospective data collection and integrity verification for clinical outcomes will occur.

4. Year Four:

An estimated additional 120 patients will be enrolled (estimated total= 530) by 9 months into the fourth year, with completion of analyses for cytokine and protein C constituents once we meet our enrollment. Prospective data collection will be completed and final integrity verification for all data will occur. Final data analysis will be completed in the final 3 months after data integrity is verified. Manuscript preparation will follow soon after.

IV. Study Site Information

It is anticipated that the participating study sites/institutions will include: University of Pittsburgh/UPMC, University of Texas Southwestern, University of Tennessee, Case Western Reserve University, University of Louisville and Vanderbilt University. These institutions have busy air medical transport services and existing affiliations with local blood banks. The actual intervention will take place en route to the trauma center, and follow up data will be collected up to 30 days, independent of discharge disposition.

V. Study Information

The study will be a 4 year, multi-center, open-label, randomized trial conducted at level-1 trauma centers. For patients with evidence of hemorrhagic shock being transported by air medical transport, the pre-hospital infusion of two units of AB plasma or low titer anti-B A plasma (<1:50 dilution or titer <50) will be compared to air medical standard of care.

VI. Study Design

A. Background and Significance:

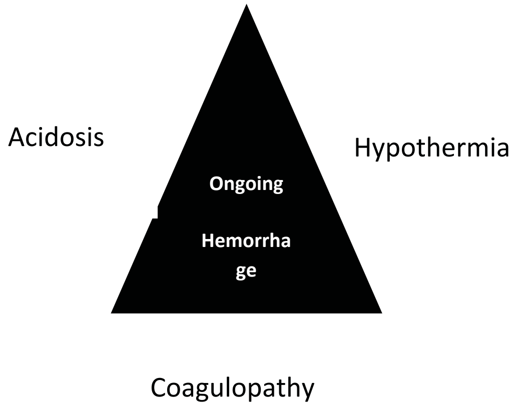

Fig 1. Adapted from Jansen JO, et al. BMJ. 2009 Jun 5;338:b1778

1. Uncontrolled hemorrhage and coagulopathy remain leading causes of mortality post-injury: Hemorrhage is estimated to be responsible for over 40% of all trauma-related deaths, nearly half of which occur in the pre-hospital setting.[1, 2] In addition, uncontrolled bleeding remains the leading cause of early in-hospital mortality.[3, 4] Ongoing hemorrhage is complicated by the well-known ‘lethal triad’ of coagulopathy, hypothermia and acidosis (Fig 1.).[5-8] It has been demonstrated that persistent hypothermia and progressive metabolic acidosis are associated with severe recalcitrant coagulopathy and resultant unbridled hemorrhage.[9-12] Although multiple mechanisms which promote or result in coagulopathy post-injury have been proposed and studied, interventions that reduce the morbidity and mortality associated with hemorrhage and coagulopathy in the clinical arena remain limited.[13, 14]

2. Coagulopathy is common, occurs early and is a complex, primary process following injury: Coagulopathy has been shown to be present in over 25% of patients at the time of trauma admission and has been determined to be an independent predictor of mortality with an associated 4-fold higher risk of mortality in both civilian and military settings.[15-19] Those injured who arrive with coagulopathy also have been shown to have longer ICU stays and ventilator requirements, are more likely to develop acute renal injury and multiple organ failure, and have a trend towards a greater incidence of acute lung injury.[18, 20] Prior literature has suggested that the coagulopathy which complicates injury is a secondary event driven by physiologic derangements and abnormalities.[7, 9, 21] Postulated mechanisms for this post-injury coagulopathy include dilution, depletion, and disseminated intravascular coagulation (DIC).[22] Dilution is thought to arise secondary to excessive crystalloid or colloid, or due to transfusion of blood products devoid of coagulation factors; with depletion resulting from ongoing bleeding where factor replacement lags behind utilization.[22] In a smaller subgroup of trauma patients, DIC has been shown to occur and is associated with the systemic inflammatory response syndrome and multiple organ failure.[23] Evolving evidence suggests that these prior mechanisms, which drive dysfunction or consumption of coagulation factors, may be individually too simplistic.[24] More recent evidence demonstrates the importance of shock and tissue hypoperfusion as principal drivers of coagulopathy following injury which may be required for coagulation factor

Fig 2. Nasciento B. et al. Crit Care. 2010; 14(1): 202.

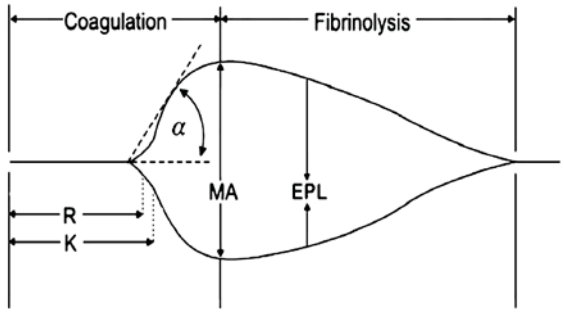

Fig 3. Standard TEG parameters. Reaction (R) time, clot formation (K) time, fibrin cross-linking (angle = α), clot strength (maximal amplitude [MA]), and estimated percent lysis (EPL). Harr JN,et al. J Surg Res. 2011 Apr 17 [Epub ahead of print]

dilution and depletion to become evident.[12, 20, 24, 25] (Fig 2.) These processes may in part be modulated by the thrombomodulin-protein C pathway.[20] As our understanding has increased regarding the mechanisms responsible for the acute coagulopathy of trauma, a new paradigm where early coagulopathy post-injury is considered a complex, multi-factorial, primary event has developed.[22, 24, 26]
It is with this understanding that the scope and magnitude of morbidity due to uncontrolled hemorrhage is demonstrated, highlighting the importance of the potential benefits of prehospital administration of plasma which may improve both tissue hypoperfusion and lessen or prevent the early coagulopathy and resultant transfusion requirements which complicates severe injury and lower 30 day mortality.

3. Diagnosis of the acute coagulopathy of trauma: As we continue to expand our understanding of the acute coagulopathy of trauma, emphasis has also been placed on diagnosing coagulopathy which complicates injury to allow real time assessment to guide evolving blood component transfusion requirements.[27] Increasing evidence suggests that historic reliance on prothrombin time (PT) and international normalized ratio (INR) is time exclusive and provides insufficient information relative to the complexity which drives this coagulopathic process.[28-30] What is needed for the appropriate evaluation of an acutely injured patient's coagulation status is a rapid, reliable assessment of the thrombosis and fibrinolysis arms of the hemostatic cascade. Thrombelastography (TEG) is a technology which provides a real time, viscoelastic analysis of these blood clotting processes.[27] (Fig 3.) Point-of-care rapid thrombelastography (POC r-TEG) differs from standard TEG because the clotting process and subsequent analysis is accelerated by the addition of tissue factor to a whole blood sample.[31] POC r-TEG is limited, however, by the requirement that the analysis be performed within 4 minutes of blood draw to prevent clot formation unless the addition of citrate occurs.[31] It has been demonstrated that TEG can assess coagulopathy, platelet dysfunction and hyperfibrinolysis at an early stage following injury and is the most rapid available test for providing reliable information on coagulopathy in significantly injured patients.[32, 33] If not more important, the technology has been deemed feasible for use in a deployed military setting as well as for civilian use.[34]

4. Aggressive blood component transfusion is associated with improved outcomes in massive transfusion: Both allogeneic blood and FFP transfusions have been shown to be independent risk factors for poor outcome in the critically ill. [35-39] Despite these inherent risks, the acutely exsanguinating, injured patient at times requires large volumes of these transfusion components until definitive control of bleeding and hemostasis can be obtained.[40-42] A significant amount of recent attention has focused on the prevention and treatment of the early coagulopathy which complicates severe injury and massive transfusion.[42-45] Since 2007, a large amount of both military and civilian evidence has accumulated suggesting that ratio-based transfusion strategies targeting high fresh frozen plasma: packed red blood cell and platelet: packed red blood cell transfusion ratios reduces the morbidity and mortality associated with unbridled hemorrhage and massive transfusion post injury. [46-53] These same studies revealed significantly lower overall blood transfusion requirements with shorter time intervals to receiving individual component transfusion, when these resuscitation protocols were employed in the hospital setting.[47, 50, 54, 55] Controversy remains regarding the exact proportion of plasma or other blood components these patients with hemorrhagic shock should receive and the potential for survival bias when analyzing retrospective data[56-59]; however, consistent evidence suggests that addressing the acute coagulopathy of trauma is associated with improved outcome.[60]
This evidence demonstrates that plasma transfusion plays an intricate role in addressing the early coagulopathy which is present at the time of admission following injury and that intervening early in the prehospital setting has the potential to further reduce overall transfusion requirements and significantly improve outcomes associated with hemorrhagic shock.

5. Predicting high volume transfusion requirements: With the demonstrated benefit of targeting high plasma and platelet transfusion ratios in those patients that ultimately require massive transfusion (defined as transfusion of >10 units of pack red blood cells in the first 24 hours from injury), it is essential that massive transfusion can be predicted relatively early, soon after presentation to the trauma center in a large proportion of patients.[61] There exists an increasing pool of literature suggesting that this can be done relatively easily soon after (or before) trauma center arrival. The majority of these massive transfusion scoring systems incorporate laboratory values in addition to vital signs upon admission in both civilian and military settings.[61-

65] Consistently, these scoring systems include hypotension (<90mmHg) as one of the primary predictors of large volume transfusion requirements. The ABC scoring system consists of 4 non-weighted parameters and include hypotension (<90mmHg), penetrating mechanism, positive focused assessment sonography of trauma, and a heart rate >120 bpm.[66] This score had an area under the curve of 0.84 via receiver operation characteristic curve analysis and is devoid of any laboratory measurements or requirements.
These analyses suggest that the vital sign parameters of hypotension and tachycardia have the ability to predict with a high likelihood the requirement for those patients who ultimately require large volume transfusion, with a corresponding high propensity to develop early coagulopathy. It is this cohort of patients where the benefits of early plasma intervention may have its strongest clinical effect.

6. Risks associated with plasma transfusion: Both allogeneic blood and blood components have been shown to be independent risk factors for morbidity in the critically ill. [35-39, 47, 67] With their use a risk of allergic reactions, transfusion-associated acute lung injury, transfusion-associated cardiac overload, and acute respiratory distress syndrome, has been documented.[39, 60] These are hypothesized to be secondary to transfer of antileukocyte antibodies from allo-immunized donors or a resultant biological response to accumulated by-products associated with blood and blood component storage.[39] Consistently, these studies which have characterized the risks associated with plasma transfusion find no association with greater mortality. It is in those patients with hemorrhagic shock and early coagulopathy where the documented survival benefit likely far exceeds any complication risks attributable to component transfusion.[47, 60, 67, 68]
The risks associated with plasma transfusion may inherently be minimized secondary to addressing and intervening with the development of acute coagulopathy post injury early in the prehospital setting and by reducing overall blood transfusion requirements may result in a reduction of 30 day mortality.

7. Universal donor 'AB- plasma':
For this clinical trial, the early, pre-hospital administration of non-cross-matched plasma will utilize a rare resource, 'universal donor plasma' or AB plasma represents approximately 3-5% of all plasma available and is typically considered in chronic shortage.[25] It is sometimes referred to as 'liquid gold'. Due to the precious nature of universal donor plasma, the NIH blood bank has developed and maintains a special AB plasma donor program. (http://clinicalcenter.nih.gov/blooddonor/donationtypes/ab_plasma.html)

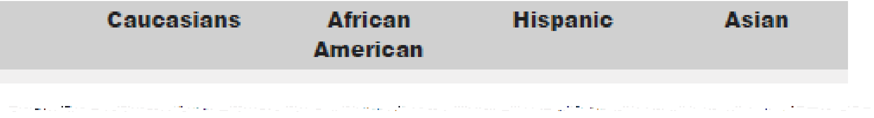

Fig 4. http://www.redcrossblood.org/learn-about-blood/blood-types

It is the precious nature of universal donor plasma which highlights the importance of utilizing air medical transport in this proposal. Air medical services cover large geographical areas and provide care at both the scene of injury and to those critically injured patients who are initially evaluated at outside facilities and require transfer to definitive care trauma centers. By utilizing air medical services as the site of universal donor plasma administration, the most efficient use of a rare resource can be accomplished with minimization of waste. Utilizing trauma centers with busy air medical services, who have relationships with their respective blood bank affiliates, will allow the successful completions of the aims and objectives proposed with minimization of blood bank resources due to the logistical considerations relative to ground prehospital transport.

8. Delay to definitive hemorrhage control: Definitive control of ongoing hemorrhage remains a fundamental principle in trauma management. Increasing attention has been paid to the significance of delay and the timing of definitive control of hemorrhage. Clarke and colleagues have previously shown that delays to operative intervention in patients with significant abdominal injuries are associated with a higher mortality risk, demonstrating a 1% higher risk of mortality for every 3 minute delay in getting patients from the ED to laparotomy.[69] Additional studies documenting relationships between delay and poor outcome following injury have been demonstrated for interventional radiology procedures and by excessive radiographic imaging post-injury in the hospital setting.[70, 71] Prehospital air medical transport has been shown to be associated with

improved outcome following severe injury, however, scene time and overall transport times are consistently longer as compared to ground transportation in both civilian and military setting.[72-76]

The results provided by the successful completion of this proposal will have paramount implications for both civilian and military injured patients as control of hemorrhage and delay to definitive care represent major impediments for both populations. This proposal will provide needed insight into the consequences of early plasma intervention in these critically injured patients when these impediments exist.

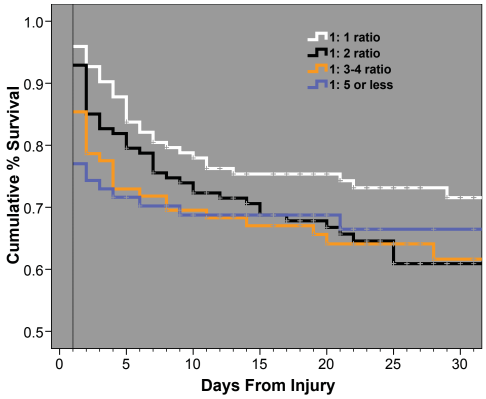

Fig 5. Kaplan-Meier Survival Analysis comparing survival across different transfusion ratio groups.

B. Preliminary Studies:

1. High plasma: blood transfusion ratios improve survival and reduce blood transfusion requirements: Secondary to the University of Pittsburgh's participation in the *Inflammation and the Host Response to Injury Large Scale Collaborative Program or 'glue grant' prospective cohort trial, (www.gluegrant.org), we have previously characterized the relationship between high fresh frozen plasma:packed red blood cell (FFP:PRBC) transfusion ratios in massive transfusion patients.[47] We verified a dose response relationship revealing that as the FFP:PRBC increased toward 1:1.5, a significant reduction in mortality occurred. (Fig 5.) These findings remained significantly robust after controlling for important differences in injury severity, temperature, shock parameters and operative interventions. Equally important, there were significant reductions in blood and blood component transfusion requirements in those with High vs. Low FFP:PRBC transfusion ratios. (Fig 6.) In a more recent analysis (unpublished, submitted to 2012 EAST) aimed to debunk any question of survival bias regarding high plasma transfusion ratios,* Cox-Hazard regression was used to determine the independent mortality risks at 6hr, 12hr, and 24hrs while controlling for important confounders. FFP:PRBC and platelet:PRBC ratios were also analyzed as time-dependent covariates accounting for fluctuation over time. We found that despite similar degrees of early shock and coagulopathy, high FFP:PRBC and platelet:PRBC ratios are associated with a survival benefit as early as 6hrs and throughout the first 24hrs, even when time dependent fluctuations of component transfusion were accounted for (Fig 7). We concluded that the observed mortality benefit associated with high component transfusion ratios was unlikely due to survivor bias and that early attainment of high transfusion ratios may significantly lower the risk of mortality in massive transfusion patients.

Fig 6. Transfusion requirements across High and Low FFP:PRBCs groups

This previous work demonstrates that in patients who ultimately require large volume transfusion, targeted high proportions of plasma improves outcome.

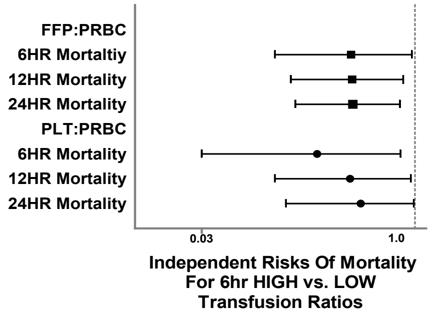

Fig 7. Mortality risks for 6hr High and Low transfusion ratios over the first 24hrs post injury.

2. Earlier more aggressive blood component transfusion is associated with a reduction in massive transfusion: We have recently characterized changes in resuscitation practice which have occurred over time in a cohort of severely injured patients requiring massive transfusion (in press, Journal of Trauma, presented at Western Trauma Association, 2011). We demonstrated that the incidence of massive transfusion (>10 units blood) significantly decreased over time, despite the median ISS of the cohort increasing. (Fig 8). When the recent time

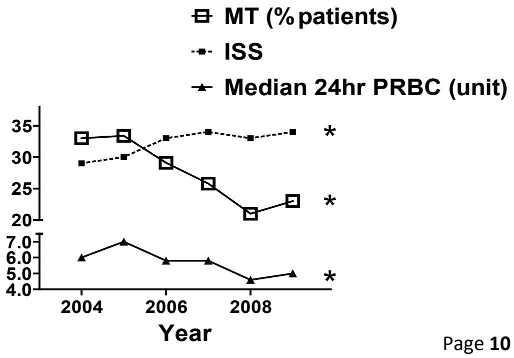

Fig 8. Decreasing incidence of massive transfusion over time with increasing injury severity

period (2007-current) was compared to the early time period (2004-2006) of enrollment for the study, there was a significant increase in the FFP: PRBC and platelet: PRBC transfusion ratios as early as 6 hours post injury, and the proportion of each blood component that was given in first 6 hours relative to the total given at 24 hours significantly increased (Fig. 9). This occurred in patients who required 7-10 Units of blood, just below the definition of massive transfusion. The data suggests that early, more aggressive attainment of high transfusion ratios may reduce the requirement for massive transfusion and may shift overall blood requirements below those which currently define massive transfusion.

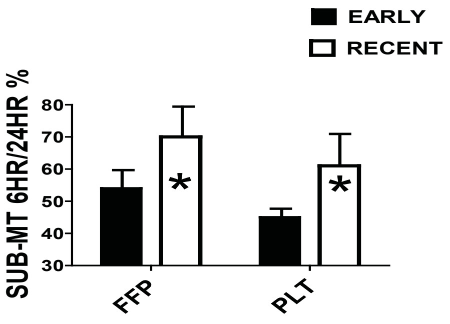

Fig 9. Comparing the proportion of component transfusion in the first 6 hours relative to 24 hours in early and recent time periods.

This previous work suggests that early and aggressive plasma administration may be associated with improved outcomes and reduced overall blood transfusion requirements and mortality.

<u>The potential risks associated with plasma transfusion:</u> We have previously documented the independent risks associated with blood component transfusion (per/Unit) in a cohort of significantly injured patients.[67] Using Cox hazard regression and controlling for all important confounders, we found no association between plasma administration and the development of nosocomial infection. There was a relationship between plasma and multiple organ failure and acute respiratory distress syndrome. A dose response relationship was documented with the risk of these complications significantly increasing after 3 Units of plasma (Fig. 10). Overall, taking into account all patients in the analysis, plasma was associated with a significantly lower independent risk of mortality. For every Unit of plasma given (in-hospital) the independent risk of mortality was estimated to be reduced by 3% (HR 0.97, p=0.02, 95%C.I. 0.94-0.99, Fig. 11

This prior work suggests the mortality benefit may outweigh the potential risks associated with plasma transfusion; with the early administration of plasma having the potential to reduce overall blood transfusion requirements and further improve outcomes. These results are similar to prior military experience which documented improved survival for every unit of plasma a patient receives.[68]

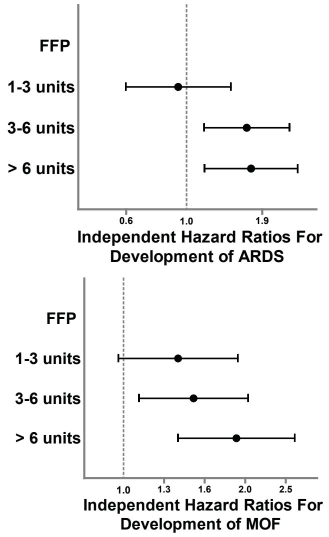

Fig 10. Dose dependent relationship between plasma transfusion and organ failure and acute respiratory distress syndrome.

<u>3. Feasibility of air medical service intervention:</u> The air medical service at the University of Pittsburgh/UPMC is the busiest non-profit flight service in the country and has a significant track record of prospective trials and interventions.[77-80] The Department of Emergency Medicine's participation in the *Resuscitation Outcomes Consortium* (https://roc.uwctc.org/) further demonstrates the expertise and capabilities this service has with air medical interventions. A recent analysis demonstrates the importance of prehospital serum lactate measurement during air medical transport for traumatic injury and its role as an independent predictor of in-hospital death, need for emergent operative intervention and the development of multiple organ failure[80] More recent work (submitted, Journal of Trauma, 2011) demonstrates the utility of air medical tissue oximetry and the ability it has to predict operative intervention or blood transfusion in the first 24 hours following injury.

Additional anticipated participating centers for this clinical trial, which include the University of Texas Southwestern, University of Louisville, University of Tennessee at Knoxville, Vanderbilt University and Case Western Reserve University, have been selected for their relationships with their respective air medical transport team, their experience with air medical interventional trials and their clinical research infrastructure to allow an interventional trial as described to occur. Air bases that will be utilized for the trial will be selected to maximize patient enrollment, minimize their distance from the trauma center and blood bank facility and to

provide the widest geographic distribution of patients. The anatomy of each respective institution will vary according to these variables and will each be individually maximized. Dr. Herb Phelan and Dr. Joseph Minei from the University of Texas Southwestern are also investigators in the *Resuscitation Outcomes Consortium* where pre-hospital interventional trials are the focus of the consortium. Similarly Dr. Harbrecht from the University of Louisville was the lead site investigator of the Inflammation and the Host Response to Injury Large Scale Collaborative Program. Dr. Harbrecht was previously at the University of Pittsburgh prior to becoming director of the division of Trauma at the University of Louisville. He leads a trauma division with a busy air medical service and with a research infrastructure already in place for the execution of this trial. Similarly, Dr. Brian Daley is the division leader of a large and busy air medical transport service at the University of Tennessee, which has a clinical research infrastructure already in place with the air medical service to allow the smooth execution of this trial. Dr. Richard Miller from Vanderbilt University leads one of the busiest air medical services in the Southeast which is operated by the Vanderbilt Hospital system itself, with the appropriate research infrastructure already in place for the execution of this trial. Finally, Dr. Jeffrey Claridge leads the trauma division at Metro Health hospital at Case Western Reserve University and is the air medical director for the entire Northern Ohio area with similar research infrastructure to allow the execution of this clinical trial.

4. Feasibility of the AB plasma or low titer anti-B A plasma **intervention:** The collaborative environment between the Departments of Surgery, Emergency Medicine and Transfusion Medicine at the University of Pittsburgh/UPMC unifies prehospital clinical research expertise with hospital based acute care research expertise and will provide the main impetus for the successful execution of the current proposal. The leadership and direction provided by University of Pittsburgh/UPMC clinicians and investigators will also promote the successful execution of the trial at the other participating centers. Dr. Triulzi, as the Medical Director of the Institute of Transfusion at the University of Pittsburgh, has the buy-in and support of Pittsburgh Central blood Bank and our transfusion service in ensuring the availability of 6 Units of AB thawed plasma at all times. This plasma will be exchanged prior to day 5 to be utilized as standard hospital supply; thereby minimizing any waste of this valuable resource. This will allow 3 out of 6 air transport bases to have 2 units of AB plasma or low titer anti-B A plasma per month at any one time. If we need to increase number of participating bases, we will work closely with Dr. Triulzi to minimize waste. Similar exchange procedures of AB plasma or low titer anti-B A plasma have been prearranged at the other participating centers where similar collaborative relationships were required to be considered as a participating center for this trial. The relationships already in place and the prior experience of each of the participating centers will allow the smooth execution of the current trial.

Thawed plasma which will be distributed to the air medical bases and returned if unused has the potential to increase the average age of the thawed plasma depending on how quickly thawed plasma is utilized at each respective center for trauma and non-trauma transfusion needs during different time periods of enrollment. Transfusion practice, due to the relative scarcity of universal donor AB plasma, dictates the use of the oldest thawed plasma available (up to 5 days) when required. As no clinically significant differences have been documented regarding the safety or efficacy secondary to the age of thawed plasma (1-5 days old), the potential for increasing the average age is possible but not clinically significant in regards to the safety of transfusion practice at each center or the execution of the proposed trial. The storage logs will be with the plasma product at all times recording age and temperature of storage. The respective transfusion services at each participating center will have full and complete access to the storage records to verify that the plasma products have been properly stored prior to their exchange. Blood bank staff will verify age and proper storage before reissuing plasma.

C. Objectives/Hypotheses:

1. Study Rationale: The effects of coagulopathy, hypothermia and acidosis are well known markers for mortality following traumatic hemorrhage. Increasing attention has recently been paid to the correction of the coagulopathy which complicates damage control resuscitation. Importantly, coagulopathy has been shown to be present very early after injury, at the time of trauma admission, even further substantiating the importance of early initiation of treatment. It is with this understanding that the magnitude of importance of the current proposal becomes apparent. Air medical transport is utilized for both civilian and military injured victims where delay to definitive care and hemorrhage control is exceedingly common. Delay to definitive care and hemorrhage control has been shown to be associated with poor outcome. It is in this cohort of patients where

interventions that improve or prevent coagulopathy may have their greatest positive effect. The successful completion of the proposed aims will provide needed insight into the potential consequences of early intervention in these critically injured patients.

2. Primary Objective: To determine the effect of the prehospital infusion (i.e., during air medical transport) of AB plasma or low titer anti-B A plasma (2 units) on 30 day mortality in patients with hemorrhagic shock as compared to standard air medical care.

a. Primary Aim #1: Determine whether prehospital infusion of AB plasma or low titer anti-B A plasma (2 units) as compared to standard air medical care results in a reduction in 30 day mortality
***Hypothesis Primary Aim #1*:** Patients in hemorrhagic shock who receive AB plasma or low titer anti-B A plasma during air medical transport will have a reduced 30 day mortality as compared to patients who receive standard air medical care.
3. Secondary Objectives: To determine the effect of the prehospital infusion (i.e., during air medical transport) of AB plasma or low titer anti-B A plasma (2 units) in patients with hemorrhagic shock on clinical outcomes including 24 hour blood transfusion requirements, the development of multiple organ failure, nosocomial infection, acute lung injury (ALI) and transfusion related acute lung injury (TRALI). To determine the effect of prehospital infusion (i.e., during air medical transport) of AB plasma or low titer anti-B A plasma (2 Units) in patients with hemorrhagic shock on blood component transfusion and resuscitation requirements in the first 24 hours; on presenting coagulation parameters including INR, PT and thromboelastography measurements; and on IL-6 cytokine levels and markers of Protein C activation.
a. Secondary Aim #1: Determine whether prehospital infusion of AB plasma or low titer anti-B A plasma as compared to standard air medical care results in a lower 24 hour blood transfusion requirement, a lower incidence of multiple organ failure, nosocomial infection, acute lung injury and TRALI.
***Hypothesis Secondary Aim #1*:** Patients in hemorrhagic shock who receive AB plasma or low titer anti-B A plasma during air medical transport will have a lower 24 hour blood transfusion requirement, a lower incidence of multiple system organ failure, nosocomial infection, acute lung injury and TRALI as compared to patients who receive standard air medical care.

b. Secondary Aim #2: Determine whether prehospital infusion of AB plasma or low titer anti-B A plasma as compared to standard air medical care results in a reduction of blood component transfusion and resuscitation requirements over the first 24 hours post-injury.
***Hypothesis Secondary Aim #2*:** Patients in hemorrhagic shock who receive AB plasma or low titer anti-B A plasma during air medical transport will have a reduced fresh frozen plasma and platelet transfusion requirement, a reduced crystalloid and colloid requirement in the first 24 hours post-injury and will less commonly require vasopressor support in the first 24 hours post injury as compared to patients who receive standard air medical care.

c. Secondary Aim #3: Determine whether prehospital infusion of AB plasma or low titer anti-B A plasma as compared to standard air medical care results in improved coagulation measurements as determined by INR, PT, and thromboelastography parameters.
***Hypothesis Secondary Aim #3*:** Patients in hemorrhagic shock who receive AB plasma or low titer anti-B A plasma during air medical transport will have improved coagulation measurements as determined by INR, PT and thromboelastography parameters as compared to patients who receive standard air medical care.

d. Secondary Aim #4: Determine whether prehospital infusion of AB plasma or low titer anti-B A plasma as compared to standard air medical care results in lower levels of early IL-6 cytokine expression, reduced thrombomodulin and increased protein C levels.
***Hypothesis Secondary Aim #4*:** Patients in hemorrhagic shock who receive AB plasma or low titer anti-B A plasma during air medical transport will have reduced early IL-6 cytokine expression, reduced thrombomodulin and increased protein C levels as compared to patients who receive standard air medical care.

4. Project Milestones: Following final IRB approval for the University of Pittsburgh/UPMC and all participating centers, a 3 month start up period will be utilized to verify and educate all study sites prior to beginning

enrollment. Community notification and other procedures associated with the provisions for Exception from Informed Consent for Emergency Research will be initiated commensurate with IRB approval at all institutions. A data entry web based platform will be created. Enrollment will occur for 3.5 years with prospective data entry of laboratory and TEG measurements, clinical outcomes, transfusion requirements and demographic and injury characteristics. Serum for cytokine, protein C and thrombomodulin measurements will be batched and sent to the University of Pittsburgh on an annual basis. We expect approximately 25 patients per year per institution on average. Enrollment will be monitored on a semi-annual and annual basis for each participating center. Data safety and monitoring over the course of the clinical trial will fall under the responsibility of an independent data safety and monitoring board (DSMB). Interim analysis will occur when 1/3 and 2/3 of patients are enrolled. A 3 month data cleaning and wind down will occur once enrollment has been completed, allowing data analysis and manuscript preparation.

5. Military Significance/Public Purpose: Despite the significant advances in trauma care delivery and post-injury management practices which have occurred over the last decade, uncontrolled hemorrhage remains one of the leading causes of trauma related deaths.[4, 5, 81, 82]
To intervene early in the cascade of events which promotes and drives ongoing hemorrhage and the early coagulopathy that complicates injury has the potential to reduce overall transfusion requirements, alter the early systemic inflammatory response, and reduce the morbid clinical outcomes which are common in patients requiring large volume transfusion. It remains unknown the magnitude of effects associated with early intervention into this process. Importantly, the risks associated with early, prehospital administration of plasma remain unknown. In those patients who ultimately would not require large volume blood component transfusion, early plasma may be associated with a greater risk of acute lung injury or an exaggerated systemic inflammatory response in addition to any beneficial survival effect. The results and conclusions of the current proposal will allow and promote understanding of both the potential benefits and risks attributable to this type of intervention and this knowledge would have a direct impact on both military and civilian injured patients.

Due to the sparse nature of AB plasma processes and distribution procedures which efficiently allow and promote the availability of this product for prehospital providers has the potential to dramatically change the way our trauma systems are designed and currently function. The potential for additional blood transfusion components to be made available would open the possibilities of a flying or driving prehospital blood bank all with transfusion interventions in this setting. This potential knowledge base would be dramatically beneficial to both military and civilian trauma systems. It is in both these settings where the prehospital phase of treatment represents a relatively novel arena for new interventions.
It is anticipated that the results provided by the successful completion of this proposal will have paramount implications for both military and civilian injured patients as control of hemorrhage and delay to definitive care represent major impediments for both populations. This proposal will provide needed insight into the consequences of early plasma intervention in these critically injured patients when these impediments exist.

6. Patient Benefit: The potential benefit of 2 units of plasma in the prehospital period irrespective of transport time to the hospital is based upon the premise that intervening early in the viscous cycle of hemorrhagic shock and coagulopathy will be beneficial to patients. Plasma will provide needed coagulation factors to begin to interrupt the coagulopathy that is occurring which represents the mainstay of treatment in the hospital setting (once arriving at the hospital) Currently, standard of care at the vast majority of prehospital provider services is the use of crystalloid infusion. Longer transport times typically are associated with greater crystalloid volumes at our own centers. There are trauma centers and their respective prehospital services that have the ability to transfuse uncrossmatched packed red blood cells following persistent, unresponsive hypotension. **[83]** It is know that hypotension in the prehospital period is associated with a higher independent risk of mortality and worse outcome.[84-86] It is in these patients where the benefits of early prehospital plasma may be of greatest benefit. Improved outcomes have been demonstrated the earlier plasma is given after arrival to a trauma center.[87] **Recent, published evidence has documented the feasibility and potential benefits of a prehospital plasma resuscitation protocol similar to the current proposed trial. The small study demonstrates that patients who receive prehospital plasma, (as part of a plasma first resuscitation strategy) in patients with average transport times of 40 minutes, benefit by having an in hospital improved plasma: PRBC transfusion ratio throughout the first 24 hours, a reduction in crystalloid in**

<u>the prehospital setting, and early treatment of trauma-induced coagulopathy which is a known independent predictor of mortality.[83] (see Attached Manuscript)</u> This prior and recent evidence suggests the current trial has significant potential to provide benefit to patients.

<u>VII. Research Design and Methods</u>

<u>A. Study Design/Setting:</u> The study will be a 4 year, multi-center, open label, randomized trial utilizing level-1 trauma centers with busy air medical transport services where affiliations with local blood bank institutions exist. For patients with evidence of hemorrhagic shock being transported by air medical transport, the pre-hospital infusion of two Units of AB plasma or low titer anti-B A plasma will be compared to air medical standard of care. The University of Pittsburgh will serve as both the clinical outcome and data coordinating center for this multi-center clinical trial. Each individual institution will perform point of care rapid TEG analysis and coagulation measurements on site. UPMC Presbyterian is the busiest level-1 trauma center in the state of Pennsylvania and is affiliated with the largest non-profit air medical service in the country with an extensive track record of multi-center, in-hospital and prehospital clinical trials. All enrolling centers and respective investigators similarly have significant experience with multi-center trials and the research infrastructure to allow them to successfully participate in this research study. **It is anticipated that participating Institutions will include: University of Pittsburgh/UPMC, University of Texas Southwestern, University of Tennessee, Case Western Reserve University, University of Louisville and Vanderbilt University.**

<u>B. Study Population:</u> Blunt or penetrating injured patients with hemorrhagic shock being transported via air medical services from the scene of injury or from referring hospital to a definitive care trauma center participating in the trial.

<u>Inclusion Criteria:</u>

1. Blunt or penetrating injured patients being transported from scene or referral hospital to PAMPer site

<u>AND</u>

2. Systolic blood pressure below 90mmHg AND tachycardia>108 at scene, or at outside hospital or during transport.

<u>OR</u>

3. Systolic blood pressure below 70mmHg at scene, or outside hospital or during transport.

<u>Exclusion Criteria</u>:

1. Wearing NO PAMP opt –out bracelet
2. Age > 90 or < 18 years of age
3. Inability to obtain intravenous or interosseous access
4. Isolated fall from standing injury mechanism
5. Documented (radiographic evidence) cervical cord injury with motor deficit
6. Known prisoner or known pregnancy
7. Traumatic arrest with > 5 minutes of CPR without return of vital signs
8. brain matter exposed or penetrating brain injury (GSW)
9. Isolated drowning or hanging victims
10. Isolated burns > estimated 20% total body surface area
11. Referral Hospital In-patient admission
12. Objection to study voiced by subject or family member at scene

Inclusion and exclusion criteria will be assessed based on available information at the time of enrollment. Although all reasonable efforts will be made by the air medical crew to either directly witness or obtain documentation of qualifying vitals, due to the nature of the emergency pre-hospital setting, there may be occasions where the air medical crew must rely on verbal report of inclusion criteria, including qualifying vitals, from the referring hospital or ground crew. In these instances, if, after subsequent review of outside hospital

and/or ground crew documentation, it is determined that the subject did not meet inclusion criteria and/or met exclusion criteria, the subject will remain enrolled in the study based on the intention-to-treat principle.

In the event that a verbal report must be used in lieu of physical documentation or directly witnessing the qualifying vitals, documentation of the verbal report will serve as the source documentation for determining eligibility. Verbal reports will be documented in the air medical record and will detail the information reported and by whom.

C. Randomization: A single stage cluster randomization scheme will be utilized**.** Air medical services at each respective participating institution (2 to 6 bases or helicopters at each center) will be block randomized and assigned to the plasma arm or standard care (control) arm for 1 month at a time. The cluster design will be at the level of the helicopter. The block scheme will vary randomly between 2, 4, and 6 month block sizes over the period of enrollment for the trial at each participating center. Examples of a 4 month block randomized possibilities for a single helicopter or air base every month would be: PPSS, PSPS, PSSP, SPSP, SPPS, or SSPP (P=plasma, S=standard care.)

Randomization assignments for each center will be determined prior to the start of enrollment following the above single stage cluster design using standard computerized randomization software. Communication with the blood bank transportation services and each respective air base will occur and an annual schedule of randomization assignment will be distributed to all air base sites by the coordinating center. This specific randomization scheme is required due to the limited supply of AB universal donor plasma and also due to the logistics of the intervention. AB plasma or low titer anti-B A plasma is required to be delivered to and available at each institution's respective air base and on board the appropriate helicopter which is randomized to plasma intervention. Due to these factors, patients are not feasibly able to be randomized individually as this would require excessive amount of AB plasma or low titer anti-B A plasma to be on board at all air base or helicopter services. Similarly, due to plasma distribution requirements, monthly random assignments of air base of helicopter services to plasma or standard of care reduces logistical demands for blood bank services and prehospital providers. Importantly, prehospital care providers or in-hospital physician teams will not be formally blinded to whether a patient receives plasma or standard air medical care. However, we will attempt to minimize ED and staff treatment bias by utilizing sham plasma transfusion bags which will be brought in to the trauma bay by the air medical crew in those patients who meet inclusion criteria during randomized months where plasma is not given. Additionally, all steps will be undertaken so that data analysis will be performed in a blinded fashion. Sham bags will be distributed to all participating helicopters and air bases for the entire duration of the study and will be utilized in those months where plasma is not on board and in patients who meet inclusion criteria.

D. Intervention: 2 units of thawed AB plasma or low titer anti-B A plasma not older than 5 days will be infused in eligible patients who are randomized to the plasma arm of the clinical trial. The AB plasma or low titer anti-B A plasma will be that which is routinely used clinically; as collected, tested and stored by established (FDA-registered) blood collection and banking laboratories. Those air medical transport bases randomized to prehospital plasma infusion will be routinely (i.e., for the month of randomized assignment to the plasma arm) stocked with 2 Units of thawed AB plasma or low titer anti-B A plasma, which will be transported in a cooler with a temperature between 1 to 10 degrees centigrade and stored in a monitored refrigerator between 1 to 6 degrees centigrade. To minimize waste of AB plasma or low titer anti-B A plasma, local blood bank affiliates in coordination with each participating center will exchange unused plasma before the end of the fifth day thereby allowing subsequent clinical use by the respective blood bank facility. This will also be accomplished in a cooler with a temperature between 1 and 10 degrees Centigrade. The respective transfusion services at each participating center will have full and complete access to the storage records to verify that the plasma products have been properly stored prior to their exchange. A blood bank technician at each respective center will be in charge of monitoring all units of plasma at each air base, the age of each unit of plasma and the timing of transport services used to transport unused plasma back to the blood bank facility. Storage records at that time will verify appropriate storage and age of the plasma prior to placing it back in the blood bank plasma pool.

Those patients with persistent hypotension (SBP<90mmHg) after completion of the 2 units of plasma or low titer anti-B A plasma will follow a goal directed prehospital crystalloid resuscitation standard operation procedure which includes crystalloid bolus infusion or uncrossmatched blood depending on the particular air medical service for patients who remain hypotensive after the plasma intervention. Pre-hospital infusion of 2 units of AB plasma or low titer anti-B A plasma, once initiated, will be continued until completion thru trauma center arrival and ongoing resuscitation required in the trauma bay. Following completion of 2 units of plasma in the prehospital setting, air medical transport will continue standard air medical care and a goal directed prehospital resuscitation strategy. Upon trauma bay arrival, no further infusion of AB plasma or low titer anti-B A plasma will be initiated for the study. Upon trauma bay arrival, and once the plasma intervention (2 units AB plasma or low titer anti-B A plasma) is completed, ongoing trauma resuscitation will occur at the discretion of the trauma surgeon and emergency staff at the trauma center using In hospital standard operation procedures (**see section F2. below**) as guidelines. Resuscitation will be considered <u>in-hospital</u> rather than <u>pre-hospital</u> following trauma bay arrival or at the time of completion of the second unit of AB plasma or low titer anti-B A plasma infusion (Fig. 11 below).

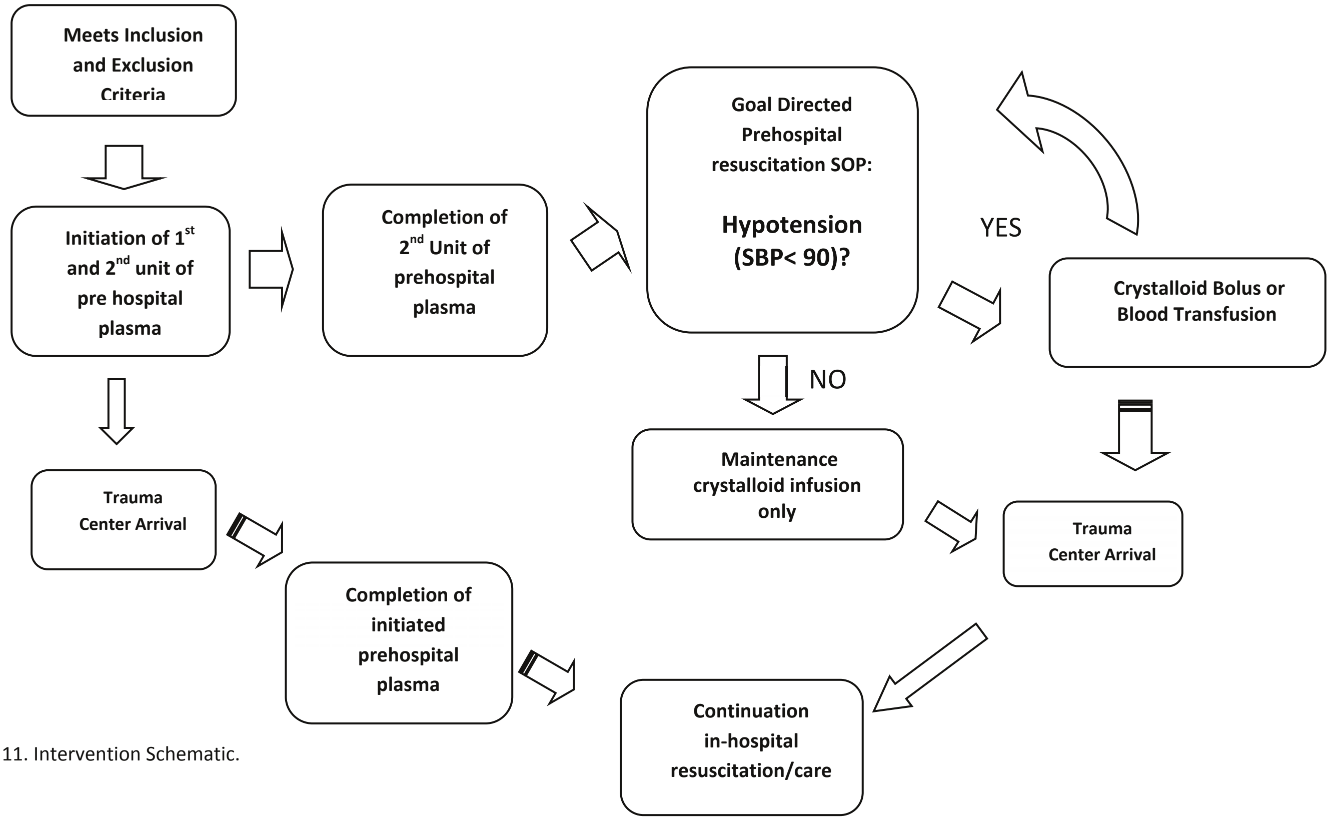

Figure 11. Intervention Schematic.

<u>E. Controls/Standard of Care:</u> On those air medical transport helicopters or air bases not randomized to have AB plasma or low titer anti-B A plasma for the month, patients who meet inclusion and exclusion criteria will undergo standard air medical care while following the prehospital resuscitation standard operating procedures (goal directed crystalloid resuscitation) as demonstrated in Figure 11.

<u>F. Standard Operating Procedures (SOPs):</u>

<u>1. Prehospital SOPs:</u> To minimize important differences for the early pre-hospital management of each patient, scene time, referral hospital time and definitive transport times for air medical services will be obtained, recorded and monitored including pre-hospital interventions. Those air medical scene times which exceed a standard deviation above the average time for each center will be flagged and investigated by the site PI and

overall study PI (Dr. Sperry). The individual pre-hospital times will be controlled for in our primary and secondary endpoint model analyses.

Evidence has accumulated regarding the potential negative effects of excessive crystalloid particularly in patients with hemorrhagic shock. A standard operating procedure (SOP) for crystalloid/resuscitation management for enrolled patients in the pre-hospital setting has been created to limit excessive crystalloid administration during the window of plasma intervention completion and trauma center arrival based upon hemodynamic status (SBP < 90mmHg) for a ‘goal directed resuscitation. (**Figure 11. above**) These crystalloid volumes will be monitored relative to transport time for all patients and across enrolling sites.
Some of the participating centers for the proposed study have the ability to carry uncrossmatched blood routinely on their air medical services to be initiated during flight. The air medical protocols for these institutions to transfuse blood en route occurs when greater than 2 liters of crystalloid/ resuscitation are infused with ongoing hypotension. The air medical services carry only 2 units of PRBCs (‘O’ negative) carried in approved coolers, with appropriate recordings to comply with blood banking standards, with similar look back procedures as is being proposed for the current trial for plasma. These protocols are already up and running at each respective institution and will not be altered by the current trial. This has become standard of care for these air medical services. For the proposed intervention to be most applicable across the majority of trauma air medical systems across the country, we will continue this practice. The inclusion criteria for the PAMPer trial will enroll patients based upon the proposed inclusion and exclusion criteria well before this blood transfusion threshold is met. **In those patients who remain hypotensive after the 2 units of AB plasma** or low titer anti-B A plasma**, the respective institutions will follow their own air medical transfusion guidelines (transfusion initiation if continued hypotension with concern for bleeding after 2L of resuscitation or following discussion with their respective medic command).** Any additional blood en route will occur following completion of the 2 units of AB plasma or low titer anti-B A plasma following each respective institutions current guidelines which are both following 2 liters of resuscitation with direction from their medic command center. **We will control and adjust for prehospital blood transfusion (# of units) in all of our primary and secondary analyses and will further characterize this prehospital variable in our predefined subgroup analysis.**

At the initial writing of the proposal, 2 month data were collected during the busy months for each of the centers looking at crystalloid and transport times from the projected air medical bases that could be used for the trial. Transport times vary with the distance the helicopter base is from the trauma center as flights are dispatched by their starting location and who is closest to the scene or referral hospital in most cases. There will be some variance depending on the air medical bases which are utilized at the formal initiation of the trial. Importantly, those patients with in hemorrhagic shock typically receive larger volumes of crystalloid volume. As only 20-30 patients are projected to be enrolled per year there were not enough that would meet inclusion criteria to include only likely eligible patients for the 3 month sampling. **We will control and adjust for individual prehospital crystalloid and transport times in all of our primary and secondary analyses and will further characterize these prehospital variables in our predefined subgroup analyses.**

Tabular Historic Data for transferred patients from potential air bases over a 2 month period:

	n	Crystalloid	Time (helicopter landing to trauma bay arrival)
Pittsburgh	42	235cc±321cc	40min±15min
Dallas	33	331cc±390cc	36min±19min
Louisville	40	290cc±400cc	35min±21min
Tennessee	29	352cc±290cc	34min±23min
Vanderbilt	35	270cc±231cc	36min±17min
Cleveland	31	257cc±302cc	39min±15min

2. In Hospital SOPs: We have selected level 1, academic, trauma centers with busy air medical transport services that are recognized for providing high level care of the injured patient. As the intervention is solely in the pre-hospital setting, there exists the potential for in-hospital management differences to occur across centers as in any multi-center study which does increase the study results applicability. However, to minimize

those differences where high level evidence exists for the early in-hospital management of each patient, and throughout a patients' admission, local SOPs for resuscitation and transfusion will be employed and monitored over the initial 24 hours and throughout a patients' admission. SOPs for patients who are at risk of massive transfusion (MT) will target an FFP: PRBC ratio of at least 1:2 based upon currently available data. Similar local SOPs for PLT transfusion ratios (1:2) will be employed and monitored during the initial 24 hours out from injury. Once 48 hours has passed without ongoing blood transfusion requirements, standard transfusion practice evidence in the ICU will be followed including standard restrictive transfusion guidelines for each respective institution in line with the TRICC trial recommendations (transfusion trigger of hgb- 7.0 in the ICU, non-bleeding patient).[88]

G. <u>Blinding:</u> Because of the pre-hospital setting of the intervention, the precious nature of AB plasma and all attempts to minimize waste of this resource, the transportation required to have thawed plasma at varied helicopter bases, and to minimize the air medical flight crews' requirements for the trial, the current randomization scheme at the level of the helicopter base was selected on a monthly basis and the intervention was unable to be blinded since transfusion of blood products requires documentation. However, we will attempt to minimize ED and staff treatment bias by adding sham plasma transfusion bags which will be brought in to the trauma bay by the air medical crew in those patients who meet inclusion criteria during randomized months where plasma is not given.

<u>H. Outcome Variables/Definitions:</u>

<u>1. Primary Outcomes:</u> Our primary outcome for the proposal will be 30 day mortality. It is this outcome variable for which the study will be powered.

<u>2. Secondary Outcomes:</u> Our secondary outcomes for the proposal will include clinical outcomes, 24 hour blood component transfusion and resuscitation requirements, coagulation parameters, and cytokine and protein C pathway measurements.

<u>3. Clinical Outcomes:</u> All clinical outcomes will be prospectively evaluated throughout ICU and hospital admission, and the timing from the day of initial injury will be recorded for time-to-event statistical analysis.

<u>a. Twenty Four-Hour Blood Transfusion Requirements:</u> 24-hour blood transfusion requirements will be determined by recording blood and number of Units transfused from the time of trauma bay arrival or upon completion of pre-hospital initiated plasma infusion. For survival bias analysis, number of blood transfusion Units received at 3, 6, 12, and 18 hours will also be recorded. Any initiation of blood transfusion will be considered completed.

<u>b. In-hospital Mortality:</u> In hospital mortality will be prospectively recorded from the time of trauma bay arrival. Over the first 24 hours we will document and record the time of death in hours, while after the 24 hour time point, we will document and record the time of death in days from arrival. We suspect that patients in hemorrhagic shock will have a significant percentage of mortality occurring in the first 24 hour period.

<u>c. Multiple Organ Failure:</u> Organ dysfunction will be evaluated via a well-validated scoring system referred to as the Denver Postinjury Multiple Organ Failure Score. Patients who are never admitted to the ICU or those with a length of ICU stay of less than 48 hours will be considered to have a Denver Score of 0. The Denver score rates the dysfunction of four organ systems (pulmonary, renal, hepatic, and cardiac), which are evaluated daily throughout the patient's intensive care unit stay and graded on a scale from 0 to 3 with the total score ranging from 0-12. Daily WORST laboratorial and physiologic values are used for the score. MOF scores are calculated as the sum of the simultaneously obtained individual organ scores on each hospital day and MOF status is defined as a score >3 occurring any day after 48 hours postinjury.

A determined from the day of initial injury for time-to-event analysis and multivariate Cox proportional hazard regression analysis.

Dysfunction	Grade 0	Grade 1	Grade 2	Grade 3
Pulmonary<br>PaO2/FiO2 ratio	> 208	208 - 165	165 - 83	< 83
Renal<br>Creatinine (umol/L)	<159	160 - 210	211 - 420	> 420
Hepatic<br>Total Bilirubin (umol/L)	< 34	34 – 68	69 - 137	> 137
Cardiac<br>Inotropes	No inotropes	Only one inotrope at a small dose *	Any inotrope at moderate dose or >1 agent, all at small doses *	Any inotrope at large dose or > 2 agents at moderate doses *

- Inotrope doses (in ug/ Kg / min):

	Small	Moderate	Large
Milrinone	<0.3	0.4 -0.7	>0.7
Vasopressin	<0.03	0.03 -0.07	>0.07
Dopamine	<6	6 - 10	>10
Dobutamine	<6	6 - 10	>10
Epinephrine	<0.06	0.06 -0.15	>0.15
Norepinephrine	<0.11	0.11 -0.5	>05
Phenylephrine	<0.6	0.6 - 3	>3

d. Nosocomial Infection: Infectious outcomes of interest will include ventilator associated pneumonia, blood stream infection and urinary tract infections. Surgical site infections and post-operative intra-abdominal collections will also be recorded but excluded as a principal secondary outcome event so as to reduce the confounding effects of operative interventions which not all patients require. The development of these nosocomial infections will be based upon positive culture evidence during hospital admission. Infections will be monitored until post-injury day 28 or ICU discharge. Diagnosis of a ventilator associated pneumonia requires a quantitative culture threshold of ≥ 104 CFU/ml from broncho-alveolar lavage specimens in addition to standard x-ray and clinical criteria. Diagnosis of catheter-related blood stream infections requires positive peripheral cultures with an identical organism obtained from either a positive semi-quantitative culture (>15 CFU/segment), or positive quantitative culture (>103 CFU/segment) from a catheter segment specimen. Urinary tract infections require > 105 organisms/ml of urine. All time variables to the respective outcome event will be determined from the day of initial injury, while the time to the first nosocomial infection will be used in those patients with multiple infections for time-to-event analysis and multivariate Cox proportional hazard regression analysis.

e. Acute Lung Injury (ALI) and Transfusion Related Acute Lung Injury (TRALI): Development of ALI will be assessed utilizing the 1992 American-European Consensus Conference definition [92] which includes: 1) bilateral infiltrates on chest x-ray, 2) a capillary wedge pressure < 18mmHg, and 3) Pao2/Fio2 ratio < 300 via blood gas analysis. In those patients without a Swan-Ganz catheter to determine capillary wedge pressure, the absence of signs of, or clinical concern, for elevated left sided atrial pressures will be used for the diagnosis. All patients who remain intubated beyond the first 24 hours post-injury will be evaluated using blood gas analysis and chest x-ray evaluation. Those patients who remain intubated at 48 hours through 7 days will be reevaluated for this outcome at these time points. All time variables to the respective outcome event will be determined from the day of initial injury for time-to-event analysis and multivariate Cox proportional hazard regression analysis. The diagnosis of **TRALI** will be defined as when ALI occurs within the first 6 hours from arrival at the trauma center as it is clinically defined.

Toy et al. reported a TRALI risk of 1:12,731 across all blood components including plasma. The most recent FDA mortality data for 2011 reports 4 TRALI related deaths due to plasma. These data suggest that the overall risk of TRALI is low. There are no definitive data on the risk of TRALI from an AB plasma or low titer anti-B A plasma unit from a multiparous female. The specific unit of thawed plasma which a patient would receive in the pre-hospital setting as the intervention would be the potential same thawed unit at the definitive trauma center and thus would inherently contain the same risks of complications as those transfused in the hospital. The risk of complications including TRALI are required to be specifically monitored for, recorded and investigated by every transfusion service at each enrolling center. Any transfusion complication in the pre-hospital setting will be similarly monitored. As the intervention is specific to the pre-hospital setting and since transfusion complications are temporally related to the specific transfusion, all transfusion related complications will be assessed during the initial 24 hours from arrival and recorded. All participating blood centers will minimize multiparous female donor plasma as is current standard of practice for blood banks.

f. Blood component transfusion and resuscitation requirements: 24-hour blood component transfusion requirements for fresh frozen plasma and platelet transfusion will be determined by recording blood component number of Units transfused for fresh frozen plasma and platelets from the time of trauma bay arrival or upon completion of prehospital initiated plasma infusion. Similar determinations for crystalloid requirements, colloid requirements (albumin, hetastarch) and whether the patient requires vasopressors (yes/no; norepinephrine, epinephrine, vasopressin or phenylephrine) in the first 24 hours post-injury will occur. For survival bias analysis, number of Units will also be determined at 3, 6, 12, and 18 hours for each transfusion and resuscitation component.

g. Coagulation Parameters: During the first 60 minutes (+ 12 hours) of the initial in-hospital resuscitation in the trauma bay or in the operating room (for those patients taken directly to the OR), but after transfusion of thawed plasma if hanging, blood for PT, INR and point of care rapid-TEG analysis will be obtained. These measurements will be repeated as close to 24 hours (+/- 12 hours) from the time of injury as feasible, to coordinate with other lab draws and staffing patterns. We will also be performing additional coagulation biomarker assays at these time points. Tissue factor will be added to a citrated whole blood collection tube and rapid TEG parameters including activated clotting time (ACT, seconds), angle (α, degrees), coagulation time (K, seconds), maximum amplitude (MA, mm), clot strength (G, dynes/cm^2), and estimated percent lysis (EPL, %) will be measured for each patient .

h. Cytokine and Protein C pathway measurements: During the first 60 minutes (+ 12 hours) of the initial in-hospital resuscitation in the trauma bay or in the operating room (for those patients taken directly to the OR), but after transfusion of thawed plasma if hanging, blood for IL-6 cytokine levels, thrombomodulin and protein C levels will be drawn along with blood for coagulation analysis. These measurements will be repeated at 24 hours (+/- 12 hours) from the time of injury as feasible, to coordinate with other lab draws and staffing patterns. An additional blood draw will be performed at 72 hours (+/- 12) for additional coagulation biomarkers. IL-6 levels rather than a large panel of early inflammatory cytokines will be measured; as IL-6 is one of the few cytokine markers shown to be associated with the development of multiple organ failure post injury.[93, 94] Altered thrombomodulin and protein C levels have similarly been shown recently to be associated with increased mortality, blood transfusion requirements, acute renal injury and greater ventilator requirements post-injury.[20] These outcome markers will provide information and insight into the mechanisms responsible for any beneficial effects of addressing hemorrhagic shock early in the prehospital setting. IL-6 will be measured using an ELISA immunoassay kit (Human IL-6 ELISA Kit, Antigenix America Inc., USA). Thrombomodulin and protein C levels will also be determined utilizing ELIZA immunoassay techniques (Asserachrom Thrombomodulin EIA, Diagnostica Stago, USA) and (Staclot Protein C clot-based activity assay, Diagnositca Stago, USA), respectively. All cytokine and Protein C pathway samples will be stored and batched at their respective institution and delivered to the University of Pittsburgh Coordinating Center where formal ELISA immunoassay measurements will be undertaken.

VIII. Human Subjects

We anticipate that this study will be conducted under the federal provisions governing Exception from the Requirement for Informed Consent for Emergency Research, including community consultation, public

notification, as well as notification of patients or their legally-authorized representative as soon as feasible after enrollment. The latter shall include provision of an opportunity to opt out from ongoing participation that will be given through oral and written communication.
Community consultation as determined by the local IRB will be undertaken prior to final IRB approval. Since the population eligible for enrollment includes all citizens in the study regions it will not be possible to target any particular small group. Feedback from the community will be obtained by research personnel regarding any concerns they may have about potential enrollment. If requested, bracelets will be made available that could be worn by members of the community who do not want to participate. Public notification and community consultation will be performed as directed by the local IRB and may include such methods as using random digit dialing telephone surveys of the proposed study community, targeted small group meetings or consultation with community leaders. Due to ongoing participation in numerous multicenter research studies involving emergency research, our institution and the other participating institutions have significant experience with community consultation and notification practices. The requirements for Exception from the Requirement for Informed Consent for Emergency Research and our proposal's characteristics regarding these can be found in the **Clinical Protocol Appendix 1**. Once a participating institution completes the processes for obtaining IRB approval of the research protocol, the IND will be amended to include the institution as a study site. The respective information submitted to the IND application will include a summary of the community consultation process accepted by the reviewing IRB. **Community Consultation Plan (appendix 2**) attached.

Benefits of participation in the PAMPer trial for both plasma and standard of care subjects:

A unique benefit regarding participation in the PAMPer trial is that all research results for both plasma and control arms of the study may be used to further inform clinical care decisions throughout a participants hospital stay. We have recently presented research currently *in press* (Brown et al. Journal of Trauma and Acute Care Surgery, 2013) documenting the potential benefits of goal directed prehospital crystalloid resuscitation. Based upon this knowledge for all trial participants, a prehospital standard operating procedure (SOP) utilizing a goal directed crystalloid resuscitation guideline will be followed. Due to the variability of prehospital crystalloid resuscitation that currently exists across injured patients, this will potentially benefit participants in either arm of the study.

Participation in the trial may also aid in early recognition of trauma induced coagulopathy due to the early measurements of INR and thrombelastography (TEG) which will be performed on all enrolled subjects. TEG is an FDA approved tool, however, currently it is not standard of care and only a small proportion of trauma centers across the country routinely obtain early INR and point of care rapid-TEG analysis in the emergency department, soon after arrival in patients in hemorrhagic shock. Early recognition of coagulopathy for all enrolled subjects may lead to earlier intervention and in hospital mechanisms that improve clinical outcome.

For all participants in the trial, early and continual screening and assessment for clinical outcomes including multiple organ failure (MOF), nosocomial infection (NI) and acute respiratory distress syndrome (ARDS) will occur and will have the potential to again benefit all participants, irrespective of which arm of the study they are randomized to, as early surveillance for these clinical outcome may lead to beneficial effects. It is the layering of standardization of prehospital resuscitation, early diagnosis of trauma induced coagulopathy and additional early assessment and screening for important clinical outcomes including MOF, NI, and ARDS that highlights the benefits of participation in the PAMPer trial for both plasma and control arms of the study.

<u>1. Screening and Enrollment:</u> Subjects will be identified prospectively by air medical transport personnel familiar with the inclusion and exclusion criteria. The intervention takes place on board the air medical transport vehicle by personnel trained in blood product administration. Site research coordinators will document and verify all trauma arrivals via air medical transport for enrollment*.* Those patients who met inclusion and no exclusion criteria will have been assigned to AB plasma / low titer anti-B A plasma or standard of care (i.e., based on the study site randomization code). Subjects not enrolled by prehospital personnel may be identified and enrolled by research personnel as control subjects upon arrival to the ED. Once in the emergency department, the subjects will undergo initial blood sampling for our secondary outcomes of interest, and will have point of care rapid thromboelastography (TEG) performed for coagulation parameter measurements within 60 minutes of patient arrival and again the next day.

2. Informed Consent and Notification: If any subject or family member voice objections to being included in research at the scene, the subject will not be included. Once subjects have arrived at the hospital, they will be approached for informed consent as soon as possible, or their legally authorized representative, if available, will be approached if subject is unable to consent. We expect most of these subjects to be unable to prospectively provide consent due to the critical nature of their injuries. We also anticipate that, in many cases, the subject's legally authorized representative will not be readily available at the injury scene to prospectively provide informed consent. The subject's capacity to consent will be determined by the treating physician at the hospital. All consenting and notification will be accomplished by research team members trained in informed consent processes, HIPAA laws, and the protocol. For those subjects that expire due to their injuries, next of kin will be notified of their involvement in the trial.
In our experience, there is no single time-line that is appropriate for all subjects or families who are dealing with the end-of-life or social issues surrounding resuscitation from traumatic injury. The treating healthcare team can guide appropriate timing of discussions about our research. When a family member refuses to provide consent for the subject's participation in the study, they will be provided with a notification form approved by the IRB. Additionally, for subjects that do not survive we will send a certified letter, also approved by the IRB.

We will keep a log that reflects the required steps for contacting the LAR or family member. The checklist will be completed for each subject enrolled and included in the subject's research records. **See Appendix 3**
We will attempt to notify the family as soon as possible in person. In the event that they cannot be contacted in person (for example, if they are outside of the state), we will notify them by registered mail. Subjects (or their legally authorized representatives) may refuse follow-up and/or access to medical record review as stated in the notification form. This will be documented in the subject's case history, along with the date and reason for withdrawal. The study investigators may examine data that have already been collected in order to determine safety. Subjects who wish to withdraw also will be reminded that total withdrawal will prevent the investigators from identifying any potential adverse events. Outcomes for subjects who withdraw or who decline consent to the follow-up portion of the study will be assessed by use of existing public databases such as obituaries or the Social Security Death Index (SSDI).

The original informed consents will be kept in a binder in a locked secure cabinet at each study site. Copies of all notifications will also be kept in a binder in a locked cabinet. These documents will be kept for a minimum of 7 years after study analysis is completed. Then they will be destroyed.

3. HIPAA: language is included in our informed consent which contains adequate written assurances that protected health information (PHI) will not be disclosed to any person or entity other than those listed on the informed consent. This research could not practically be conducted without access and use of PHI for safety reasons.

IX. Sample Storage

Detailed instructions beyond what is presented here regarding blood sample collection, processing, storage, packaging, and shipping will be provided in a separate manual of procedures. Samples will be kept for at least 7 years.

Future uses of blood samples obtained in this study will not include DNA or genetic testing. Future testing by members of the PAMPer team may include additional physiological markers that may indicate mechanisms of early coagulopathy of trauma that have not yet been identified.

Upon receiving all specimens, research lab personnel will inspect the sample integrity and document the conditions (for example: thawed, vial broken, clotted, etc.). The plasma samples obtained during PAMPer will be maintained by the University of Pittsburgh Coordinating Center in a -80°C freezer.

X. Data

1. Sources: Data will be collected prospectively as patient care progresses. This will include a review of the air medical patient care report(s), Emergency Department and electronic/ paper hospital records.

<u>2. Prehospital Resuscitation Elements:</u> Demographics, air medical response times, injury characteristics, vital signs, prehospital resuscitation characteristics, (plasma volume, crystalloid volume, blood transfusion volume, starting at referring hospital or scene) prehospital interventions (needle decompression, chest tubes) referring hospital vitals, and interventions.

<u>3. In-Hospital Resuscitation Elements:</u> Demographics, shock severity (base deficit, lactate), injury characteristics, ED vitals, ED interventions (chest tubes, intubation), injury severity, operative interventions and timing of interventions, injury severity score, ICU days, ventilator days, length of stay, multiple organ dysfunction scores (daily), nosocomial infectious outcomes, blood gas results, chest x-ray reads, transfusion of blood and blood components, resuscitation requirements.

<u>4. Data Entry:</u> MACRO and associated internet technology affiliates at the University of Pittsburgh will create web-based HTML forms to collect necessary information from all participating sites. Web entry forms will have dynamic features such as immediate checks on data and relationships within a form and between forms. Details and clarification about data items will be provided using pop-up windows and links to appropriate sections of the on-line version of the Manual of Operations. Data encryption and authentication methods will be used. Additional features will be built into the web entry forms including: forms transmission history, access to past forms, tracking of data corrections, and the capability to save and re-load incomplete forms. The subjects will be identified by a study number only. All clinical interventions will become part of the patient's medical records including plasma transfusion. All hard copy source documentation will be kept in a secured, locked cabinet in the site's research coordinator's office. All study documents will be maintained in a secure location for the time frame designated by each participating site's requirements. The electronic data will be entered and maintained on a password protected SSL website designed for this trial.

The data entered for the PAMPer trial will be maintained at the University of Pittsburgh on a relational database. The database would be housed in a virtual environment so in the event of a hardware failure it would migrate to a new host. The data will be backed up 4 times a day with full transaction log files in use and copies of the data will be stored offsite with a secure service, Iron Mountain. In addition to the data server, the production web server will also be backed up routinely and as a virtual machine can be transitioned to different hardware automatically in case of hardware failure. All Servers are behind an enterprise firewall and access has to be granted through the firewall even within the University Network. Research laboratory results will also be downloaded to the study designated program.

<u>5. Database Management:</u> A two-tiered database structure will be created. A front-end database will serve the web entry needs, using a database management system well-suited to handling updates from multiple interactive users. The data from this database will be transferred periodically (e.g. weekly) to a data repository that can be used by statistical software packages. These data sets will be the basis for data queries, analyses and monitoring reports. Various versions of this database will be kept as needed, e.g. for quarterly performance reports. Backup of data and programs will be performed at frequent intervals. Access to data will be limited to those who need access to perform their tasks. The database management system is able to manage large quantities of data, to merge data from multiple databases as required, to handle complex and possibly changing relationships, and to produce analysis datasets that can be imported into a variety of statistical analysis packages.

<u>XI. Analysis Plan</u>

The over arching goal of the study proposal is to assess the efficacy and safety of prehospital plasma infusion as compared to standard of care for injured patients in hemorrhagic shock who require air medical transport. All primary and secondary analyses will be performed based on the Intent-to-Treat principle and will include all enrolled patients grouped by randomization.

<u>1. Data Analysis For Primary Hypothesis:</u>

a. Hypothesis Primary Aim #1: Using the hypothesis patients in hemorrhagic shock who receive AB plasma or low titer anti-B A plasma during air medical transport will have a reduced 30 day mortality as compared to patients who receive standard air medical care.

In this phase III clinical trial our primary outcome of 30-days mortality will be analyzed on the basis of intent-to-treat. All subjects within all 32 clusters randomized will be included for the primary clinical outcome analysis. 30 day mortality will be computed based on our data collection and follow up using all available sources including social security death records. Subjects who have not been reported as deceased by day 30 following ED admission from any of the sources used for query, multiple imputation will be used under the assumption that the missing data are not missing at random. Further details concerning the determination a subject death is included in the data collection section of this protocol. If more than 15% of the subjects are missing the 30 days mortality data, our primary outcome will be analyzed descriptively without inferential testing. The proportion of mortality within each cluster (level of randomization) will be computed and pooled for both intervention and control groups. For our cluster design, the primary clinical outcome of 30-day mortality will be assessed as a fixed end point of time using test of proportions differences, pooled z-test with continuity correction applied. Our statistical testing is part of 3 sequential tests that determined using the O'Brien-Fleming spending function to determine the test boundaries at each look including the final analysis as described in our power analysis.[95] P value and related effect size with 95% confident interval for 30 days mortality differences will be computed. Cluster size variation will be checked and consequently a weighted analysis to account for related variation will be performed. We will take advantage of the advanced method in accounting for intra-cluster correlation in which will help in increasing the statistical power of our analysis. Accounting for cluster effect can be accomplished by dividing z-value on the square root of the design effect in this study.[96] The revised z-value adjusting for clustering is calculated with following equation:

$$\frac{z - Value}{\sqrt{(design\ effect)}}$$

As an extension for the above analysis, multiple regression including generalized estimating equations (GEE) will be used as well to adjust for cluster level covariates and to incorporate patient level covariates through a two-stage process.[97] This approach of modeling techniques allows the inherent correlation within clusters to be modeled explicitly and a more satisfactory model can be obtained.[98, 99] For this regard in utilizing a statistical modeling we will be able to identify the main factors that explain variation in the outcome. Our analysis plan is to adjust for the effect of related covariates before testing the effect of our intervention (pre-hospital plasma) rather than to maximize the proportion of variation explained. Ensuring that our modeling is hypothesis-led rather than data driven, we have considered the most covariates which are to be included in our model with the intervention variable fitted last (pre-hospital plasma). Of these covariates: prehospital transporting time, pre-hospital blood transfusion and crystalloid, mechanism of injury, head injury/GCS, age, and gender. The priority will be for base line covariates which reveal imbalance in between clusters or treatment groups. Site as a stratifying variable will be included as a random effect .[100] The number of variables that we may adjust our main effect for will be according to related relevancy and sample size adequacy. We are also aware that we should avoid the extended enthusiasm in over adjusting to avoid diluting the intervention main effect of our trial. Sensitivity analysis of 30 days mortality will be performed to check the effect of imputation as alive on the treatment group for comparisons and related confident intervals. Additional statistical technique may be added based on DSMB recommendations. STATA software, StataCorp, College Station, TX, will be used in our analysis. Interim and final analyses of the primary outcome will be the same, and both adjusted and unadjusted p-values will be presented for all analyses.

2. Secondary Hypotheses:

a. Hypothesis Secondary Aim #1: Using the hypothesis patients in hemorrhagic shock who receive prehospital AB plasma or low titer anti-B A plasma during air medical transport (Ppp) will have a lower 24hour blood transfusion requirement, a lower incidence of multiple system organ failure, nosocomial infection, acute lung injury and TRALI as compared to patients who receive standard air medical care (Psc), we will test the

null hypothesis of Ppp=Psc versus the alternative hypothesis that Ppp<Psc. In testing the significance for our secondary aims, data will be checked for equal variance across clusters and adjustment for fixed effects and random effects will be incorporated in any modeling when needed. Assuming equal variance, we will utilize the two-sided Mann-Whitney U or Fishers Exact test for these secondary clinical outcome comparisons. Time-to-event analysis using Kaplan-Meier and log rank comparison will also be performed utilizing the timing of the secondary outcome event in days and censoring patients who suffer mortality prior to any outcome event. Regression analysis of individual level data using methods for clustered data (adjusting the standard errors for the design effect) will be used in order to analyze the effect of intervention on the outcome variables adjusting for all confounding, covariates and expected interactions. Models will be compared with the likelihood ratio test.

b. Hypothesis Secondary Aim #2: Using the hypothesis patients in hemorrhagic shock who receive prehospital AB plasma or low titer anti-B A plasma during air medical transport (Ppp) will have a reduced fresh frozen plasma and platelet transfusion requirement, a reduced crystalloid and colloid requirement in the first 24 hours post-injury and will less commonly require vasopressor support in the first 24 hours post injury as compared to patients who receive standard air medical care (Psc), we will test the null hypothesis of Ppp=Psc versus the alternative hypothesis that Ppp<Psc. In testing the significance for our secondary aims, data will be checked for equal variance across clusters and adjustment for fixed effects and random effects will be incorporated in any modeling when needed. Assuming equal variance, we will utilize an independent samples Mann-Whitney U test for these secondary hypothesis comparisons of resuscitation volumes across randomization groups as they will not be normally distributed. We will utilize the two-sided Fisher Exact test for vasopressor requirement (yes/no) in the intial 24 hours post-injury across randomization groups. Similar analyses controlling for possible survival bias will be performed as proposed for our primary outcome, 24 hour blood transfusion requirements.

c. Hypothesis Secondary Aim #3: Using the hypothesis patients in hemorrhagic shock who receive prehospital AB plasma or low titer anti-B A plasma during air medical transport (Ppp) will have improved coagulation measurements as determined by INR, PT and thromboelastography parameters as compared to patients who receive standard air medical care (Psc), we will test the null hypothesis of Pp=Psc versus the alternative hypothesis that Ppp<Psc for each coagulation parameter. In testing the significance for these secondary aims, data will be checked for equal variance across clusters and adjustment for fixed effects and random effects will be incorporated in any modeling when needed. Assuming equal variance, we will utilize an independent samples Mann-Whitney U test for these secondary hypothesis comparisons of coagulation parameters as they will not be normally distributed. We will additionally control for mulitple comparisons. Similiar analyses for the two time points will be performed (first 60 minutes (+12 hours) and 24 hours).

d. Hypothesis Secondary Aim #4: Using the hypothesis patients in hemorrhagic shock who receive prehospital AB plasma or low titer anti-B A plasma during air medical transport (Ppp) will have reduced early IL-6 cytokine expression, reduced thrombomodulin and increased protein C levels as compared to patients who receive standard air medical care (Psc), we will test the null hypothesis of Ppp=Psc versus alternative hypotheses (Ppp<Psc for IL-6 and thrombomodulin and Ppp>Psc for protein C levels). In testing the significance for these secondary aims, data will be cheeked for equal variance across clusters and adjustment for fixed effects and random effects will be incorporated in any modeling when needed. Assuming equal variance, we will utilize an independent samples Mann-Whitney U test for these secondary hypothesis comparisons of cytokine and protein C pathway moeities as they will not be normally distributed. Similiar analyses for the two time points will be performed (first 60 minutes (+12 hours) and 24 hours).

3. Predefined Subgroup Analyses: Predefined subset analyses will be performed looking at 1a.) patients who ultimately did or did not required massive transfusion in the first 24 hours (≥ 10 Units PRBCs) 1b.) patients who ultimately required ≥ than 4 Units of PRBC's 2.) those patients who received or did not receive prehospital PRBC transfusion, 3.) those patients with significant traumatic brain injury (Head AIS >2) versus those without significant brain injury (Head AIS ≤ 2), 4.) those patients enrolled from the scene of injury versus those enrolled from a referral hospital, 5.) those patients with a preinjury history of vitamin K antagonist medication versus those without, 6.) those patients with preinjury history of antiplatelet medication, 7.) those patients who suffered blunt injury as compared to those who suffered penetrating injury, and 8.) those patients with high versus low field to ED transport times (median split subgroups) . It is recognized that the study is not

appropriately powered for these subgroup comparisons and the results and conclusions formulated from these subgroup analyses will be considered exploratory in nature and will not be used as a basis for treatment recommendations.

4. Randomization of Ineligible Subjects: It is anticipated that there will be a small proportion of patients enrolled who receive either AB plasma/low titer anti-B A plasma or standard of care that in retrospect will not have met the entry criteria and are thus ineligible. In this circumstance, patients will be analyzed according to the group to which they were randomized. Subgroup analyses based on eligibility criteria will be performed if the number of patients so affected is large. However, based on the relatively limited inclusion and exclusion criteria it is anticipated that the frequency of this event will be low.

5. Non-adherence: In some circumstances, patients may receive standard care instead of AB plasma/low titer anti-B A plasma intervention when randomized to AB plasma /low titer anti-B A plasma for that month. Non-adherence is most likely to occur in the case of the exsanguinating patient when AB plasma or low titer anti-B A plasma despite being available is not used. Fortunately, this event is relatively rare. In keeping with the intention-to-treat analytic design, these patients will be analyzed with the group to which they were randomized.

6. Sample Size Justification and Power Analysis: We have determined the sample size for this proposal and powered the analysis based upon our primary outcome (30 day mortality) as this is a traditional trauma trial standard for evaluating delayed complications and safety of trial interventions, the benefit is durable, the outcome is important to scientists and patients and provides evidence to support the most efficient use of the nation's blood supply. All subjects will be tracked for vital statistics for a full 30 days, whether or not they have left the hospital.

7. Blood Transfusion at 24 hours Secondary Outcome: Baseline references for the average 24 hour blood transfusion requirement in injured patients with hemorrhagic shock vary in the literature and depends on multiple factors including shock severity, injury severity, age, mechanism of injury and transfusion practice at the institutions being analyzed.[36, 45, 47, 68, 101-105] From these baseline references we determined that the average requirement for blood in initial 24 hours for patients in hemorrhagic shock is 15.0±12 Units. Based upon the sample size estimate for our primary outcome of 30 day mortality, we will have 80% power to detect at least a 20% reduction in 24 hour blood transfusion requirements.

8. Sample Size Calculation for a Cluster Design: To appropriately power the study for 30 day mortality, we have utilized, as of yet, unpublished prospective data from the *Inflammation and the Host Response to Injury Large Scale Collaborative Program, (www.gluegrant.org)* supported by the National Institute of General Medical Sciences (NIGMS) or more commonly termed 'Glue Grant' study and additional published literature to estimate our baseline mortality and effect size for the study. In hemorrhagic shock patients enrolled in the Glue Grant, patients who require at least 3-4 units of blood within the first 6 hours of injury had a in hospital 21.3% to 22.4% mortality, respectively. This is similar and in conjunction with prior published literature in hemorrhagic shock patients.[51, 105-108] Based upon these point estimates we will use a baseline mortality of 22% for our power calculations. By intervening early into the coagulopathy which complicates significant traumatic injury and hemorrhagic shock, the intent of the trial would be to improve outcomes (30 mortality) by reducing transfusion requirements, reducing the need for massive transfusion (> 10 units of blood in 24 hours post injury) and reducing the inflammatory response which blood transfusion has been shown to be an independent risk factor for. Again, using the Glue Grant dataset, for those patients who required between at least10 units and 15 units of blood transfusion over the initial 24 hours following injury, the mortality rate was 7.6% and 8.3%, respectively.

For our sample size estimation for the 30 day mortality outcome, we chose a difference of 14% (22% to 8%, see Glue Grant point estimates above) from a baseline mortality of 22% when comparing patients randomized to plasma versus standard of care. The trial will be powered at 88% with a two-sided alpha level of 0.05, adjusted for interim analyses to 0.037 (interim analyses at 1/3 and 2/3 enrollment). The additional power will allow adjustment of potential unequal cluster sizes as this could decrease the statistical power of the study. We considered a between group difference in 30 day mortality of 14% or greater to be clinically meaningful and of

sufficient magnitude to influence clinical practice. Adjusting for site generally should increase power unless there is a lack of homogeneity of treatment effects across sites.

The required sample size is 144 patients in each group multiplied by **1.75** to adjust for cluster design (average 16 patients per cluster) and addition of 12% for missing data giving a needed number of patients of 282 for each group and **564 patients total**. We will have 80% power to detect a 13% difference from baseline and 75% power to detect a 12% difference. Our sample size will provide additional power to overcome the possibility of within cluster variation due to cluster design.

**Power**	**N1**	**N2**	**Alpha**	**Beta**	**P1**	**P2**
0.883804	144	144	0.050000	0.116196	0.22	0.08
0.874007	140	140	0.050000	0.125993	0.22	0.08
0.863480	136	136	0.050000	0.136520	0.22	0.08
0.855080	133	133	0.050000	0.144920	0.22	0.08

**Assumption**	**Control group**	**Intervention group**	**Absolute differences (effect size)**	**Estimated Power***
**1**	**22%**	**10%**	**12%**	**75%**
**2**	**22%**	**9%**	**13%**	**80%**
**3**	**22%**	**8%**	**14%**	**88.4%**

Assuming 0.038 alpha level, two sided, test of proportions differences, z-test with continuity correction applied.
(Power analysis performed using PASS statistical software, Number Cruncher Statistical System, Kaysville, Utah).

The above power analysis generated assuming 3 sequential tests are made using the O'Brien-Fleming spending function to determine the test boundaries and depicted in the following table and graph:

Details when Spending = O'Brien-Fleming, N1 = 144, N2 =144, P1 = 0.22, P2 = 0.08, Continuity Correction.

**Look**	**Time**	**Lower Bndry**	**Upper Bndry**	**Nominal Alpha**	**Inc Alpha**	**Total Alpha**	**Inc Power**	**Total Power**
1	0.33	-3.71030	3.71030	0.000207	0.000207	0.000207	0.030172	0.030172
2	0.67	-2.51142	2.51142	0.012025	0.011890	0.012097	0.501816	0.531988
3	1.00	-1.99302	1.99302	0.046259	0.037903	0.050000	0.351816	0.883804
Drift	3.17317							

O'Brien-Fleming Boundaries with Alpha = 0.05

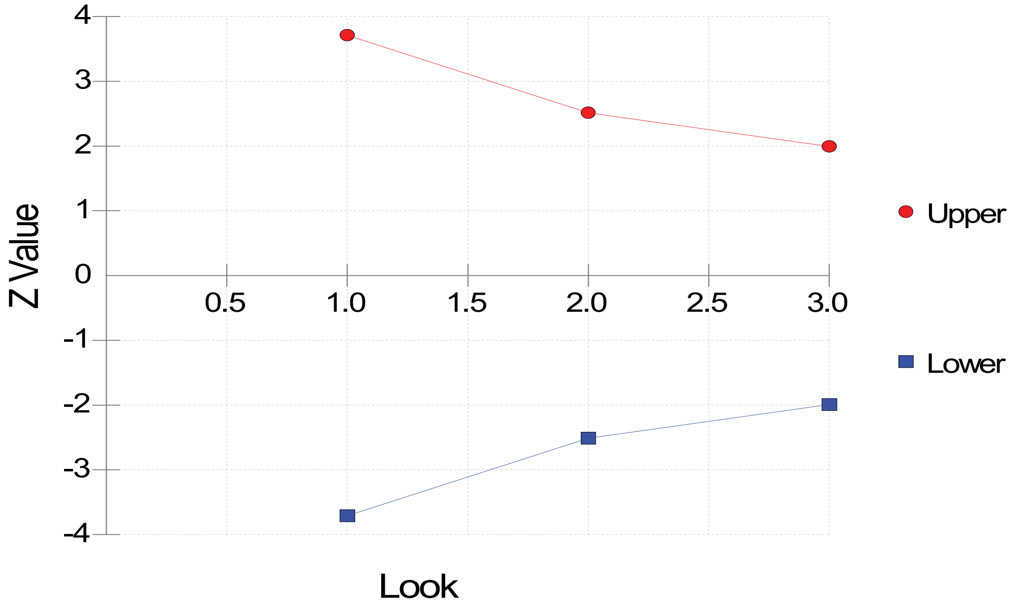

The used multiplier of 1.75 has been determined based on the equation of (1 + (*ň* - I) *ꝓ),* where *ň* = average cluster size) and *(ꝓ* = estimated intra-cluster correlation coefficient). Assuming a 1:1 randomization of equal cluster sizes, with 32 clusters of 16 patients each, with an estimated intra-cluster correlation coefficient (ICC) of 0.05, (as recommended when no previous literature or similar trials involving patient-level outcomes exist, [109-111]. With a total of 564 patients needed for this trail, on average, over the 3.5 years of enrollment for the trial, each participating center of will need to enroll 20-30 patients per year. We will enroll 564 subjects in order to ensure that we reach our goal of 504 evaluable subjects for our per-protocol analysis.

9. Missing Data: As a general strategy for missing data in this study we will concentrate highly on tactical approach rather than only analytical. Our goal is to focus on preventing missing data as much as possible. There is no methodology that can recover the robustness and unbiased character of estimates derived from a complete set of data.

We expect very little missing data due to the nature of our study design and with related heavy preparation and efforts from our parts to have a well-conducted clinical trial. The study intervention in this study will span a short duration in mostly prehospital phase therefore we do not expect a considerable drop out during that treatment phase. We expect about 2.5%-3% on average. We have inflated our sample size by 5% to reduce related effect on power adequacy.

In this study we will be attempting to maximize the number of participants according to the study protocol until all outcome data are collected. Our outcome measurements will be attempted in all subjects who initially are enrolled into the study including those who did not complete the study and in a full 'intention to treat' basis for our statistical analysis.

For 30-day mortality, given the transient nature of many of the subjects, extensive efforts will be made to ascertain vital status. Batch searches of mortality databases will continue annually for subjects with unknown status, until 30 days post trial closeout. If discharge occurs before hospital day 30 and the subject is discharged to a hospice, nursing home or other healthcare provider, research staff will contact the facility to ascertain the subject's vital status. If the subject was discharged to his/her usual residence before day 30, the research staff will contact the subject or their family/legally authorized representative (LAR). If vital status remains unknown the clinical site will request periodic searches for the subject's social security number in the Social Security Master Death Index. For subjects not reported as deceased by these sources by day 30 following ED admission, batch searches of the mortality database will continue annually until trial close-out. Date (and cause of death when available) for out-of-hospital deaths will be documented; however, underlying and contributing causes of death may not be available from these sources. For interim and final analyses, subjects who have not been reported as deceased by day 30 following ED admission from any of these sources, we will use multiple imputation for the final value. For sensitivity analyses we will report the data with and without imputation. We also will report an analysis consistent with that used in other trauma studies counting those missing as 'alive' and 'dead' on day 30.

Data missing will be accurately documented with related causes, continuously monitored and mitigated accordingly. We are not expecting missing baseline data as prehospital data collection will be protocolled and complete, therefore no problem should be expected in the precision of our analysis. We are setting a minimum rate of completion for the study primary outcomes data equal to 80%. A rate above 15% of missing primary outcome data is unacceptable for our data analysis and will be reported as a descriptive outcome only. Single imputation methods will not be used as the primary approach in the treatment of missing data.

We will assume data missing is not at random in our trial and we will use all baseline covariates and some of missing data might be determined by some observed outcome as trial progress. A likelihood-based analysis including regression multiple imputations and random-effects regression models could be implemented in this regard. Missing outcome can be predicted from individuals' observed data using model based on observed individuals. In our final analysis we will explicitly state the assumptions underlying treating missing outcomes

and justifying those using compressive data descriptions and sensitivity analysis. Our sensitivity analysis will allow us to explore the robustness of conclusions to alternative plausible assumptions. We will follow CONSORT statement in reporting the number of clusters/objects with missing outcome data by treatment arm.[112] All methods used in treating missing data will be adequately reported.

XII. Safety Monitoring

1. Adverse Event definitions:

a. Adverse event means any untoward medical occurrence associated with the use of the drug in humans, whether or not considered drug related.

b. Adverse reaction means any adverse event caused by a drug.

c. Suspected adverse reaction means any adverse event for which there is a reasonable possibility that the drug caused the adverse event. Suspected adverse reaction implies a lesser degree of certainty about causality than "adverse reaction"

d. Reasonable possibility. For the purpose of IND safety reporting, "reasonable possibility" means there is evidence to suggest a causal relationship between the drug and the adverse event.

e. Life-threatening, suspected adverse reaction. A suspected adverse reaction is considered "life-threatening" if, in the view of either the Investigator (i.e., the study site principal investigator) or Sponsor, its occurrence places the patient or research subject at immediate risk of death. It does not include a suspected adverse reaction that had it occurred in a more severe form, might have caused death.

f. Serious, suspected adverse reaction. A suspected adverse reaction is considered "serious" if, in the view of the Investigator (i.e., the study site principal investigator) or Sponsor, it results in any of the following outcomes: death, a life-threatening adverse reaction, inpatient hospitalization or prolongation of existing hospitalization, a persistent or significant incapacity or substantial disruption of the ability to conduct normal life functions, or a congenital anomaly/birth defect.

Important drug-related medical events that may not result in death, be life-threatening, or require hospitalization may be considered "serious" when, based upon appropriate medical judgment, they may jeopardize the research subject and may require medical or surgical intervention to prevent one of the outcomes listed in this definition. Examples of such medical events include allergic bronchospasm requiring intensive treatment in the emergency room or at home, blood dyscrasias or convulsions that do not result in inpatient hospitalization, or the development of drug dependency or drug abuse.

g. Unexpected, suspected adverse reaction. A suspected adverse reaction is considered "unexpected" if it is not listed in the general investigational plan, clinical protocol, or elsewhere in the current IND application; or is not listed at the specificity or severity that has been previously observed and/or specified.

2. Assessing and Reporting Adverse Events (AEs): All adverse events will be documented by the study sites and assessed for relationship to the study intervention. Reporting forms will be submitted to the Coordinating Center (to include the IND Sponsor) and Data Safety Monitoring Board (DSMB***).*** All reported adverse events will be reviewed as to treatment arm and further classified by: a) Severity (serious or non-serious); and b) Expected vs. Unexpected. For serious, unexpected adverse events felt to be associated with the research intervention, the Coordinating Center will notify the reviewing IRBs and the FDA/IND application in accordance with requisite reporting time frames.

Investigators and study team will determine daily if any clinical adverse experiences occur during the period from enrollment. The investigator will evaluate any changes in laboratory values and physical signs and will determine if the change is clinically important and different from what is expected in the course of treatment. If reportable adverse experiences occur, they will be recorded on the adverse event case report form. The study population is expected to have a large number of unrelated, expected serious adverse events including death from trauma related injuries. The SAE will be recorded on the subject's AE/SAE log and follow local reporting requirements.

SAE reporting for the PAMPer study will follow the FDA guidance on safety reporting requirements for IND and Department of Defense guidelines as well as local IRB reporting guidelines. SAE's will be reported within 15 calendar days of receiving the site report.

Transfusion services at each respective enrolling center will rely upon their respective central blood bank to provide them universal donor 'AB' plasma. All participating blood centers will minimize multiparous female donor plasma as is current standard of practice for blood banks. The risk of complications including TRALI and are required to be specifically monitored for, recorded and investigated by every transfusion service at each enrolling center. Any transfusion complication including any transfusion of blood products in the pre-hospital setting will be similarly monitored and documented. As the intervention is specific to the pre-hospital setting and since transfusion complications are temporally related to the specific transfusion, all transfusion related complications will be assessed during the initial 24 hours from arrival and recorded. <u>The admitting hospital and their respective blood bank transfusion service, which will be the service which provides the plasma for the trial and is a participating center, will be responsible for investigating and documenting any adverse reaction or fatality due to plasma that was transfused during transport.</u>

Prehospital SOPs for blood storage, temperature monitoring, administration, and adverse event reporting will be followed across all participating trauma centers. (**see Appendix 4**)

A summary report of the DSMB's findings will be submitted to regulatory agencies. At least one specialized clinician from the Data Safety Monitoring committee will be responsible for monitoring data safety. All related unanticipated problems will be directly handled by study site Investigators and reported accordingly. We will also follow Department Of Defense Unique requirements documentation. The University of Pittsburgh and each participating center will have an AE logbook to record and to assure adequate attention for continuous assessment, analysis, and reporting of adverse effects using a standardized report form (i.e., Form FDA 3500A). The Coordinating Center will be responsible for all oversight of these risk assessments with monthly evaluations.

<u>3. Prehospital Blood Product Adverse Events and Look Back SOP:</u>

a.. Transfusion rate will be compatible with the patient's condition. The patient will be monitored closely during the entire transfusion. The documented start and stop times are directly related to the actual transfusion of the component. Paramedics will document vital signs and start times in the field. Study coordinators will assume responsibility of additional vital signs and stopping time of the plasma.

b. The patient medical record shall include the following:

1. Name of the components transfused
2. Donor identification number of components
3. Date and time of transfusion (Start and Stop time)
4. Pre and post transfusion vital signs
5. The volumeor # of units transfused
6. The transfusionist's name (paramedic)
7. Documentation of related adverse events

c. Procedure for transfusion reactions:

This is modified from UPMC policy for Blood Transfusions to be applicable for this study.

1. Careful observation throughout the transfusion allows for early detection of adverse reactions and optimal treatment, if necessary. All reactions should be handled initially as possible hemolytic reactions and the transfusion must be stopped. Any adverse events associated with the transfusion of blood or blood components should be documented in the patient's Medical Record and reported to the blood bank/ transfusion service. Prehospital providers initiating transfusion of blood products will monitor vitals throughout transport. If clinical concern for a transfusion reaction occurs, the transfusion will be stopped, and supportive care will continue. The concern for a transfusion reaction will then be communicated to the trauma center staff.

2. The most common clinical events accompanying or announcing transfusion reactions are, in order of decreasing frequency:

a. Fever, with or without chills
b. Skin symptoms, hives and/or itching or rash
c. Chest pain
d. Hypotension
e. Nausea
f. Flushing
g. Respiratory Distress (wheezing, coughing or dyspnea)
h. Bleeding at infusion site
i. Hemoglobinuria
j. Circulatory overload
k. Anaphylaxis

3. If an adverse reaction is suspected, the procedure below will be followed:

a. Stop the transfusion
b. Maintain IV access with Normal Saline and change the tubing.
c. Notify the patient's physician upon arrival to the ED and initiate immediate treatment as ordered.

4. For all other blood products involved in a reaction, the transfusion shall be stopped and a Transfusion reaction investigation shall be initiated per standard blood bank guidelines

5. Notify the Blood Bank of the suspected transfusion reaction.

6. Collect a sample drawn from the patient as soon after the reaction was detected. Send a 6 mls pink top tube, labeled with a new Blood Bank armband to the Blood Bank along with the unused blood, blood bag with attached hard back copy of the transfusion tag, the IV tubing used and the top 2 copies of the Transfusion Reaction Investigation 3 part form. The back copy of the Transfusion Reaction Investigation form should remain in the patient's chart as the initial report. A post transfusion reaction Urinalysis with Microscopic may also be ordered by the patient's physician.

7. The Blood Bank will complete the Transfusion Reaction initial report and notify the caregiver of the critical results. Pathology will evaluate the patient's reactions, Blood Bank's initial report, culture when indicated, and report will be documented in the patient's medical record. Consultation between the Medical Director of the Transfusion Service, the patient's physician and Risk Management is required when a fatal hemolytic transfusion reaction occurs. Further evaluation and FDA notification may be indicated. The participating centers Transfusion Service is responsible for peer review and blood utilization practice.

8. Look back procedures: Since the plasma will be tracked through the participating centers Blood Bank/Transfusion service look back/product recall procedures will be conducted as per standard protocol. (**See Appendix 5**)

4. Data Safety Monitoring Board (DSMB): A Data and Safety Monitoring Board (DSMB) will be created to review this study and provide recommendations re. study continuation to the IND Sponsor. After initial approval and at periodic intervals (to be determined by the committee) during the course of the study, the DSMB responsibilities are to:

a. Review the research protocol, informed consent documents and plans for data and safety monitoring;

b. Evaluate the progress of the study, including periodic assessments of data quality and timeliness, participant recruitment, accrual and retention, participant risk versus benefit, adverse events, unanticipated problems, performance of the trial sites, and other factors that can affect study outcome;

c. Consider factors external to the study when relevant information becomes available, such as scientific or therapeutic developments that may have an impact on the safety of the participants or the ethics of the study;

d. Review clinical center performance, make recommendations and assist in the resolution of problems reported by the IND Sponsor or study site Investigators;

e. Protect the safety of the study participants;

f. Report on the safety and progress of the study;

g. Make recommendations to the IND Sponsor, and if required, to the FDA concerning continuation, termination or other modifications of the study based on the observed beneficial or adverse effects of the treatment under study;

h. Monitor the confidentiality of the study data and the results of monitoring;

i. Assist the IND Sponsor by commenting on any problems with study conduct, enrollment, sample size and/or data collection.

j. The DSMB will include experts in emergency medicine, surgery (trauma/critical medicine),bioethics and biostatistics. As a condition of Department of Defense funding, a Medical Monitor will be appointed and approved by the IRB. The Medical Monitor may or may not be a DSMB member. Members will consist of persons independent of the investigators who have no financial, scientific, or other conflict of interest with the study. Written documentation attesting to absence of conflict of interest will be required.

k. The University of Pittsburgh Office of Clinical Research, Health Sciences will provide the logistical management and support of the DSMB. A safety officer (chairperson) will be identified at the first meeting. This person will be the contact person for serious adverse event reporting. Procedures for this will be discussed at the first meeting.

l. The first meeting will take place before initiation of the study to discuss the protocol, approve the commencement of the study, and to establish guidelines to monitor the study. The follow-up meeting frequency of the DSMB will be determined during the first meeting. An emergency meeting of the DSMB will be called at any time by the Chairperson should questions of patient safety arise. DSMB charter is attached (**Appendix 6).**

5. Research Monitor

The research monitor is required to review all unanticipated problems involving risk to subjects or others, serious adverse events and all subject deaths and provide an unbiased written report of the event. At a minimum, the medical monitor must comment on the outcomes of the event or problem and in case of a serious adverse event or death, comment on the relationship to participation in the study. The medical monitor must also indicate whether he concurs with the details of the report provided by the principal investigator. The research monitor may also discuss the research protocol with the investigators, interview human subjects, and consult with others outside of the study about the research. The research monitor shall have authority to stop a research protocol in progress, remove individual human subjects from a research protocol, and take whatever steps necessary to protect the safety and well-being of human subjects until the IRB can assess the monitor's report. Reports for events determined by either the investigator or research monitor to be possibly or definitely related to participation and reports of events resulting in death must be promptly forwarded to the U.S. Army Medical Research and Materiel Command Office of Research Protections Human Research Protections Office (USAMRMC ORP HRPO).

6. Interim Analyses: In concert with the DSMB, prior to initiation of the trial, the final monitoring plan will be developed to serve as the guide to the DSMB's decision-making process concerning early stopping of the trial. In making the decision to recommend termination of the study, the DSMB shall be guided by several types of information: (i) a formal stopping rule based on the primary analysis (comparison of treatment groups on the 30 day mortality), (ii) information on safety outcomes by treatment group, (iii) consistency between results for primary and secondary outcomes, and (iv) consistency of treatment effects across subgroups.

We have designed this trial with a two interim look before the final analysis. Our power analysis generated assuming a total of 3 sequential tests based on O'Brien-Fleming spending function to determine alpha spending and test boundaries. We will use test of proportions differences, z-test with continuity correction applied and other adjusting techniques. The level of significance will maintain an overall p value of 0.05 according to O'Brien-Fleming stopping boundaries leaving a p value of 0.038; two sided, for the final analysis

with a final z-value of 1.993. An independent data and safety monitoring board (DSMB) will periodically review the efficacy and safety data. DSMB will issue related recommendations based on comprehensive data monitoring and substantiated evidences. Two formal interim analyses of efficacy will be performed when 33% and 67% of the expected number of primary events had accrued (about one month after 1/3 and 2/3 of subject accruals). The purpose of our sequential tests is to detect early sign of superior efficacy and detect further apparent futility in the intervention group. This kind of futility monitoring and testing could cause this trial to be stopped as soon as a negative outcome of 30-days mortality is inevitable and thus it is no longer worthwhile continuing the trial to its completion. Such early termination for futility could reduce the enormous expenditures of resources, human and financial, involved in the conduct of trials that ultimately provides negative answers regarding the value of the study medical intervention.[113]

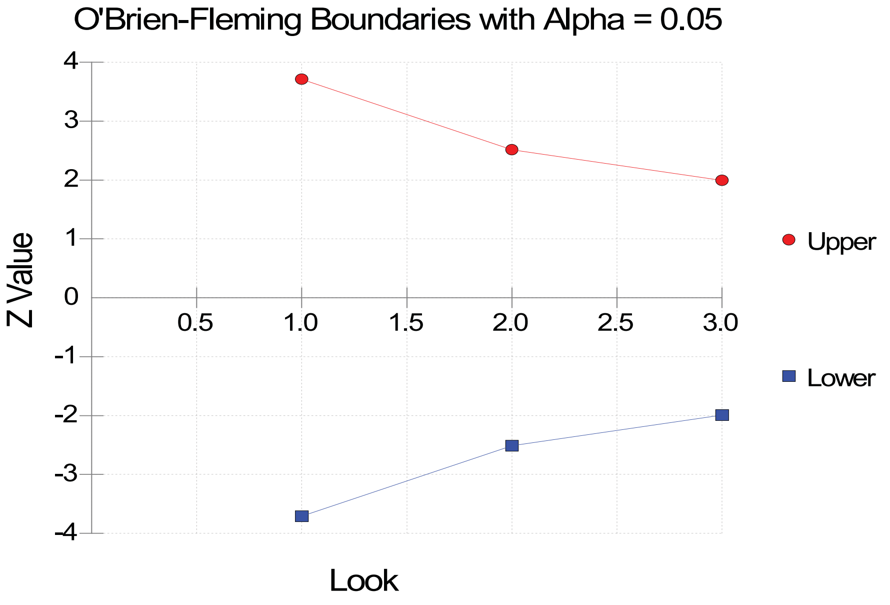

Our trial's lower and upper stopping boundaries have been computed to ensure that the trial Type I and Type II error probabilities of the group sequential plan are according to the study assumptions and design. The upper boundaries are related to the formal efficacy testing at each assigned sample size (expected number of primary events completion at 33%, 67%, and 100%). The lower boundaries are related to the formal futility (safety) testing at each assigned sample size (expected number of primary events completion at 33%, 67%, and 100%). Upper and lower boundaries will be provided to DSMB as a guideline and could be modified by DSMB prior to the trial upon reasonable justifications. With this sequential testing plan based on O'Brien-Fleming spending function, only an absolutely overwhelming treatment intervention can justify the termination of our clinical trial after a third of the subjects have been enrolled and completed a one month of follow up. If the trial has been ordered to stop early because of interim analysis, adjusted p-values will be computed based on the described analysis of our main clinical outcome. Unadjusted p-value will not be considered for final results interpretations.

Our interim analysis is part of our three sequential testing as we have mentioned above. At each of the two interim looks, 30 days-mortality will be pooled across clusters comparing between the two study groups. Based on the assumed power analysis at each interim look, the z-value will be calculated and adjusted for cluster size and other adjustments (including within-clusters correlation) similar of what we have described on the analysis of our primary clinical outcome and as much as the accrued sample size at that specific check may allowed. We have illustrated z-values for the upper and lower boundaries across interim two analyses and final analysis of a total accumulated alpha of 0.05. (**Figure and Table below**)

Further and in relation to interim safety analysis, safety data by study groups labeled as Plasma group and Control group will be provided periodically to DSMB. Data will be remained completely unblinded unless the DSMB call for otherwise. The safety data of the study include serious adverse events regarding frequency, anticipated or unanticipated, individual description for each event and dates. Our periodic reports to DSMB will include as well data on recruitment, data completion, data quality, etc. Other data will be provided as well as any additional safety analysis upon DSMB request. Mortality will be reported as an overall in our periodic reports to DSMB however we will report mortality individually as treatment A and B at each of the trial two formal interim analyses. Secondary to limited funding, complex cluster design and multicenter nature of the trial, we are unable use adaptive design due to the possibility of substantial increase in sample size, however based upon a DSMB recommendation during our interim analysis if our mortality rate is lower than expected and requires less than a 10% increase in our pre-determined sample size required, we will attempt to increase our enrollment utilizing those centers with highest enrollment, limited by budgetary feasibility, and taking into consideration the needed adjustment for type I error.

Sequential tests based on O'Brien-Fleming spending function, alpha spending and test boundaries

Look	time	Lower Boundary	Upper boundary	Nominal Alpha	Inc Alpha *Spent Alpha*	Total Alpha	Inc Power *Exit probabilities*	Total Power
1	0.33	-3.71030	3.71030	0.000207	0.000207	0.000207	0.030172	0.030172
2	0.67	-2.51142	2.51142	0.012025	0.011890	0.012097	0.501816	0.531988
3	1.00	-1.99302	1.99302	0.046259	0.037903	0.05000	0.351816	0.883804

As an alternative to the above methods to monitor futility, the DSMB might want to terminate our trial when the results of the interim analysis are unlikely to change after accruing more patients based on conditional power. Conditional power is defined as the approach that quantifies the statistical power to yield an answer different from that seen at the interim analysis. If this quantity is really small, then we can conclude that it would be futile to continue with the investigation.

For such a conditional power calculation, we will use z to determine if we can reject our H0 with related interim alpha according to the following:

If the current trend continues, what is the chance that we will have a positive study (Final Z ≥ 1.96)

$$z = \frac{\text{Actual mortality at this current interim} - \textit{Total current accrual at this interim analysis}}{\sqrt{\text{Total calculated sample size } (\textit{expected rate of mortality})(\text{expected rate of survival})}} \geq 1.96$$

We will be able to tell how many remaining subjects should survive in order to reject H0. Also we can be able to determine if the probability is so small to reject the H0 according to accumulated data. Using the above information we can decide that if it is futile to continue because there is such a small chance of rejecting the trial H0.

For futility assessment based on conditional power we will be able to use SAS programs (FindZa.sas and FindZaBI.sas) at each interim analysis to compute the properties of a futility assessment based on a conditional power computation.

Based on the above analysis the study DSMB might provide recommendations at interim analysis for early discontinuation of the trial because of futility and related conditional power. Such conclusion is based on a fact that even further subject enrollments to a full sample size or even additional increase in sample size will unlikely proves effectiveness. Possible additional confirmatory analysis can be applied to consider any other efficacy measures and to provide subsets analysis based on centers and other considerations.

7. Quality Control, Assurance and Confidentiality:

a. Protocol Compliance:

The participating study site Investigators will not deviate from the protocol for any reason without prior written approval from the IRB except in the event of the safety of the research subject. In that event, the study site Investigator will notify the IND Sponsor and reviewing IRB immediately, if possible, and request approval of the protocol deviation, or, if prospective IND Sponsor and IRB approval is not possible, the study site Investigator will notify the IND Sponsor and reviewing IRB promptly following the respective protocol deviation. The study site Investigator will inform the reviewing IRB of all protocol deviations and unanticipated events involving risks to the research subjects and others, and will obtain prospective IRB approval for all proposed protocol changes. Persistent or serious noncompliance may result in termination of the study site's participation in the research study.

Protocol Deviations: Due to the prehospital setting of the intervention, the relative focused inclusion criteria, and the short intervention period, we expect few protocol deviations as compared to other large multicenter trials. If monitoring reports demonstrate evidence of continuing protocol deviations, we will analyze them to

determine if they are site specific or common across the study. We will note if specific inclusion or exclusion criteria are being misinterpreted, if a certain time point in testing is being omitted, or if a common set of data elements are missing. If the deviations are site specific, retraining will be done at the site. If the problems are study wide, we will discuss them with the other investigators, the DOD and the FDA to see if the protocol needs amended or recruitment put on hold.

b. Privacy and Confidentiality:
The study site Investigator's and members of their research team will make reasonable effort to ensure the research subjects' confidentiality. Subject name and other identifiable information will be kept in a secure, locked, limited access area.

c. Investigator Responsibilities:
The study site Investigators will agree to implement the IRB approved protocol and conduct the study in accordance with Section 9 (Commitments) of Form FDA 1572, 21 CFR Part 312, Subpart D, and the ICH GCP Guidelines (E6, Section 5) as well as all applicable national, state and local laws. The study will be performed in accordance with ethical principles that have their origin in the Declaration of Helsinki and are consistent with ICH/Good Clinical Practice, and applicable regulatory requirements.

XIII. Study Limitations

Due to the scarce nature of universal donor AB plasma or low titer anti-B A plasma and logistics of the air medical transport intervention, randomization for the proposal will be assigned in a 2, 4, or 6 month block fashion for helicopters or airbases at each respective participating institution. Individual patient randomization, requiring AB plasma or low titer anti-B A plasma to be aboard or available at every helicopter or airbase constantly throughout enrollment, remains the optimal randomization method, however is not feasible. This will bring in the potential for disparities in enrollment to either the intervention arm or standard care arm as we will not be able to assure equal numbers in each arm of the study.
The study is a multi-center trial with the potential for variation in prehospital standard of care and in-hospital variation in post-injury care potentially affecting the primary and secondary outcomes for the proposal. To maximize the generalizability of the trial results and to minimize procedural requirements in the prehospital setting, we elected not to standardize prehospital air medical standard of care except for crystalloid infusion. Importantly, we selected similar academic, level 1, participating centers based upon their patient and air medical transport volumes, their prior experience with clinical research and prior participation in prior multi-center trials, and who practice up-to-date evidence based trauma care, in attempts to minimize significant variation in post injury care.

XIV. Timetable

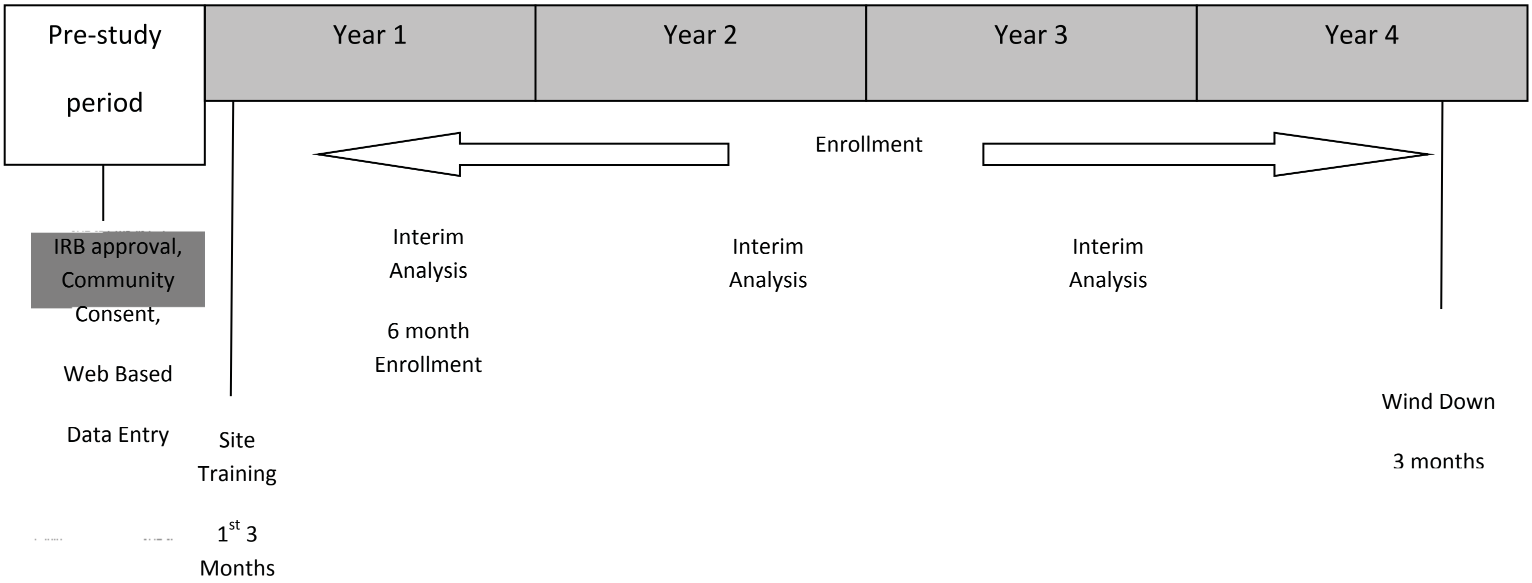

XV. Additional Regulatory and Reporting Requirements Of The USAMRMC

Additional reporting requirements and responsibilities of the Principal Investigator to the United States Army Medical Research and Materiel Command's (USAMRMC) Office of Research Protections (ORP), Human Research Protection Office (HRPO) that will be employed can be found in the Appendices. (**Clinical Protocol Appendix 7**)

XVI. Bibliography and References/ Literature Review

1. Kauvar, D.S., R. Lefering, and C.E. Wade, *Impact of hemorrhage on trauma outcome: an overview of epidemiology, clinical presentations, and therapeutic considerations.* J Trauma, 2006. 60(6 Suppl): p. S3-11.
2. Hoyt, D.B., *A clinical review of bleeding dilemmas in trauma.* Semin Hematol, 2004. 41(1 Suppl 1): p. 40-3.
3. Holcomb, J.B., *Methods for improved hemorrhage control.* Crit Care, 2004. 8 Suppl 2: p. S57-60.
4. Shackford, S.R., et al., *The epidemiology of traumatic death. A population-based analysis.* Arch Surg, 1993. 128(5): p. 571-5.
5. Gentilello, L.M. and D.J. Pierson, *Trauma critical care.* Am J Respir Crit Care Med, 2001. 163(3 Pt 1): p. 604-7.
6. Wilson, R.F., et al., *Problems with 20 or more blood transfusions in 24 hours.* Am Surg, 1987. 53(7): p. 410-7.
7. Ferrara, A., et al., *Hypothermia and acidosis worsen coagulopathy in the patient requiring massive transfusion.* Am J Surg, 1990. 160(5): p. 515-8.
8. Moore, E.E., *Thomas G. Orr Memorial Lecture. Staged laparotomy for the hypothermia, acidosis, and coagulopathy syndrome.* Am J Surg, 1996. 172(5): p. 405-10.
9. Cosgriff, N., et al., *Predicting life-threatening coagulopathy in the massively transfused trauma patient: hypothermia and acidoses revisited.* J Trauma, 1997. 42(5): p. 857-61; discussion 861-2.
10. Patt, A., B.L. McCroskey, and E.E. Moore, *Hypothermia-induced coagulopathies in trauma.* Surg Clin North Am, 1988. 68(4): p. 775-85.
11. Dunn, E.L., et al., *Acidosis-induced coagulopathy.* Surg Forum, 1979. 30: p. 471-3.
12. Shaz, B.H., et al., *Pathophysiology of Early Trauma-Induced Coagulopathy: Emerging Evidence for Hemodilution and Coagulation Factor Depletion.* J Trauma, 2011.
13. Curry, N., et al., *The acute management of trauma hemorrhage: a systematic review of randomized controlled trials.* Crit Care, 2011. 15(2): p. R92.
14. Curry, N., et al., *Trauma-Induced Coagulopathy-A Review of the Systematic Reviews: Is There Sufficient Evidence to Guide Clinical Transfusion Practice?* Transfus Med Rev, 2011.
15. MacLeod, J., et al., *Predictors of mortality in trauma patients.* Am Surg, 2004. 70(9): p. 805-10.
16. MacLeod, J.B., et al., *Early coagulopathy predicts mortality in trauma.* J Trauma, 2003. 55(1): p. 39-44.
17. Brohi, K., et al., *Acute traumatic coagulopathy.* J Trauma, 2003. 54(6): p. 1127-30.
18. Maegele, M., et al., *Early coagulopathy in multiple injury: an analysis from the German Trauma Registry on 8724 patients.* Injury, 2007. 38(3): p. 298-304.

19. Niles, S.E., et al., *Increased mortality associated with the early coagulopathy of trauma in combat casualties.* J Trauma, 2008. 64(6): p. 1459-63; discussion 1463-5.
20. Brohi, K., et al., *Acute traumatic coagulopathy: initiated by hypoperfusion: modulated through the protein C pathway?* Ann Surg, 2007. 245(5): p. 812-8.
21. Gubler, K.D., et al., *The impact of hypothermia on dilutional coagulopathy.* J Trauma, 1994. 36(6): p. 847-51.
22. MacLeod, J.B., *Trauma and coagulopathy: a new paradigm to consider.* Arch Surg, 2008. 143(8): p. 797-801.
23. Gando, S., et al., *Disseminated intravascular coagulation is a frequent complication of systemic inflammatory response syndrome.* Thromb Haemost, 1996. 75(2): p. 224-8.
24. Brohi, K., M.J. Cohen, and R.A. Davenport, *Acute coagulopathy of trauma: mechanism, identification and effect.* Curr Opin Crit Care, 2007. 13(6): p. 680-5.
25. Nascimento, B., et al., *Clinical review: Fresh frozen plasma in massive bleedings - more questions than answers.* Crit Care, 2010. 14(1): p. 202.
26. Hess, J.R., et al., *The coagulopathy of trauma: a review of mechanisms.* J Trauma, 2008. 65(4): p. 748-54.
27. Kashuk, J.L., et al., *Postinjury coagulopathy management: goal directed resuscitation via POC thrombelastography.* Ann Surg, 2010. 251(4): p. 604-14.
28. Spahn, D.R. and R. Rossaint, *Coagulopathy and blood component transfusion in trauma.* Br J Anaesth, 2005. 95(2): p. 130-9.
29. Lucas, C.E. and A.M. Ledgerwood, *Clinical significance of altered coagulation tests after massive transfusion for trauma.* Am Surg, 1981. 47(3): p. 125-30.
30. Murray, D., B. Pennell, and J. Olson, *Variability of prothrombin time and activated partial thromboplastin time in the diagnosis of increased surgical bleeding.* Transfusion, 1999. 39(1): p. 56-62.
31. Kashuk, J.L., et al., *Noncitrated whole blood is optimal for evaluation of postinjury coagulopathy with point-of-care rapid thrombelastography.* J Surg Res, 2009. 156(1): p. 133-8.
32. Carroll, R.C., et al., *Early evaluation of acute traumatic coagulopathy by thrombelastography.* Transl Res, 2009. 154(1): p. 34-9.
33. Jeger, V., H. Zimmermann, and A.K. Exadaktylos, *Can RapidTEG accelerate the search for coagulopathies in the patient with multiple injuries?* J Trauma, 2009. 66(4): p. 1253-7.
34. Doran, C.M., T. Woolley, and M.J. Midwinter, *Feasibility of using rotational thromboelastometry to assess coagulation status of combat casualties in a deployed setting.* J Trauma, 2010. 69 Suppl 1: p. S40-8.
35. Claridge, J.A., et al., *Blood transfusions correlate with infections in trauma patients in a dose-dependent manner.* Am Surg, 2002. 68(7): p. 566-72.
36. Malone, D.L., et al., *Blood transfusion, independent of shock severity, is associated with worse outcome in trauma.* J Trauma, 2003. 54(5): p. 898-905; discussion 905-7.
37. Moore, F.A., E.E. Moore, and A. Sauaia, *Blood transfusion. An independent risk factor for postinjury multiple organ failure.* Arch Surg, 1997. 132(6): p. 620-4; discussion 624-5.

38. Robinson, W.P., 3rd, et al., *Blood transfusion is an independent predictor of increased mortality in nonoperatively managed blunt hepatic and splenic injuries.* J Trauma, 2005. 58(3): p. 437-44; discussion 444-5.
39. Khan, H., et al., *Fresh-frozen plasma and platelet transfusions are associated with development of acute lung injury in critically ill medical patients.* Chest, 2007. 131(5): p. 1308-14.
40. Huber-Wagner, S., et al., *Massive blood transfusion and outcome in 1062 polytrauma patients: a prospective study based on the Trauma Registry of the German Trauma Society.* Vox Sang, 2007. 92(1): p. 69-78.
41. Mitra, B., et al., *Massive blood transfusion and trauma resuscitation.* Injury, 2007. 38(9): p. 1023-9.
42. Malone, D.L., J.R. Hess, and A. Fingerhut, *Massive transfusion practices around the globe and a suggestion for a common massive transfusion protocol.* J Trauma, 2006. 60(6 Suppl): p. S91-6.
43. Holcomb, J.B., *Damage control resuscitation.* J Trauma, 2007. 62(6 Suppl): p. S36-7.
44. Holcomb, J.B., et al., *Damage control resuscitation: directly addressing the early coagulopathy of trauma.* J Trauma, 2007. 62(2): p. 307-10.
45. Ketchum, L., J.R. Hess, and S. Hiippala, *Indications for early fresh frozen plasma, cryoprecipitate, and platelet transfusion in trauma.* J Trauma, 2006. 60(6 Suppl): p. S51-8.
46. Borgman, M.A., et al., *The ratio of blood products transfused affects mortality in patients receiving massive transfusions at a combat support hospital.* J Trauma, 2007. 63(4): p. 805-13.
47. Sperry, J.L., et al., *An FFP:PRBC transfusion ratio >/=1:1.5 is associated with a lower risk of mortality after massive transfusion.* J Trauma, 2008. 65(5): p. 986-93.
48. Fox, C.J., et al., *The effectiveness of a damage control resuscitation strategy for vascular injury in a combat support hospital: results of a case control study.* J Trauma, 2008. 64(2 Suppl): p. S99-106; discussion S106-7.
49. Gunter, O.L., Jr., et al., *Optimizing outcomes in damage control resuscitation: identifying blood product ratios associated with improved survival.* J Trauma, 2008. 65(3): p. 527-34.
50. Cotton, B.A., et al., *Damage control hematology: the impact of a trauma exsanguination protocol on survival and blood product utilization.* J Trauma, 2008. 64(5): p. 1177-82; discussion 1182-3.
51. Zink, K.A., et al., *A high ratio of plasma and platelets to packed red blood cells in the first 6 hours of massive transfusion improves outcomes in a large multicenter study.* Am J Surg, 2009. 197(5): p. 565-70; discussion 570.
52. Inaba, K., et al., *The impact of platelet transfusion in massively transfused trauma patients.* J Am Coll Surg, 2010. 211(5): p. 573-9.
53. Holcomb, J.B., et al., *Increased plasma and platelet to red blood cell ratios improves outcome in 466 massively transfused civilian trauma patients.* Ann Surg, 2008. 248(3): p. 447-58.
54. Riskin, D.J., et al., *Massive transfusion protocols: the role of aggressive resuscitation versus product ratio in mortality reduction.* J Am Coll Surg, 2009. 209(2): p. 198-205.

55. O'Keeffe, T., et al., *A massive transfusion protocol to decrease blood component use and costs.* Arch Surg, 2008. 143(7): p. 686-90; discussion 690-1.
56. Magnotti, L.J., et al., *Improved survival after hemostatic resuscitation: does the emperor have no clothes?* J Trauma, 2011. 70(1): p. 97-102.
57. Snyder, C.W., et al., *The relationship of blood product ratio to mortality: survival benefit or survival bias?* J Trauma, 2009. 66(2): p. 358-62; discussion 362-4.
58. Kashuk, J.L., et al., *Postinjury life threatening coagulopathy: is 1:1 fresh frozen plasma:packed red blood cells the answer?* J Trauma, 2008. 65(2): p. 261-70; discussion 270-1.
59. Scalea, T.M., et al., *Early aggressive use of fresh frozen plasma does not improve outcome in critically injured trauma patients.* Ann Surg, 2008. 248(4): p. 578-84.
60. Holcomb, J.B., *Optimal use of blood products in severely injured trauma patients.* Hematology Am Soc Hematol Educ Program, 2010. 2010: p. 465-9.
61. Yucel, N., et al., *Trauma Associated Severe Hemorrhage (TASH)-Score: probability of mass transfusion as surrogate for life threatening hemorrhage after multiple trauma.* J Trauma, 2006. 60(6): p. 1228-36; discussion 1236-7.
62. Larson, C.R., et al., *Association of shock, coagulopathy, and initial vital signs with massive transfusion in combat casualties.* J Trauma, 2010. 69 Suppl 1: p. S26-32.
63. Schreiber, M.A., et al., *Early predictors of massive transfusion in combat casualties.* J Am Coll Surg, 2007. 205(4): p. 541-5.
64. McLaughlin, D.F., et al., *A predictive model for massive transfusion in combat casualty patients.* J Trauma, 2008. 64(2 Suppl): p. S57-63; discussion S63.
65. Dutton, R.P., R. Lefering, and M. Lynn, *Database predictors of transfusion and mortality.* J Trauma, 2006. 60(6 Suppl): p. S70-7.
66. Nunez, T.C., et al., *Early prediction of massive transfusion in trauma: simple as ABC (assessment of blood consumption)?* J Trauma, 2009. 66(2): p. 346-52.
67. Watson, G.A., et al., *Fresh frozen plasma is independently associated with a higher risk of multiple organ failure and acute respiratory distress syndrome.* J Trauma, 2009. 67(2): p. 221-7; discussion 228-30.
68. Spinella, P.C., et al., *Effect of plasma and red blood cell transfusions on survival in patients with combat related traumatic injuries.* J Trauma, 2008. 64(2 Suppl): p. S69-77; discussion S77-8.
69. Clarke, J.R., et al., *Time to laparotomy for intra-abdominal bleeding from trauma does affect survival for delays up to 90 minutes.* J Trauma, 2002. 52(3): p. 420-5.
70. Neal, M.D., et al., *Over reliance on computed tomography imaging in patients with severe abdominal injury: is the delay worth the risk?* J Trauma, 2011. 70(2): p. 278-84.
71. Howell, G.M., et al., *Delay to therapeutic interventional radiology postinjury: time is of the essence.* J Trauma, 2010. 68(6): p. 1296-300.
72. Brown, J.B., et al., *Helicopters improve survival in seriously injured patients requiring interfacility transfer for definitive care.* J Trauma, 2011. 70(2): p. 310-4.
73. Brown, J.B., et al., *Helicopters and the civilian trauma system: national utilization patterns demonstrate improved outcomes after traumatic injury.* J Trauma, 2010. 69(5): p. 1030-4; discussion 1034-6.

74. Dubick, M.A. and J.L. Atkins, *Small-volume fluid resuscitation for the far-forward combat environment: current concepts.* J Trauma, 2003. 54(5 Suppl): p. S43-5.
75. Mabry, R.L., et al., *United States Army Rangers in Somalia: an analysis of combat casualties on an urban battlefield.* J Trauma, 2000. 49(3): p. 515-28; discussion 528-9.
76. Lehmann, R., et al., *Interhospital patient transport by rotary wing aircraft in a combat environment: risks, adverse events, and process improvement.* J Trauma, 2009. 66(4 Suppl): p. S31-4; discussion S34-6.
77. Guyette, F.X., H. Wang, and J.S. Cole, *King airway use by air medical providers.* Prehosp Emerg Care, 2007. 11(4): p. 473-6.
78. Guyette, F.X., et al., *Feasibility of basic emergency medical technicians to perform selected advanced life support interventions.* Prehosp Emerg Care, 2006. 10(4): p. 518-21.
79. Guyette, F.X., et al., *Vasopressin administered with epinephrine is associated with a return of a pulse in out-of-hospital cardiac arrest.* Resuscitation, 2004. 63(3): p. 277-82.
80. Guyette, F., et al., *Prehospital serum lactate as a predictor of outcomes in trauma patients: a retrospective observational study.* J Trauma, 2011. 70(4): p. 782-6.
81. Sauaia, A., et al., *Epidemiology of trauma deaths: a reassessment.* J Trauma, 1995. 38(2): p. 185-93.
82. Acosta, J.A., et al., *Lethal injuries and time to death in a level I trauma center.* J Am Coll Surg, 1998. 186(5): p. 528-33.
83. Kim, B.D., et al., *The effects of prehospital plasma on patients with injury: a prehospital plasma resuscitation.* J Trauma Acute Care Surg, 2012. 73(2 Suppl 1): p. S49-53.
84. Bilello, J.F., et al., *Prehospital hypotension in blunt trauma: identifying the "crump factor".* J Trauma, 2011. 70(5): p. 1038-42.
85. Lipsky, A.M., et al., *Prehospital hypotension is a predictor of the need for an emergent, therapeutic operation in trauma patients with normal systolic blood pressure in the emergency department.* J Trauma, 2006. 61(5): p. 1228-33.
86. Lalezarzadeh, F., et al., *Evaluation of prehospital and emergency department systolic blood pressure as a predictor of in-hospital mortality.* Am Surg, 2009. 75(10): p. 1009-14.
87. Kautza, B.C., et al., *Changes in massive transfusion over time: an early shift in the right direction?* J Trauma Acute Care Surg, 2012. 72(1): p. 106-11.
88. Hebert, P.C., et al., *A multicenter, randomized, controlled clinical trial of transfusion requirements in critical care. Transfusion Requirements in Critical Care Investigators, Canadian Critical Care Trials Group.* N Engl J Med, 1999. 340(6): p. 409-17.
89. Carrico, C.J., et al., *Multiple-organ-failure syndrome.* Arch Surg, 1986. 121(2): p. 196-208.
90. Marshall, J.C., *Organ dysfunction as an outcome measure in clinical trials.* Eur J Surg Suppl, 1999(584): p. 62-7.
91. Marshall, J.C., et al., *Multiple organ dysfunction score: a reliable descriptor of a complex clinical outcome.* Crit Care Med, 1995. 23(10): p. 1638-52.

92. Bernard, G.R., et al., *The American-European Consensus Conference on ARDS. Definitions, mechanisms, relevant outcomes, and clinical trial coordination.* Am J Respir Crit Care Med, 1994. 149(3 Pt 1): p. 818-24.
93. Cuschieri, J., et al., *Early elevation in random plasma IL-6 after severe injury is associated with development of organ failure.* Shock, 2010. 34(4): p. 346-51.
94. Sperry, J.L., et al., *Male gender is associated with excessive IL-6 expression following severe injury.* J Trauma, 2008. 64(3): p. 572-8; discussion 578-9.
95. O'Brien, P.C. and T.R. Fleming, *A multiple testing procedure for clinical trials.* Biometrics, 1979. 35(3): p. 549-56.
96. Murray, D.M., ed. *The Design and Analysis of Group Randomized Trials*. Oxford University Press: Oxford.
97. Zucker, D.M., et al., *Statistical design of the Child and Adolescent Trial for Cardiovascular Health (CATCH): implications of cluster randomization.* Control Clin Trials, 1995. 16(2): p. 96-118.
98. Burton, P., L. Gurrin, and P. Sly, *Extending the simple linear regression model to account for correlated responses: an introduction to generalized estimating equations and multi-level mixed modelling.* Stat Med, 1998. 17(11): p. 1261-91.
99. Murray, D.M., S.P. Varnell, and J.L. Blitstein, *Design and analysis of group-randomized trials: a review of recent methodological developments.* Am J Public Health, 2004. 94(3): p. 423-32.
100. Hedeker, D., R.D. Gibbons, and B.R. Flay, *Random-effects regression models for clustered data with an example from smoking prevention research.* J Consult Clin Psychol, 1994. 62(4): p. 757-65.
101. Dutton, R.P., et al., *Safety of uncrossmatched type-O red cells for resuscitation from hemorrhagic shock.* J Trauma, 2005. 59(6): p. 1445-9.
102. Murthi, S.B., et al., *Transfusion medicine in trauma patients.* Expert Rev Hematol, 2008. 1(1): p. 99-109.
103. Murray, D.J., et al., *Packed red cells in acute blood loss: dilutional coagulopathy as a cause of surgical bleeding.* Anesth Analg, 1995. 80(2): p. 336-42.
104. Velmahos, G.C., et al., *Is there a limit to massive blood transfusion after severe trauma?* Arch Surg, 1998. 133(9): p. 947-52.
105. Gonzalez, E.A., et al., *Fresh frozen plasma should be given earlier to patients requiring massive transfusion.* J Trauma, 2007. 62(1): p. 112-9.
106. Heckbert, S.R., et al., *Outcome after hemorrhagic shock in trauma patients.* J Trauma, 1998. 45(3): p. 545-9.
107. Cales, R.H., *Trauma mortality in Orange County: the effect of implementation of a regional trauma system.* Ann Emerg Med, 1984. 13(1): p. 1-10.
108. Teixeira, P.G., et al., *Impact of plasma transfusion in massively transfused trauma patients.* J Trauma, 2009. 66(3): p. 693-7.
109. Lancaster, G.A., et al., *Trials in primary care: statistical issues in the design, conduct and evaluation of complex interventions.* Stat Methods Med Res, 2010. 19(4): p. 349-77.
110. Littenberg, B. and C.D. MacLean, *Intra-cluster correlation coefficients in adults with diabetes in primary care practices: the Vermont Diabetes Information System field survey.* BMC Med Res Methodol, 2006. 6: p. 20.

111. Elley, C.R., et al., *Intraclass correlation coefficients from three cluster randomised controlled trials in primary and residential health care.* Aust N Z J Public Health, 2005. 29(5): p. 461-7.
112. Campbell, M.K., D.R. Elbourne, and D.G. Altman, *CONSORT statement: extension to cluster randomised trials.* BMJ, 2004. 328(7441): p. 702-8.
113. Ware, J.H., J.E. Muller, and E. Braunwald, *The futility index. An approach to the cost-effective termination of randomized clinical trials.* Am J Med, 1985. 78(4): p. 635-43.

Clinical Protocol Appendix 1

Requirements for Exception From Consent For Emergency Research

We have outlined below each criterion stipulated in the regulations for this exception and how our study design applies to these criteria.

(1) The human subjects are in a life-threatening situation, available treatments are unproven or unsatisfactory, and the collection of valid scientific evidence, which may include evidence obtained through randomized placebo-controlled investigations, is necessary to determine the safety and effectiveness of particular interventions.

The proposed trial is a randomized trial comparing the use of prehospital plasma versus standard of care in patients in hemorrhagic shock following injury requiring air medical transport to a definitive trauma center. These patients are in an life-threatening situation with a mortality before discharge approaching 25% despite all efforts. The standard of care for management of these patients includes intravenous crystalloid while en route to definitive care. As reviewed in this proposal, prior studies have demonstrated that injured patients who require large volume blood transfusion have improved survival if transfusion of plasma in high or equal ratios to blood occurs. Evidence suggests that early blood component transfusion may reduce overall blood transfusion requirements and that addressing the coagulopathy which occurs early after injury improves outcome. Controversy remains regarding the specific ratio of plasma and other blood components relative to blood that is beneficial. It is known that plasma is associated with a greater risk of pulmonary complications, including acute lung injury and adult respiratory distress syndrome; however, whether these risks outweigh the survival benefit associated with early plasma in hemorrhagic shock patients remains unknown. Prehospital plasma use has never been characterized in civilian or military patient cohorts.

We propose a randomized trial focused on evaluation of prehospital plasma with sufficient statistical power to detect changes in clinical outcomes. Furthermore, we have developed the current proposal with also places an emphasis on the mechanism by which plasma may have any beneficial effect.

(2) Obtaining informed consent is not feasible because:
i. The subjects will not be able to give their informed consent as a result of their medical condition;
ii. The intervention under investigation must be administered before consent from the subjects' legally authorized representatives is feasible; and
iii. There is no reasonable way to identify prospectively the individuals likely to become eligible for participation in the clinical investigation.

The study intervention needs to be administered en route to a definitive trauma center from the injury scene or from a referring hospital (see discussion of therapeutic window below). In this uncontrolled setting, the hemorrhagic shock patient is unable to provide consent for study enrollment, is commonly unconscious or in extremis, and legal next-of-kin are often not immediately available at the scene, nor is it practical for the hospital provider to explain the study and receive consent while caring for the patient. Since we are studying patients with hemorrhagic shock following injury, there is no way to prospectively identify individuals who are likely to become eligible for this trial.

(3) Participation in the research holds out the prospect of direct benefit to the subjects because:
i. Subjects are facing a life-threatening situation that necessitates intervention;
ii. Appropriate animal and other preclinical studies have been conducted, and the information derived from those studies and related evidence support the potential for the intervention to provide a direct benefit to the individual subjects; and
iii. Risks associated with the investigation are reasonable in relation to what is known about the medical condition of the potential class of subjects, the risks and benefits of standard therapy, if any, and what is known about the risks and benefits of the proposed intervention or activity.

(i) As defined, these patients are injured and in hemorraghic shock and are facing a potentially life-threatening situation that requires immediate intervention.
(ii) Previous animal and human studies have been conducted, and suggest the potential for a direct benefit to individual patients who require large volume blood transfusion.
(iii) Plasma has been evaluated in patients who require large volume blood transfusion following injury and has been shown to provide a survival advantage. Plasma has also been independently shown to be associated with pulmonary complications but no higher risk of mortality. As discussed above, there are potential risks to subjects that may have not

been observed in previous trials. We contend that these risks are reasonable in light of the potential benefits outlined in this proposal and the current poor outcome for patients with hemorrhagic shock.

(4) The clinical investigation could not practicably be carried out without the waiver.

This study could not be conducted without the waiver of consent due to the need to administer the intervention in the prehospital setting en route to a definitive trauma center.

(5) The proposed investigational plan defines the length of the potential therapeutic window based on scientific evidence, and the investigator has committed to attempting to contact a legally authorized representative for each subject within that window of time and, if feasible, to asking the legally authorized representative contacted for consent within that window rather than proceeding without consent. The investigator will summarize efforts made to contact legally authorized representatives and make this information available to the IRB at the time of continuing review.

Patients in hemorrhagic shock following injury have been shown to develop progressive hypothermia, coagulopathy and acidosis leading to further recalcitrant hemorrhage and multisystem organ failure and death. The potential therapeutic window for addressing this process is during the initial resuscitation period, which occurs from arrival of the air medical transport provider on scene or at a referral hospital up until trauma center arrival. Since this is an immediately life-threatening situation, it will not always be possible to contact legal representatives at the time of study entry. We will make every effort to contact legal representatives after admission to the hospital to notify them that the patient was enrolled in a randomized trial. Research personnel will attempt to contact the subject's legal authorized representative as soon as feasible and a summary of these efforts will be documented in the patient's chart. If the subject becomes competent during the study period then he/she will be approached by research personnel for notification of enrollment.

(6) The IRB has reviewed and approved informed consent procedures and an informed consent document consistent with Sec. 50.25. These procedures and the informed consent document are to be used with subjects or their legally authorized representatives in situations where use of such procedures and documents is feasible. The IRB has reviewed and approved procedures and information to be used when providing an opportunity for a family member to object to a subject's participation in the clinical investigation consistent with paragraph (a)(7)(v) of this section.

All procedures and consent forms will be approved by the Institutional Review Board (IRB) of the study site prior to the onset of the trial.

(7) Additional protections of the rights and welfare of the subjects will be provided, including, at least:
i. Consultation (including, where appropriate, consultation carried out by the IRB) with representatives of the communities in which the clinical investigation will be conducted and from which the subjects will be drawn;
ii. Public disclosure to the communities in which the clinical investigation will be conducted and from which the subjects will be drawn, prior to initiation of the clinical investigation, of plans for the investigation and its risks and expected benefits;
iii. Public disclosure of sufficient information following completion of the clinical investigation to apprise the community and researchers of the study, including the demographic characteristics of the research population, and its results;
iv. Establishment of an independent data monitoring committee to exercise oversight of the clinical investigation; and
v. If obtaining informed consent is not feasible and a legally authorized representative is not reasonably available, the investigator has committed, if feasible, to attempting to contact within the therapeutic window the subject's family member who is not a legally authorized representative, and asking whether he or she objects to the subject's participation in the clinical investigation. The investigator will summarize efforts made to contact family members and make this information available to the IRB at the time of continuing review.

(i) Community consultation as outlined by the local IRB will be undertaken prior to IRB approval. Since the population eligible for enrollment includes all citizens in the study region it will not be possible to target any particular small group. Feedback from the community will be obtained by research personnel regarding any concerns they may have about potential enrollment. If requested, bracelets will be made available that could be worn by members of the community who

do not want to participate. Public notification and community consultation will be performed as directed by the local IRB and may include such methods as using random digit dialing telephone surveys of the proposed study community, targeted small group meetings or consultation with community leaders. Our institution has significant experience with community consultation and notification practices.
(ii) & (iii) Public disclosures will be performed both prior to study enrollment and at the completion of the study in the form of multimedia press releases organized by the investigators. These will include plans for the study including potential risks and benefits and a summary of the results of the study upon completion. In the event that the press releases are not widely circulated, advertisements will also be placed in local papers describing the study.
(iv) The Data Safety Monitoring Board will function as an independent data monitoring committee who will exercise oversight of the study.

(v) We expect that all patients who meet the enrollment criteria will be unconscious or in critical state that does not allow appropriate consent to occur. Any delay in medical care that would be required for the care provider to attempt to obtain consent from the patient’s legal guardian would be life threatening. Thus it will not be feasible to attempt to obtain informed consent during the initial therapeutic window. Requiring consent to review a hospital chart to determine the presence or absence of serious adverse events is likely to be associated with a biased estimate of the safety and efficacy of the intervention. Therefore we will use exception from consent for emergency research which includes public notification, community consultation, patient notification of enrollment, and provision of an opportunity to opt out from ongoing participation.

Clinical Protocol Appendix 2

Proposed Community Consultation and Public Disclosure Plan PAMPer Trial

I. Community Consultation

A. City of Pittsburgh
1. Pittsburgh Human Relations Committee
B. Website
Information about the current PAMPer Trial will be posted on a website which has been developed for this purpose. Contact information will be provided for questions and comments. All multimedia material will have the following website listed: www.acutecareresearch.org
There will be information on how to get more information about the trial and how to obtain on “opt out bracelet” if desired.
C. Surveys
Surveys will be placed in the Trauma Service outpatient clinic. They will also include the web address and contact information.

II. Public disclosure

A. Multi-Media
1. The UPMC Media Office will issue a press release describing the upcoming study and locations of public forums.
B. Notifications will be posted on our local Pittsburgh Authority public transportation buses. The website address will be posted. Contact information will be provided for questions and comments. This will include information regarding how to obtain an opt-out bracelet. This has been the most effective means of getting feedback in our area.

C. Opt out bracelets will be made available upon request. They will be PINK and state “NO PANPER”.

Clinical Protocol Appendix 3

Telephone Conversation Tracker for LAR Calls

Date	Start time	End time	Name	Phone Number	Subject ID	Notes	Action Items	Follow-up Needed? Y/N

Telephone Conversation Tracker for Outgoing Calls

Date	Start time	End time	Name	Phone Number	Subject	Notes	Action Items	Follow-up Needed? Y/N

Clinical Protocol Appendix 4

CENTER FOR EMERGENCY MEDICINE OF WESTERN PENNSYLVANIA,INC.
STAT MEDEVAC

Policy Number: 220
Effective Date: January 1, 2012
CAMTS Reference: None

Subject: Blood Product Maintenance

Policy:

1. Base site blood products will be inspected daily for adequate temperature maintenance in the blood product storage refrigerator. Daily temperature checks will be documented in conjunction with the weekly circular graph temperature recording. Blood products will also be properly signed out of the blood refrigerator when indicated for missions and maintained at proper temperature until transfused or returned to the refrigerator.
2. Inventory/Expiration Tracking Log
   a. All PRBC units must be logged on this sheet when received from the designated distribution point:
      1. STAT MedEvac 1 – Washington Hospital Transfusion Services Lab
      2. STAT MedEvac 2 -Johnstown American Red Cross
      3. STAT MedEvac 3 – UPMC Passavant Cranberry Blood Bank
      4. STAT MedEvac 4– UPMC McKeesport Transfusion Services Lab
      5. STAT MedEvac 5– Uniontown Hospital Blood Bank
      6. STAT MedEvac 6– Clarion Hospital Blood Bank
      7. STAT MedEvac 7– UPMC Horizon Transfusion Services Lab
      8. STAT MedEvac 8– UPMC Passavant Cranberry
      9. STAT MedEvac 9– American Red Cross
      10. STAT MedEvac 10- John Hopkins Hospital
      11. STAT MedEvac 11 -Altoona Hospital Blood Bank
      12. STAT MedEvac 13- Baltimore American Red Cross
      13. STAT MedEvac 14- UPMC Passavant Cranberry Blood Bank
      14. STAT MedEvac 15- Washington Hospital Transfusion Services Lab
      15. STAT MedEvac 16- UPMC Passavant Cranberry Blood Bank
      16. STAT MedEvac 17- UPMC Hamot
      17. STAT MedEvac 18- Children's National Medical Center Blood Bank

   b. Record the following information:
      1. Unit Number
      2. Date placed in service
      3. Date unit will expire
      4. Date unit is to be returned (minimum of 10 days prior to expiration date on blood products)
      5. Initials of person confirming ABO type and placing units into service.
      6. Disposition of units (transfused, wasted, or returned)
      7. Location: - Note the receiving facility where patient receiving PRBCs transfusion was admitted.
      8. Paperwork: Check that all appropriate paperwork is completed.
      9. Comments: As needed, and list flight number associated with blood product transfusion.
      10. Initials of person completing log regarding unit disposition.

Original Policy Date: June 20, 1994
Revision Date: June 25, 1998; June 2011; February 2003; May 2004; April 2007; December 2011

CENTER FOR EMERGENCY MEDICINE OF WESTERN PENNSYLVANIA,INC.
STAT MEDEVAC

Policy Number: 220
Effective Date: January 1, 2012
CAMTS Reference: None

3. Daily Checks / Shift Responsibilities: In order to prevent any problems with the recording chart to potentially go undetected for as long as 24 hours, check the chart midway through the shift for accurate documentation of current day and time every shift. Any problems should be corrected immediately and documented on the recording chart.
   a. Temperature and Visual Inspection to be completed on every shift recording the following information on the Temperature
   Check and Visual Inspection Sheet:
   1. Date of inspection
   2. Temperatures as indicated
      a. Make sure temperature reading on recording chart corresponds with current day, time.
      b. Make sure stylus is making contact with recording chart.
      c. Notify Base Site Manager of any problems, i.e.: possibility of contamination, temperature not maintained between 1o C to 6° C, inability to get recording chart to function properly.
   3. Refrigerator graph: Confirm accurate documentation of current day and time on the circular graph. Internal refrigerator temperatures and chart temperatures must both be within acceptable range (1-6° C) and agree with each other: t1°
      a. Any gaps or fluctuations in temperature on recording chart <u>must</u> have explanation documented on chart followed by your initials.
      b. A copy of all temperature documentation will be sent to the appropriate blood bank by the Base Site Coordinator or base representative.
   4. Contamination: Check each unit of blood for contamination and expiration date- i.e., if the red cell mass appears purple; if there is a zone of hemolysis: visible clots; if the plasma is murky; if plasma has a purple, brown, or red discoloration; if there are signs of leakage or inadequate sealing.
      a. PRBC units that are questionable must be quarantined and recorded as such in the comments section.
      b. Notify the appropriate blood bank immediately of quarantined blood.
      c. Exchange quarantined units for replacement units as soon as possible.
   5. Confirm that blood is "0" negative or positive.
   6. Confirm that blood is not due to expire by checking the inventory and expiration tracking log. If blood is to be returned, follow blood product return/transfer procedure.
   7. Confirm ice or commercial ice packs are available for transport.
   8. Comments as needed
   9. Initials of person doing inspection.
   b. Ensure refrigerator is clean and in working order.

Original Policy Date: June 20, 1994Revision Date: June 25, 1998; June 2011; February 2003; May 2004; April 2007; December 2011

CENTER FOR EMERGENCY MEDICINE OF WESTERN PENNSYLVANIA,INC.
STAT MEDEVAC

Policy Number: 220
Effective Date: January 1, 2012
CAMTS Reference: None

4. Weekly Recording Chart Change
   a. Every Monday the circular graph recording chart on top panel of the blood product storage refrigerator must be changed.
      Instructions to remove graph:
      1. Open the latch and door.
      2. Push the number "3" button on the interior panel to reposition the stylus.
      3. Unscrew center nut on graph.
      4. Remove graph paper.
      5. Write in the current date, in the appropriate space, in which the circular graph paper was removed and then initial (the graph will have been stamped when the new circular graph paper was placed).
      6. Examine the circular graph and ensure that any fluctuations in temperature on the graph or any corrective action to correct fluctuations in temperature are explained on the graph and initialed.
      7. Place completed circular graph in the Base Site Manager's or base representative's mail box. All graphs must be kept for a minimum of 5 years.
      8. Utilizing the commercial stamp mark the new circular graph along the outer edge of the paper and note in pen the Unit (listed as GEM), Location (listed as STAT X), Date in and initials of the person completing the form.
      9. Insert new graph into the receptacle and replace central nut.
      10. Push the number "3" button on the interior panel to return the stylus to the recording position.
      11. Ensure that the stylus is touching graph at the appropriate spot for current day and time.
      12. Close door and engage latch.
      13. Any problems existing after trouble shooting proves unsuccessful should be reported to the Base Site Manager.
5. Blood tracking log
   a. Columns 1-5 must be filled out when removing blood products from the refrigerator
      1. Date: Current date entered.
      2. Unit number: List all units available.
      3. Issued Time and Temperature: Current time and temperature when blood is being removed.
      4. Visual inspection: Check appearance, type, and expiration date to ensure it is correct and it is not expired. Record as satisfactory or unsatisfactory. Any units that appear unsatisfactory are not to be taken out on a mission but immediately placed out of service
      5. Initials: Your initials.
   b. Columns 6-10 must be filled out as indicated when returning blood products to the refrigerator.
      1. Disposition: If no units are transfused, list as returned. If units are transfused, indicate the specific units transfused and receiving hospital of patient transfer. If units are to be quarantined, indicate the specific units quarantined.
      2. Returned time: Time blood returned to blood product refrigerator.

3. Visual Inspection: Check appearance for contamination, clots, discoloration, etc. List as satisfactory or unsatisfactory.
4. Initials: Your initials.
5. Comments: Insert flight number and patient name if blood product was transfused.

7. Packing blood products for a mission
   a. Blood products are to be taken on every mission.
   b. Each unit should be in the plastic blood product bags
   c. Place in the insulated cooler with commercial ice packs or ice and an appropriate thermometer. The units should be "sandwiched" between the ice using appropriate barriers to prevent the units from coming in direct contact with the ice.
   d. If blood is not transfused, return it to the blood product refrigerator upon returning to base. Fill out remaining Columns 6-10 of Blood Tracking Log
8. Administration of blood products is to be carried out in strict accordance with STAT MedEvac Critical Care Protocols.
9. Documentation of transfusion
   a. When a unit is transfused during a mission make sure the appropriate information is relayed to the receiving facility including type and unit number.
   b. Fill out appropriate blood bank forms for transfused products per Central Blood Bank / American Red Cross instructions.
   c. Upon return to the base, fill out the Blood Tracking Log for Columns 6-10 as instructed.
10. Replacement of transfused blood products
    a. Notify appropriate blood bank that you have transfused blood and specify the number of replacements needed.
       i. Notify STAT Com and the Medical Director on call of any delay in receiving replacement units of blood and document the delay via special report.
11. Transfusion Complications
    a. Notify the receiving facility of the patient's signs and symptoms immediately upon arrival.
    b. Upon returning to base, an Adverse Reaction special report should be completed.
11. Blood refrigerator alarms
    a. Monthly Check- High and low temperature alarms to be checked on the first of every month.
       i. Remove blood from refrigerator and place on ice in cooler.
       ii. Remove probe from glycerol solution and place the probe on ice. Temperature of probe will register below 1c C within several minutes to activate alarm.
       iii. The designated operator should call to advise of the Alarm. Activate the silence button on the blood product storage refrigerator after receiving phone call.
       iv. Then place probe in tepid water to test high temperature alarm (>6°).
       v. Again, the designated operator should call to confirm alarm activation. Activate the silence button on the blood product storage refrigerator after receiving phone call.
       vi. Initial and note "Alarm Test" on Temperature Graph.

CENTER FOR EMERGENCY MEDICINE OF WESTERN PENNSYLVANIA,INC.
STAT MEDEVAC

Policy Number: 220
Effective Date: January 1, 2012
CAMTS Reference: None

vii. Document "Alarms Checked/Operational or Non- Operational" in comment section of Daily Temperature Checks and Visual Inspection Logs.

viii. If alarms fail or no phone call is received from the designated operator keep blood on ice in cooler and notify the Base Site Manager immediately for guidance.

c. Alarm Activation

i. The blood bank refrigerator will alarm any time the temperature in it rises above 6°C or below 1 0C or electrical power is shut *off* to the refrigerator.

ii. At the same time, the remote alarm will be activated at the Communications Specialist's switch board, the Communications Specialist will notify you by phone when the alarm activates.

iii. When alarm goes *off,* try to find any obvious causes, i.e., door is open to refrigerator, refrigerator is unplugged, circuit breaker is *off,* or circulating fan is not working.

iv. If cause cannot be found or corrected, removed blood and place it on ice in the cooler.

v. Notify the Base Site Manager immediately of problem. The Base Site Manager will instruct you as to what to do with the units of blood.

12. Quarterly Temperature Monitoring

a. Every quarter (March, June, September, and December) a verification of temperature maintenance of blood during emergency flights must be performed.

b. The following steps are to be performed when testing the temperatures:

1. Store the bottle in your refrigerator along with the units of blood until needed.
2. When packing units for an emergency, record the thermometer reading, time packed, date and initials on the card provided.
3. Place the bottle into the cooler along with the blood.
4. Assure that all the blood units and the bottle are covered with ice.
5. Upon return to base after flight, record the thermometer reading, time unpacked, date, and initials on the bottom of the card provided.
6. The acceptable range during transport is 1-10.

c. A record of all results will be maintained at the base

President: ______________________

Medical Director: ______________________

Original Policy Date: June 20, 1994
Revision Date: June 25, 1998; June 2011; February 2003; May 2004; April 2007; December 2011

Clinical Protocol Appendix 5

ITxM The Institute for Transfusion Medicine clinical services Pittsburgh, PA & Chicago, IL	Donor-Patient "Lookback"		Pg. 1 of 6
	Doc #: ITxM-CS-00795	Revision: 7	
	Department: Patient Transfusion Service		

DONOR-PATIENT "LOOKBACK"

APPROVALS

All Approvals are maintained and controlled via Document Control Systems' MC3 Portal™ Software. Please Refer to MC3 Portal™ for the current controlled revision and approval records.

SUMMARY OF THE MODIFICATIONS – See MASTERControl™ InfoCard Release Date

List a summary of the modifications below. Bullet outline is recommended.

- **Addition of UPMC East**
  UPMC EAST – Patients transfused after July 2, 2012 are in SafeTrace Tx. UPMC East has no transfusion records prior to this date.

Copyright© ITxM 2006. All rights reserved. May not be reproduced without permission. All hard copies should be checked against the current electronic version within MASTERControl™ prior to use and destroyed promptly thereafter. All hard copies are considered uncontrolled documents except when issued by Quality Assurance as an official hard copy for use in lieu of MASTERControl™.

PROCESS

SYSTEM

Investigation of Adverse Transfusion Effects, Information Management

CRITICAL CONTROL POINT

Documentation/Record Keeping, Supplier Qualification, Error/Accident Review, Internal Assessment, Process Improvement

PRINCIPLE

Regulatory agencies require notification of recipients of blood products from a donor who subsequently tests confirmed positive for HIV1,2, HCV or HTLV-I/II, or is at risk for transmitting Creutzfeldt-Jakob disease (CJD).

POLICY

I. IDENTIFICATION OF INFECTED DONORS

- PROSPECTIVE LOOKBACK - Units implicated in the lookback process are identified by Central Blood Bank or LifeSource and CTS or RCRL is notified in writing of the units and their shipping date. For HIV, HCV, and HBV lookback cases, CBB or LifeSource will recall all indate products within 3 days of a repeat reactive screening test and will notify the transfusion service in writing within 30 days of a positive Western blot for HIV or 45 days for a RIBA positive HCV test.
- RETROSPECTIVE LOOKBACK - Units implicated in the retrospective HCV lookback process are identified by CBB or LifeSource and the transfusion service is notified in writing of the units and their shipping date within 6 months of the September 23, 1998 publication of the FDA guidance document.
- CENTRALIZED TRANSFUSION SERVICE RECIPIENT IDENTIFICATION - The transfusion service must identify the recipient of any of the implicated units. The method of recipient tracing varies with the date of transfusion and the hospital.

PUH/E&E/CHP - After May 21, 1999 - SafeTrace Tx contains all units receipts received.
Between mid-October 1988 and May 20, 1999 - the PTS computer system or microfiche contains all units recipients received.
Prior to PTS computer (1988) - unit inventory cards (3 x 5) were used to record each unit, to whom it was issued and the hospital. Cards are stored in boxes stored in a warehouse at National Business Records Management (NBRM). There are cards dating back at least to 1977. (see Notes for retrieving cards)

MUH - Patients transfused after May 11, 1991 are in the PTS system. MUH has no transfusion records prior to this date.

ALLEGHENY GENERAL HOSPITAL - Patients transfused after May 21, 1999 are in SafeTrace Tx. Patients transfused between August 20, 1994 and May 20, 1999 are in the PTS system. Patients transfused prior to this date must be retrieved by AGH staff. From September 17, 1988 to August 19, 1994 records are at AGH Information Systems (SunQuest System). From March 14, 1979 to September 16, 1988, records are in log books stored at AGH Stat lab. Any additional records are stored at Iron Mountain. A request for the required records should be sent to the medical director of the department of pathology and the LIS manager.

Copyright© ITxM 2006. All rights reserved. May not be reproduced without permission. All hard copies should be checked against the current electronic version within MASTERControl™ prior to use and destroyed promptly thereafter. All hard copies are considered uncontrolled documents except when issued by Quality Assurance as an official hard copy for use in lieu of MASTERControl™.

ITxM The Institute for Transfusion Medicine clinical services Pittsburgh, PA & Chicago, IL	**Donor-Patient "Lookback"**	Pg. 3 of 6
	Doc#: ITxM-CS-00795 | Revision: 7	
	Department: Patient Transfusion Service	

SHADYSIDE HOSPITAL- Patients transfused after May 21, 1999 are in SafeTrace Tx. Patients transfused between October 7, 1994 and May 20, 1999 are in the PTS system. Patients transfused on or prior to this date must be extracted by SSH staff. A request for the required records should be sent to the medical director of the department of pathology and the laborat01y administrative director.

WASHINGTON HOSPITAL- Patients transfused after May 21, 1999 are in SafeTrace Tx. Patients transfused between July 7, 1995 and May 20, 1999 are in the PTS system. Patients transfused prior to this date are in card files and will be retrieved by WH staff. A request for the required records should be sent to the medical director of the department of pathology and the lab manager.

MCKEESPORT HOSPITAL -Patients transfused after May 21, 1999 are in SafeTrace Tx. Patients transfused between March 2, 1997 and May 20, 1999 are in the PTS system. Patients transfused on or prior to this date are in card files and will be retrieved by McK staff. A request for the required records should be sent to the medical director of the department of pathology and the lab manager.

UPMC, SOUTH SIDE HOSPITAL- Patients transfused after May 21, 1999 are in SafeTrace Tx. Patients transfused between February 28, 1997 and May 20, 1999 are in the PTS system. Patients transfused on or prior to this date are in card files and will be retrieved by SOSH staff. A request for the required records should be sent to the medical director of the department of pathology and the lab manager.

COMMUNITY MEDICAL CENTER- Patients transfused on or after October 5, 1999 are in the SafeTrace Tx system. Patients transfused prior to this date are referred to the medical director/lab manager to obtain the required information.

UPMC, ST. MARGARETS -Patients transfused after July 31, 2000 are in the SafeTrace Tx system. Patients transfused on or prior to this date are referred to the medical director/lab manager to obtain the required information.

MAGEE HOSPITAL- Patients transfused after December 10, 2000 are in SafeTracc Tx. Patients transfused on or prior to this date are referred to the medical director/lab manager of the hospital.

WEST PENN HOSPITAL- Patients transfused after November 19, 2000 are in SafeTrace Tx. Patients transfused on or prior to this date are referred to the medical director/lab manager of the hospital.

LIFECARE HOSPITAL- Patients transfused after January 17, 2000 are in SafeTrace Tx. Patients transfused on or prior to this date arc referred to the medical director/lab manager of the hospital.

KINDRED HOSPITAL - Patients transfused after December 31, 1999 are in SafeTrace Tx. Patients transfused on or prior to this date are referred to the medical director/lab manager of the hospital.

RCRL CHICAGO- Patient's transfused after June 1, 2000 are in SafeTrace Tx. For patient's transfused prior to this date, LifcSource will contact the transfusing facility directly. UPMC-BRA_DDOCK HOSPITAL-Patients transfused after September 16, 2001 are in SafeTrace Tx. Patients transfused prior to this date are rcfcned to the lab manager of the hospital.

FORBES REGIONAL HOSPITAL-Patients transfused after March 24, 2002 arc in SafeTrace Tx. Patients transfused prior to this date arc referred to the lab manager of the hospital.

Copyright© ITxM 2006. All rights reserved. May not be reproduced without permission. All hard copies should be checked against the current electronic version within MASTERControl™ prior to use and destroyed promptly thereafter. All hard copies are considered uncontrolled documents except when issued by Quality Assurance as an official hard copy for use in lieu of MASTERControl™.

UPMC PASSAV ANT AND UPMC PASSAVANT CRANBERRY HOSPITAL- Patients transfused after October 31, 2004 are in SafeTrace Tx. Patients transfused prior to this date are referred to the lab manager of the hospital.

AGH-SUBURBAN CAMPUS- Patients transfused after March 15, 2006, are in SafeTrace Tx. Patients transfused prior to this date are referred to the lab manager of the hospital.

UPMC MERCY- Patients transfused after December 1, 2008 are in SafeTrace Tx. Patients transfused prior to this date are referred to the lab manager of the hospital.

LIFELINE- Patients transfused after December 1, 2009 are in SafeTrace Tx.

ADVANCED SURGICAL -Patients transfused after May 1 , 20110 are in SafeTrace Tx.

MEADVILLE MEDICAL CENTER - Patients transfused after March 15, 2012 are in SafeTrace Tx. Patients transfused prior to this date are referred to the lab manager of the hospital.

UPMC EAST- Patients transfused after July 2, 2012 are in SafeTrace Tx. UPMC East has no transfusion records prior to this date.

II. CONFIRMING THE PATIENT AND IDENTIFYING THE PHYSICIAN

Pittsburgh- For patients since October 1988, the SafeTrace Tx has the patient's name, hospital number, birth date and sometimes the physician. If the physician's name is not available in the computer, this must be obtained from the medical record. For transfusions in the pre-computer era, the transfusion, the recipient, and the physician are verified by review of the medical record or by the PTS patient card. Patient cards are stored in boxes stored in a warehouse at National Business Records Management (NBRM).

Chicago -Patients transfused after June I, 2000 are in SafeTrace Tx. If physician information is not in SafeTrace Tx, the transfusion entity will receive only the information available prior to this time.

III. RECIPIENTS PHYSICIAN NOTIFICATION OF POSSIBLE VIRAL INFECTION

The CTS or RCRL Chicago physician will send the patient's physician, the physician who ordered the blood product, or the transfusing entity a letter notifying him/her of the lookback. All correspondence is sent by certified mail or by UPS. For HIV, HCV and HBV lookback, the physician must promptly return an enclosed confirmation letter to the transfusion service indicating that they accept responsibility for patient notification. In the case of HIV and HCV, patient notification includes the need for HIV or HCV testing and counseling. If the transfusion service cannot locate the physician, does not receive the confirmatory documentation from the physician or the physician refuses to accept responsibility for notification, then the transfusion service is responsible for notifying the patient. This is done by the CTS physician at CTS hospitals. The FDA requires that the process of notification be completed within 8 weeks for HIV and 12 weeks for HCV (prospective) and 1 year for HCV (retrospective).

Copyright© ITxM 2006. All rights reserved. May not be reproduced without permission. All hard copies should be checked against the current electronic version within MASTERControl™ prior to use and destroyed promptly thereafter. All hard copies are considered uncontrolled documents except when issued by Quality Assurance as an official hard copy for use in lieu of MASTERControl™.

IV. RECORDS

- The following documents are maintained in the look back file:
  - -Notification letter from CBB or LifeSource (HIV, HCV, HBV, HTLV-I, CJD)
  - -Copy of certified letter notifying recipient's physician (HIV, HCV, HBV, HTLV-1, CJD)
  - - Documentation indicating physician's acceptance of responsibility for patient notification (HIV, HCV only)
  - - Certified mail receipts
  - -Documentation of patient notification if performed by the transfusion service medical director. Three attempts will be made to notify the patient by letter for HCV or HIV lookback. If the first attempt is returned because the address is not valid, no further letters will be sent.
- Documentation of notification of the patient by his/her physician is maintained in the patient's medical record.
- Lookback records arc maintained at CTS.

PROCEDURE NOTES

1. STORED INVENTORY CARDS AND PATIENT RECORD CARDS FOR UPMC/CHP
The inventory-card boxes are listed, along with other stored PTS records, on a computer printout from NBRM labeled ("CBB- LAB PTS"). Photocopies of this computer printout are held by Central Records, Donor Counseling, and the Associate Medical Director. Each box is listed according to the first and last card (e.g., RIOOOO-Rl4000), and there are often more than one letter series in the same box. Some box records have the year; most do not. Some box numbers have a very broad span; often this is because frozen RBCs from years before were used and put into the current box with the same letter series. This means that there is usually more than one box on the list which could contain a given card. The end number of the box is closer to most of the cards in it.
2. POOLED COMPONENTS
Pools are given unique unit numbers in the computer. In SafeTrace Tx, the unique pool number can be found in the additional information tab of the component profile of the original unit. This pool number can then be queried to get final disposition information. In the PTS computer system, the blood unit inquiry for the original unit number will give P-number, and then this can be entered to yield the patient. Pre-computer, when a platelet was pooled, the P-number was put on the platelet's original inventory card and a new P-number inventory card was made. The P- series card has the recipient's name, so if only the original unit number is given, both cards must be sought sequentially for pre-1984 searches. After the BOS mainframe computer system began in 1984, pools in PTS were entered. Donor Counseling will provide the P-number or individual unit IDs from the mainframe.
3. OBTAINING INVENTORY CARDS/PATIENT RECORD CARDS
The Quality Department secretary provides forms for requesting stored boxes from NBRM.
After identifying the boxes needed from the computer list above, the request forms are completed and sent to the Quality Department secretary. They arrange for the boxes to be picked up by CBB drivers and brought to the requester in a few days.

REFERENCES

Federal Register Vol61, No. 175 September 9, 1996 page 47423-34.

Copyright© ITxM 2006. All rights reserved. May not be reproduced without permission. All hard copies should be checked against the current electronic version within MASTERControl™ prior to use and destroyed promptly thereafter. All hard copies are considered uncontrolled documents except when issued by Quality Assurance as an official hard copy for use in lieu of MASTERControl™.

<u>AUTHOR</u>
Darrell J. Triulzi, M.D., and Linda F. Hahn, MPM, MT(ASCP)SBB

Copyright© ITxM 2006. All rights reserved. May not be reproduced without permission. All hard copies should be checked against the current electronic version within MASTERControl™ prior to use and destroyed promptly thereafter. All hard copies are considered uncontrolled documents except when issued by Quality Assurance as an official hard copy for use in lieu of MASTERControl™.

Clinical Protocol Appendix 6

DSMB Charter

Prehospital Air Medical Plasma (PAMPer) trial

October 24, 2013

Version 5.0

Data Safety Monitoring Board (DSMB) Overview

Trial Description and Study Design

- Trial name: Prehospital Air Medical Plasma (PAMPer) trial
- Principal investigator (PI): Jason Sperry, MD, MPH
- Funding agency: Department of Defense
- Trial design: Multi-center, prospective, randomized, open label interventional trial.
- Phase: III
- Number of patients: 550
- Number of sites: 6

DSMB Description

- This DSMB will be coordinated by the PI, Jason Sperry, MD, MPH.
- This DSMB will be independent of the investigators, funding agency, regulatory agencies, and institutional review boards.
- This charter will be approved by its DSMB members as attested to by signature of the chairperson.

DSMB Membership

- Members will disclose conflicts of interest and will be cleared of significant conflicts of interest and potential conflicts of interest in accordance with provisions in this charter.
- DSMB members will sign confidentiality agreements covering DSMB activities.
- Composition of membership will be researchers with the following expertise: emergency medicine, surgery (trauma/critical medicine), biostatistics and a bioethicist.
- Remuneration will be provided any expenses related to DSMB activities.

Reporting

- Unblinded data to be reviewed by the DSMB will be provided by an independent statistician. Issues and recommendations identified by the DSMB will be provided to the principal investigator by the DSMB chairperson in accordance with this charter.
- Details of closed session deliberations (e.g., minutes) will be considered privileged and not subject to disclosure except as required by law.

Introduction

The purpose of this charter is to define the roles and responsibilities of the DSMB, delineate qualifications of the membership, describe the purpose and timing of meetings, provide the procedures for ensuring confidentiality and proper communication, and outline the content of the reports.

The DSMB will function in accordance with the principles of the following documents: FDA document "Guidance for Clinical Trial Sponsors: On the Establishment and Operation of Clinical Trial Data Monitoring Committees".

Study Overview/Summary

Objective/Hypothesis: The primary hypothesis will be that prehospital infusion of plasma during air medical transport in patients with hemorrhagic shock will reduce overall blood transfusion requirements in the first 24 hours post injury. The secondary hypotheses include that prehospital infusion of plasma will reduce the incidence of mortality, multiple organ failure, nosocomial infection, and acute lung injury; reduce or prevent the early coagulopathy as demonstrated by improvements in presenting coagulation and thromboelastography parameters; and reduce the early inflammatory cytokine response, thrombomodulin and increase protein C levels.

Specific Aims:
Aim#1: Determine whether prehospital infusion of plasma as compared to standard air medical care results in a reduction in 24 hour blood transfusion requirements.
Aim#2: Determine whether prehospital infusion of plasma as compared to standard air medical care results in a reduction in the incidence of in-hospital mortality, multiple organ failure, nosocomial infection, acute lung injury and 24 hour blood component transfusion and resuscitation requirements.
Aim#3: Determine whether prehospital infusion of plasma as compared to standard air medical care results in an improvement in the acute coagulopathy of trauma, lower early IL-6 cytokine levels, reduced thrombomodulin and increased protein C levels.

Study Design: Multi-center, prospective, randomized, open label, interventional trial over 4yrs focusing on patients with concern for hemorrhagic shock being transported via air ambulance to definitive trauma care.

Population: Blunt or penetrating injured patients with hemorrhagic shock being transported via air medical services from the scene of injury or from referring hospital to a definitive care trauma center participating in the trial.

Inclusion Criteria:
1. Blunt or penetrating injured patients being transported from scene or referral hospital to PAMPer site
AND
2. Systolic blood pressure below 90mmHg AND tachycardia>108 at scene, or at outside hospital or during transport
OR
3. Systolic blood pressure below 70mmHg at scene, or outside hospital or during transport

Exclusion Criteria:
1. Wearing NO PAMP opt –out bracelet
2. Age > 90 or < 18 years of age
3. Inability to obtain intravenous or interosseous access
4. Isolated fall from standing injury mechanism
5 Documented cervical cord injury with motor deficit
6 Known prisoner or known pregnancy
7. Traumatic arrest with > 5 minutes of CPR without return of vital signs
8. Brain matter exposed or penetrating brain injury (GSW)
9. Isolated drowning or hanging victims
10. Isolated burns > estimated 20% total body surface area
11. Referral Hospital In-patient admission
12. Objection to study voiced by subject or family member at scene

Intervention: Eligible patients will be randomized to receive 2 units of AB thawed plasma, not older than 5 days, vs. standard air medical care. To minimize waste of AB plasma or low titer anti-B A plasma, local blood bank affiliates in coordination with each participating center will exchange unused, ≤ 5 day-old AB thawed plasma allowing its subsequent clinical use.

Randomization scheme: Respective air medical services will be randomly divided into 2 groups by either air base or helicopter, depending on each service's organizational characteristics. These groups will then be 4-month, block randomized to either AB plasma or low titer anti-B A plasma or standard care.

Roles and Responsibilities

DSMB Roles and Responsibilities

This DSMB will

- Meet periodically (see DSMB Meetings) to review aggregate and individual subject data related to safety, data integrity and overall conduct of the trial.
- Review specific interim analyses for efficacy (see Study Review Criteria/Stopping Rules and Guidelines).
- Provide recommendations to continue or terminate the trial depending upon these analyses.
- Communicate other recommendations or concerns as appropriate.
- Operate according to the procedures described in this charter and all procedures of the DSMB.
- Follow conflict of interest guidelines as detailed below (see DSMB Membership).
- Comply with confidentiality procedures as described below (see Confidentiality).
- Maintain documentation and records of all activities as described below (see DSMB Meetings, DSMB Reports).

Principal Investigator (or Designees) Roles and Responsibilities

The PI will directly or through delegation:

- Assure the proper conduct of the study.
- Assure collection of accurate and timely data (monitoring and data management).
- The PI will designate an independent statistician to compile and report SAEs to the DSMB.
- Promptly report potential safety concern(s) to the DSMB.
- Prepare summary reports of relevant data for the DSMB. (This may include analyses not otherwise outlined in this charter based upon findings.)
- Provide an independent facilitator for presentation of results during DSMB meetings if requested by the DSMB.
- Communicate with regulatory authorities, IRB, and investigators, in a manner that maintains integrity of the data, as necessary. (This communication is not the responsibility of the DSMB.)
- Provide funding for the study and DSMB.
- PI will not attend the closed session of the DSMB Meeting.

DSMB Membership

The DSMB will consist of at least 4 members. The DSMB members have been selected by the PI in consultation with the investigators.

As characteristic qualifications, members will:

- Work professionally and meet qualifications for their respective professions.
- Comply with accepted practices of their respective professions.
- Comply with the conflict of interest policies specified by the standard operating procedures (SOPs) of the PI to ensure that members do not have serious scientific, financial, personal, or other conflicts of interest related to the conduct, outcome, or impact of the study according to the guidelines specified below (e.g., engaged in any simultaneously occurring competitive trials in any role that could pose a conflict of interest for this study).
- Be independent from the PI, IRB, regulatory agencies, principal investigator, co-principal or sub-principal investigator, site investigator, site sub-investigator, clinical care of the study subjects, or any other capacity related to trial operations.
- Not be on the list of Notice of Initiation of Disqualification Proceedings and Opportunity to Explain (NIDPOE) (http://www.fda.gov/foi/nidpoe/default.html) and/or debarred list of investigators (http://www.fda.gov/ora/compliance_ref/debar).

Although each DSMB member will be expected to serve for the duration of the trial, in the unlikely event that a member is unable to continue participation, the reason will be documented and a replacement will be selected by the PI.

The DSMB will follow conflict of interest guidelines referenced by the Department of Health and Human Services, Financial Relationships and Interests in Research Involving Human Subjects: Guidance for Human Subject Protection (http://www.hhs.gov/ohrp/humansubjects/finreltnn/fguid.pdf). DSMB members will sign a non-conflict of interest statement

in regard to this study which will be on file with the PI. As determined by the PI, conflicts of interest and/or potential conflicts of interest (as determined by SOPs) will be reduced to the greatest extent that is consistent with assembling a highly competent DSMB. Any questions or concerns that arise regarding conflicts of interest will be addressed by the DSMB chairperson with input from other DSMB members and PI as necessary.

DSMB Meetings

Projected Schedule of Meetings

An initial meeting of the DSMB will be held prior to any subject enrollment in the study in order for the members to review the charter, to form an understanding of the protocol and definitions being used, to establish a meeting schedule, and to review the study modification and/or termination guidelines. Subsequent interim and final review meetings will be held to review and discuss interim and final study data (adverse events, protocol deviations, enrollment summary and tables for overall primary and secondary endpoints). Frequency of meetings will be every six months, unless the board determines otherwise.

Meeting Format

DSMB meetings will generally be conducted by teleconference and coordinated by the PI. A quorum, defined as 2 out of 4 members will be required to hold a DSMB meeting. Critical decisions of the DSMB should be made by unanimous vote. However, if this is not possible, majority vote will decide.

Open and Closed Sessions

The open session may be attended by the PI and study investigators or their designees. Data presented in the open session may include enrollment data, individual adverse event data, baseline characteristics, overall data accuracy and compliance data or issues, and other administrative data. Minutes of the open session will be recorded by the Chair of the DSMB. Minutes will be finalized upon signature of the chairperson and maintained by the DSMB in accordance with applicable statutory regulation.

The closed session will be restricted to the DSMB members. A facilitator or recorder may be requested by the DSMB. Data which may compromise the integrity of the study (e.g., comparative data) will be analyzed and discussed only in the closed session. The minutes of the closed session will be recorded by the DSMB Chair. Minutes from the closed session will be recorded separately from the minutes of the open session and stored securely by the DSMB Chair. Closed session minutes, finalized by signature of the chairperson, will be maintained in confidence and retained until discarded in accordance with applicable statutory regulation.

Following each meeting, a report separate from the minutes of the open and closed sessions will be sent to the PI describing the DSMB recommendations and rationale for such (see DSMB Communication of Findings and Recommendations).

Study Review Criteria/Stopping Rules and Guidelines

Guidance for the conduct of safety and efficacy analyses, and guidelines / stopping rules will be established prior to the DSMB's first evaluation of data.

Safety Analyses

The primary safety endpoint is mortality as observed during interim analysis. In addition to the primary safety endpoint, the DSMB will monitor the following adverse events:

1. ARDS (adult respiratory distress syndrome)
2. TRALI (transfusion related acute lung injury)
3. MOF (- multiple organ failure)
4. Transfusion reactions
5. Surgical interventions
6. Complications due to specific injuries
7. Other major medical or surgical complications are commonly observed in these patients

Stopping Guidelines / Stopping Rules: Safety

Termination or modification may be recommended for any perceived safety concern based on clinical judgment, including but not limited to a higher than anticipated rate for any component of the primary endpoint resulting in adverse events, or unexpected SAEs.

Efficacy Analyses

The primary outcome variable 30 day mortality will be utilized to access for efficacy of the trial. Accessing this primary outcome variable at each interim analysis will allow early termination of the trial for either lack of efficacy or excessive efficacy or benefit provided by early prehospital plasma.

Adaptive Protocol Modification
There is no planned sample size re-estimation; however if the DSMB reveals a need, the sample size calculation can be re-evaluated.

Consideration of External Data
The DSMB will also consider data from other studies or external sources during its deliberations, if available, as these results may have a profound impact on the status of the patients and design of the current study.

DSMB Reports

Monitoring for Safety
The primary charge of the DSMB is to monitor the study for patient safety. Formal DSMB safety reviews will occur as specified above (see Study Review Criteria/Stopping Rules and Guidelines).

Monitoring for Efficacy
The DSMB will monitor efficacy outcomes to determine relative risk/benefit, futility, or for early termination due to overwhelming efficacy. Interim analyses efficacy reports sent to the DSMB will occur as specified above (see Study Review Criteria/Stopping Rules and Guidelines).

Monitoring for Study Conduct
The DSMB will review data related to study conduct. Data to be reviewed and listed in the DSMB reports includes: enrollment rates over time, time from last patient enrolled to date of report (indication of delay between treatment or follow-up and reporting), summary of protocol violations, and completeness of treatment and follow-up visit data.

Data Flow for Adverse Events
The DSMB will carefully monitor adverse events periodically throughout the duration of the study. This process will be dynamic to include quarterly reviews of all reported SAEs by the DSMB chairperson. The investigators will be expected to report Serious Adverse Events (SAEs) to the PI within 24 hours of knowledge of the event. The PI will then report it to the DSMB within 7 days.

Preparation of Reports to the DSMB
The University of Pittsburgh Coordinating Center will generate unblinded data for DSMB review. The PI will prepare and distribute reports to the DSMB electronically approximately 7 days prior to the date of each DSMB meeting.

In order to provide the maximum amount of information to the DSMB, the analyses will employ the most recent data (recognizing limitations thereof) available at the time of the analysis. Requests for additional data by the DSMB members will be made to the DSMB chairperson or his or her designee, who will be responsible for communicating the request with the PI.

The DSMB will review the data and discuss the analyses during the closed portion of the scheduled meeting.

DSMB Communication of Findings and Recommendations
Following each meeting and within 7 days of the meeting, the chairperson will send findings and recommendations of the DSMB in writing to the PI.

These findings and recommendations can result from both the open and closed sessions of the DSMB. If these findings include serious and potentially consequential recommendations that require immediate action, the chairperson will also promptly notify the PI by phone and/or by email.

PI's Response to DSMB Findings and Recommendations
The PI and co-investigators will review and respond to the DSMB recommendations. The recommendations of the DSMB will not be legally binding but require professional consideration by the recipients. If the DSMB recommends continuation of the study without modification, no formal response will be required. However, if the recommendations request action, such as a recommendation for termination of the study or modification of the protocol, the DSMB will request that the PI provide a formal written response stating whether the recommendations will be followed and the plan for addressing the issues.

It is recognized that the PI may need to consult with regulatory agencies or other consultants before finalizing the response to the DSMB. Upon receipt, the DSMB will consider the PI response and will attempt to resolve relevant issues, resulting in a final decision. Appropriate caution will be necessary during this process to avoid compromising study

integrity or the ability of the PI to manage the study, should the study continue. The PI will agree to disseminate the final decision to the appropriate regulatory agencies, IRB, and investigators within an appropriate time.

In the unlikely event of irreconcilable differences, especially regarding study termination or other substantial study modifications, the DSMB may decide to discontinue monitoring the current study and disband. This decision will be communicated to the PI, FDA, and IRBs.

Public disclosure of the PI's final decision or DSMB recommendations will be at the discretion of the PI or their designee. The DSMB will not make any public announcements either as a group or individually.

DSMB Closeout

This study may be terminated under a variety of circumstances including, but not limited to, termination for overwhelming effectiveness, futility, or safety issues per protocol or DSMB monitoring guidelines. Responsibilities of the DSMB with regard to closeout will be to review the final study report to ensure study integrity. The DSMB may recommend continuing action items to the PI based upon the final review.

Confidentiality

All data provided to the DSMB and all deliberations of the DSMB will be privileged and confidential. The DSMB will agree to use this information to accomplish the responsibilities of the DSMB and will not use it for other purposes without written consent from the study PI and co-investigators. No communication of the deliberations or recommendations of the DSMB, either written or oral, will occur except as required for the DSMB to fulfill its responsibilities. Individual DSMB members must not have direct communication regarding the study outside the DSMB (including, but not limited to the investigators, IRB, regulatory agencies, or PI) except as authorized by the DSMB.

Amendments to the DSMB Charter

This DSMB charter can be amended as needed during the course of the study. Information to be included as amendments will be any modifications or supplements to the reports prepared for the DSMB, as well as amendments to other information addressed in this charter. All amendments will be documented with sequential version numbers and revision dates, and will be recorded in the minutes of the DSMB meetings. Each revision will be reviewed and agreed upon by both the study PI and the DSMB. All versions of the charter will be archived in accordance with this document and maintained by the PI.

Clinical Protocol Appendix 7

Reporting Requirements

Reporting Requirements and Responsibilities of the Principal Investigator to the United States Army Medical Research and Materiel Command's (USAMRMC), Office of Research Protections (ORP), Human Research Protection Office (HRPO).

a. The protocol will not be initiated until written notification of approval of the research project is issued by the HRPO.

b. The Principal Investigator has a duty and responsibility to foster open and honest communication with research subjects. The USAMRMC strongly encourages the Principal Investigator to provide subjects with a copy of the research protocol, if requested, with proprietary and personal information redacted as needed.

c. The Principal Investigator must comply with the following minimum reporting requirements. Specific reporting requirements for the protocol will be included in the HRPO Approval Memorandum. Failure to comply could result in suspension of funding.

(1) Substantive modifications to the research protocol and any modifications that could potentially increase risk to subjects must be submitted to the HRPO for approval prior to implementation. The USAMRMC ORP HRPO defines a substantive modification as a change in Principal Investigator, change or addition of an institution, elimination or alteration of the consent process, change to the study population that has regulatory implications (e.g. adding children, adding active duty population, etc), significant change in study design (i.e. would prompt additional scientific review) or a change that could potentially increase risks to subjects.

(2) Any changes of the IRB used to review and approve the research will be promptly reported to the USAMRMC ORP HRPO.

(3) All unanticipated problems involving risk to subjects or others must be promptly reported by telephone (301-619-2165), by email (HRPO@amedd.army.mil), or by facsimile (301-619-7803) to the HRPO. A complete written report will follow the initial notification. In addition to the methods above, the complete report can be sent to the U.S. Army Medical Research and Materiel Command, ATTN: MCMR-RP, 504 Scott Street, Fort Detrick, Maryland 21702-5012.

(4) Suspensions, clinical holds (voluntary or involuntary), or terminations of this research by the Institutional Review Board (IRB), the institution, the Sponsor, or regulatory agencies will be promptly reported to the USAMRMC ORP HRPO.

(5) A copy of the continuing review approval notification by the IRB of Record must be submitted to the HRPO as soon as possible after receipt. For greater than minimal risk research, a copy of the continuing review report approved by the IRB must also be provided. Please note that the HRPO also conducts random audits at the time of continuing review. Additional information and documentation may be requested at that time.

(6) The final study report, including any acknowledgement documentation and supporting documents, must be submitted to the HRPO when available.

(7) The knowledge of any pending compliance inspection/visit by the FDA, DHHS Office of Human Research Protections (OHRP), or other government agency concerning this research, the issuance of Inspection Reports, FDA Form 483, warning letters or actions taken by any regulatory agencies including legal or medical actions and any instances of serious or continuing noncompliance with the regulations or requirements, must be promptly reported to the HRPO.

Please Note: The USAMRMC ORP HRPO conducts site visits as part of its responsibility for compliance oversight. Accurate and complete study records must be maintained and made available to representatives of the USAMRMC as a part of their responsibility to protect human subjects in research. Research records must be stored in a confidential manner so as to protect the confidentiality of subject information.

For questions regarding the HRPO human research protocol review requirements email hrpo@amedd.army.mil or leave a voicemail at 301-619-2165 and a staff member will contact you.

Clinical Protocol Appendix 8

Harmonization Protocol for Prehospital Use of Plasma for Traumatic Hemorrhage Clinical Studies

Date: 22 October 2013

Point of Contact and Principal Investigator for Harmonization Protocol: Anthony E. Pusateri, PhD

1. Introduction

This harmonization protocol describes two phases for harmonizing three clinical studies that examine the prehospital use of plasma for traumatic hemorrhage. The primary harmonization plan is effective immediately and harmonizes the key study components that are critical to the primary unifying hypotheses and to the preplanned meta-analysis related to those primary unifying hypotheses. The secondary harmonization plan will be developed later, and will address secondary hypotheses and exploratory analyses. In this document, the primary harmonization will be described in detail. The approach and components related the secondary harmonization plan will be identified but will not be discussed in detail.

Currently, there is great interest in the potential use of plasma as the initial resuscitation fluid for traumatic hemorrhage. Traditionally, initial resuscitation has included fluids such as crystalloids. Plasma has been used as part of transfusion during the in-hospital phase of care. Recent evidence suggests both that earlier transfusion and that a higher ratio of transfused plasma with respect to red cells improve outcomes. These findings suggest that earlier use of plasma may be beneficial in trauma patients; however, there are little clinical data on the use of plasma in the pre-hospital environment. The question of the utility of plasma in the prehospital environment is especially significant in combat casualty care because of the challenges of the battlefield that may result in unpredictable and often prolonged evacuation times. Therefore, the US DoD has sponsored three clinical trials to study the potential beneficial or negative effects of plasma in the prehospital setting.

The approach taken in the overarching research program was to fund three separate studies, as opposed to funding a single very large study for licensure. This approach was taken because of the limited information available on the prehospital use of plasma. The approach was designed to provide information on prehospital plasma use under the different conditions and approaches provided by three separate studies. To obtain the maximum amount of information possible, the three clinical studies will be harmonized to provide the most effective possible meta-analysis addressing the most important outcomes. Harmonization is not meant to significantly alter the objectives or success of any individual study. An additional reason for harmonization is to facilitate an interface with the NHLBI-DoD Trans-agency Consortium on Coagulopathy in Trauma (TACTIC).

2. Purpose of this document

This document is a Harmonization Protocol. It identifies the specific points of harmonization among the three separate clinical studies. This document also identifies the unifying hypotheses, approach to data integration, and the meta-analysis plan, as well as relevant coordination procedures. Specific details about clinical protocol procedures are included within each separate study protocol (Appendices 1-3). This harmonization protocol does not replace or negate any planned analyses described for each individual site, nor does it detract from the unique characteristics of each study. This document describes procedures and analyses that will bring together the three studies with the purpose of capitalizing on the increased statistical power made possible by combining selected, harmonized data and by conducting meta-analyses according to a pre-planned, statistically valid approach.

3. This document brings together three separate clinical studies.

a. Study Title: Control Of Major Bleeding After Trauma (COMBAT): A prospective, randomized Comparison of fresh frozen plasma versus standard crystalloid intravenous fluid as initial resuscitation fluid
Principal Investigator: Ernest E. Moore, M.D., Denver Health Medical Center, Denver, CO

b. Study Title: Prehospital Air Medical Plasma (PAMPer) Phase III Multicenter, Prospective, Randomized, Open-label, Interventional Trial
Principle Investigator: Jason L. Sperry MD, MPH, University of Pittsburgh Medical Center, Pittsburgh, PA

c. Study Title: Pre-Hospital Use of Plasma for Traumatic Hemorrhage – (PUPTH_Study)
Principle Investigator: Bruce D. Spiess, MD, Virginia Commonwealth University Medical School, Richmond, VA

The scientific and clinical backgrounds and rationales for each study are thoroughly reviewed in the individual clinical protocols (Appendices 1-3).

4. Unifying Hypotheses

The following hypotheses will be addressed by the combined study harmonization plan and meta-analysis.

Primary Outcome

1. Prehospital administration of 2 units of plasma will reduce mortality at 30 days after ED arrival

Secondary Outcomes

2. Prehospital administration of 2 units of plasma will reduce mortality

a. Prehospital administration of 2 units of plasma will reduce mortality at time of emergency department (ED) arrival

b. Prehospital administration of 2 units of plasma will reduce mortality at 24 hours after ED arrival

3. Prehospital administration of 2 units of plasma will reduce 24 hour transfusion requirements4. Prehospital administration of 2 units of plasma will improve standard coagulation parameters at the time of ED arrival

5. Prehospital administration of 2 units of plasma will improve clot viscoelastic properties (thromboelastograph (TEG) parameters) at the time of ED arrival

6. Prehospital administration of 2 units of plasma will improve hemodynamic parameters (systolic blood pressure (SBP) and heart rate (HR)) at the time of ED arrival

7. Prehospital administration of 2 units of plasma will improve cellular hematologic parameters (hematocrit, red cells, platelet count) at the time of ED arrival

8. Prehospital administration of 2 units of plasma will improve metabolic status (lactate, blood gases, pH, base deficit) at the time of ED arrival

9. Prehospital administration of 2 units of plasma will improve International Society on Thrombosis and Haemostasis Disseminated Intravascular Coagulation Score (ISTH DIC Score) at the time of ED arrival and at 24 hours after ED arrival

5. Clinical Protocol Harmonization Approach

This protocol harmonization will be conducted in two stages, Primary Harmonization and Secondary Harmonization. Primary Harmonization will be accomplished prior to the start of patient enrollment with the purpose to support the unifying hypotheses stated in this document. This will include such key aspects as experimental treatments and inclusion/exclusion criteria, among others. Secondary harmonization will be accomplished later and will include specific assay methodology and other aspects of the study. The approach to harmonization will be to attain agreement among site principal investigators and then to obtain local IRB, USAMRMC Human Use Review Office, Secretary of the Army, and FDA approval for any required protocol modifications. Ideally, all changes that require FDA and/or Secretary of the Army approval will be accomplished as part of primary harmonization. It is hoped that items harmonized during secondary harmonization will require only IRB notification or, at most, IRB concurrence for approval.

Primary harmonization will include the following aspects of each clinical study:

1. Experimental Treatment Groups

2. Inclusion and Exclusion Criteria

3. Timing of blood samples and identification of key parameters and assays

4. Adverse events

5. Methods to account for patient transport time

6. Enabling language and permissions for secondary harmonization.

Secondary Harmonization will include the following aspects of each clinical study:

1. Assay procedures and reagents

2. Blood sampling and handling procedures

3. Sample processing and storage procedures

4. Timing and number of blood samples (additional harmonization beyond that stated for primary harmonization)

5. Consolidation of procedures and laboratories to run assays

1. Experimental Treatment Groups

Across the three individual studies (Combined Study), the experimental treatment groups will be:

Control: Prehospital standard of care crystalloid resuscitation or fluid infusion

Treatment: Prehospital administration of 2 units of plasma

The individual study sites differ somewhat with respect to the specific plasma component and preparation procedures to be used (Table 1). These are dictated by local blood bank policy and it will not be possible to change these parameters. However, we believe that the procedures to be used for each individual study are similar enough to enable the overarching analyses described in the preplanned meta-analysis section. The volume of blood products administered will be recorded in units or volume. Randomization will be accomplished as described in each individual site protocol (Appendices1-3).

Table 1. Treatment Groups at Individual Sites

Parameter	Colorado (COMBAT)	Pittsburgh (PAMPer)	Virginia (PUPTH)
Blood Component	Type AB FP24 thawed plasma	Type AB thawed plasma	Type A thawed plasma
Handling Procedures	Plasma will be carried frozen and will be thawed in the ambulance using FDA approved microwave or other approved method	Thawed plasma (TP) will be carried as refrigerated thawed plasma. Thawed plasma will not be older than 5 days (post-thaw), and will be rotated every 5 days.	Thawed plasma will be carried as refrigerated thawed plasma in EMS supervisor vehicles.
How administered	Gravity feed with manual compression. TP will be administered by a paramedic or higher level care provider via a dedicated large bore line. If not randomized to TP, then standard crystalloid will be administered in the same manner. A limited amount of crystalloid may be administered prior to TP. The volume administered will be documented. Crystalloid will not be warmed in field.	Gravity feed with manual compression. TP will be administered by a paramedic or higher level care provider via a dedicated large bore line. If not randomized to TP, then standard crystalloid will be administered in the same manner. A limited amount of crystalloid may be administered prior to TP. The volume or units administered will be documented. Plasma and crystalloid will not be warmed in field.	Gravity feed with manual compression. TP will be administered by EMS supervisor via a dedicated large bore line. If not randomized to TP, then standard NS resuscitation will be administered in the same manner A limited amount of crystalloid may be administered prior to TP. The volume administered will be documented. Plasma and crystalloid will not be warmed in field.
Procedure	Patients randomized to the plasma group will receive 2 units of plasma before crystalloids. If the plasma is not ready and a patient needs fluids, Normal saline (NS) will be administered until plasma is ready. There	Patients that are randomized to the plasma group will receive 2 units of plasma prior to administration of any other fluids or blood components. . The volume or units of crystalloid administered	Patients randomized to the plasma group will receive 2 units of plasma before crystalloids. If the plasma is not ready and a patient needs fluids, NS will be administered until plasma is ready. There is no limit to

	is no limit to crystalloid volume. The volume of crystalloid administered and when it is administered (before or after plasma) will be documented for all patients in both treatment groups.	and when it is administered (before or after plasma) will be documented for all. patients in both treatment groups. The volume or units and timing of red cell administration will also be documented.	crystalloid volume. The volume of crystalloid administered and when it is administered (before or after plasma) will be documented for all patients in both treatment groups.
Control Group	Normal saline will be used for resuscitation. There will be no limit to total NS administered. The volume of crystalloid administered will be documented for each patient.	Crystalloid resuscitation will be performed using (NS or lactated Ringer's solution (LR)) as needed (no upper limit).<br><br>Those patients with persistent hypotension (SBP>90mmHg) with completion of the 2 units of plasma or initial crystalloid treatment will follow a goal directed prehospital crystalloid resuscitation standard operation procedure which includes crystalloid bolus infusion or uncrossmatched blood depending on the particular air medical service for patients who remain hypotensive after the plasma intervention.<br><br>The volume or units and timing of both crystalloid and red cells will be documented.	Normal saline will be used for resuscitation The volume of crystalloid administered will be documented for each patient.
Standard of care	Normal saline as needed (no upper	Crystalloid (NS ro LR) as needed (no upper limit). Some	Normal saline as needed (no upper

	limit).	participating sites also administer packed red cells during aero-medical transport.	limit).

2. Inclusion and Exclusion Criteria

Inclusion and exclusion criteria are compatible among the three individual studies. Inclusion and exclusion criteria are phrased differently among the three individual protocols (Appendices 1-3). There are differences in specific wording and details of the inclusion and exclusion criteria as written. These are summarized in Tables 2 and 3. For purposes of the combined harmonization protocol, simplified inclusion and exclusion criteria have been developed (Tables 4-5). We believe that these simplified inclusion and exclusion criteria reflect the primary features and intent identified in each protocol and describe, for the combined protocol, valid criteria that identify the harmonized patient population for purposes of the harmonized analysis and interpretation of the combined studies. Although there may remain slight differences in inclusion and exclusion criteria, it is expected that the number of enrolled patients that fall outside of the simplified, harmonized criteria will be so small as to not appreciably affect the projected power of the planned analyses through exclusion of these patients from the harmonized dataset. Exclusion would only be required for the primary and secondary unified hypotheses..

Table 2. Inclusion Criteria For Each Clinical Study

Criteria	COMBAT	PAMPer	PUPTH
Type of injury	Acutely injured trauma patients in severe, presumed hemorrhagic shock.	Blunt or penetrating injured patients with hemorrhagic shock	Blunt or penetrating trauma
Age	Age >/= 18 years	Age 18 to 90 years	Age>/=18 years
Gender	Either sex	Either sex	Either sex
Hemorrhagic Shock Status	Acutely injured, with presumed hemorrhagic shock from acute blood loss defined as SBP≤70 mmHg or SBP 71-90 mmHg with HR≥108 beats per minute.	Acutely injured, with presumed hemorrhagic shock from acute blood loss defined as SBP≤70 mmHg or SBP 71-90 mmHg with HR≥108 beats per minute.	BP systolic </=70 mmHg or BP systolic 70-90 mmHg with HR >/=108 BPM
			Major, ongoing hemorrhage, expected unstable vital signs

			consistent with above
Transport		Air medical transport to tertiary definitive care trauma center participating in the trial	
Consent			If lucid, able to consent (if feasible LAR/next of kin available and provides consent (abbreviated)), otherwise exception from informed consent

Table 3. Exclusion Criteria For Each Clinical Study

Criteria	COMBAT	PAMPer	PUPTH
Age	Age<18 years	Age >90 or <Age 18 years of age	Age <18 years
Not expected to survive	Unsalvageable injuries (defined as asystolic or CPR prior to randomization)		Not expected to survive transport to VCUMC
Head or CNS injury	Isolated gunshot wound to the head (a highly lethal injury that is not primarily due to blood loss)	Penetrating cranial injury	Penetrating head trauma
		Traumatic brain injury with brain matter exposed	
		Documented cervical cord injury with motor deficit	
Pregnancy	Visibly or verbally reported pregnant woman	Known pregnancy	Known/obvious pregnancy

Prisoner	Known prisoner	Known prisoner	Prisoner
Cardiac activity	Unsalvageable injuries (defined as asystolic or CPR prior to randomization)	Trauma arrest with >5 minutes of CPR without return of vital signs	Cardiac arrest or CPR prior to randomization
Decline participation	Patient has an opt-out bracelet or necklace		Wearing an opt out wrist band
	Family member present at the scene objects to the patient's participation		Refusal to participate
Objections to Blood Products	Known or religious objection to blood products		Wearing medical alert jewelry/bracelet, etc. found to indicate Jehovah's Witness or similar with objections to blood transfusions
IV access		Inability to obtain intravenous or interosseous access	Inability to obtain IV access to administer TP
Other		Isolated fall from standing injury mechanism	Arrival of EMS supervisor at the time ambulance transport is underway
		Isolated drowning or hanging victims	Not English or Spanish-speaking
		Isolated burns > estimated 20% total body surface area	Communication barrier at the time of eliciting refusal (non-English or non-Spanish speaking)
		Referral hospital In-patient admission	Documented "Do not resuscitate" (DNR) order found/known

Table 4. Harmonized Inclusion Criteria

Acutely injured patients with blunt or penetrating trauma in severe hemorrhagic shock

Transported by ground or air ambulance
Presence of electrical activity and/or measureable or palpable blood pressure at time of randomization
Age>/=18 years
Shock definition: Acutely injured, with presumed hemorrhagic shock from acute blood loss defined as SBP</=70 mmHg or with SBP 71-90 mmHg and HR>/=108 beats per minute
Either sex
Volume or units of crystalloid administered prior to randomization can be documented

Table 5. Harmonized Exclusion Criteria

Age <18 years
Inability to obtain intravenous or interosseous access
Penetrating cranial injury.
Traumatic brain injury with brain matter exposed.
Visibly or verbally reported pregnant woman
Cardiac arrest or CPR prior to randomization
Known prisoner
Unsalvageable injuries
Known religious objection to blood products
Patient has an opt-out bracelet, necklace or wallet card
Patient (if lucid) or family member at scene declines participation in the study

3. Timing of blood samples and identification of key parameters and assays

Timing of collection of data for key parameters that support the unifying hypotheses will be standardized across studies to the following times: 1) Emergency Department arrival (within 1 hour of arrival and prior to in-hospital transfusion of fluid administration; 2) 24 hours after ED arrival; and 3) 28-30 days after ED arrival. This represents the minimum that will be performed. Data will also be collected at other time points as described in each individual site protocol. Additional assays are included as specified in each site specific protocol (Appendices 1-3).

The clinical data both at presentation and throughout hospitalization will be obtained and recorded in individual databases established at each study site (Table 6). Clinical data entered will include a summary of injuries on admission, illness during the index admission, medical history, medications, and infectious and non-infectious complications, as well

as time and cause of death. Patient data entry will end with the index hospital stay. Outpatient information will not be included. A combined data base will be established for the combined study that will minimize need for manual data entry. This will be established and validated prior to the first interim data analysis. The ClinPortal web-based data collection tool at Washington University in Saint Louis will be used to compile and integrate data from each clinical study site.

Table 6. Key data collection will include the following

Parameter	Time		
	ED Arrival	24 Hr	30 d
Mortality (Documented by telephone contact if discharged before 30 days)	x	x	x
24 Hour Blood Transfusion Requirements (total and by blood component)		x	
Standard coagulation assays: prothrombin time (PT), international normalization ratio (INR), and fibrinogen concentration (Clauss Method)	x	x	
Thromboelastography (TEG): Tissue factor activated rapid TEG (r-TEG) will be used. Parameters will include activated clotting time (ACT, seconds), angle (alpha, degrees), coagulation time (K, seconds), maximum amplitude (MA, mm), clot strength (G, dynes/cm2), and estimated percent lysis (EPL, %).	x	x	
D-dimer	x	x	
Multiple Organ Failure (Using standard MOF checklist/criteria (TBD))		x	x
Nosocomial Infection (number of events, organism and antibiotic sensitivity)		x	x
Acute Lung Injury (Using standard ALI checklist/criteria (Appendix 5 TBD)		x	x
Transfusion Related Acute Lung Injury (Using standard TRALI checklist/criteria (Appendix 6 TBD)		x	x
Resuscitation Fluid Requirements	x	x	x
Lactate	x	x	
Arterial Blood Gases	x	x	
Platelet Count	x	x	
Hematocrit	x	x	
Red Blood Cell Count	x	x	
Hemodynamic Parameters (SBP and HR)	x		

Transport Time	x		
Time from 911 call (estimate of time of injury) to ED arrival	x		
Injury Severity Score (Calculated within 14 days of entry into study)			

Timing of data and sample collection for each study and for the harmonized approach is depicted in Figure 1.

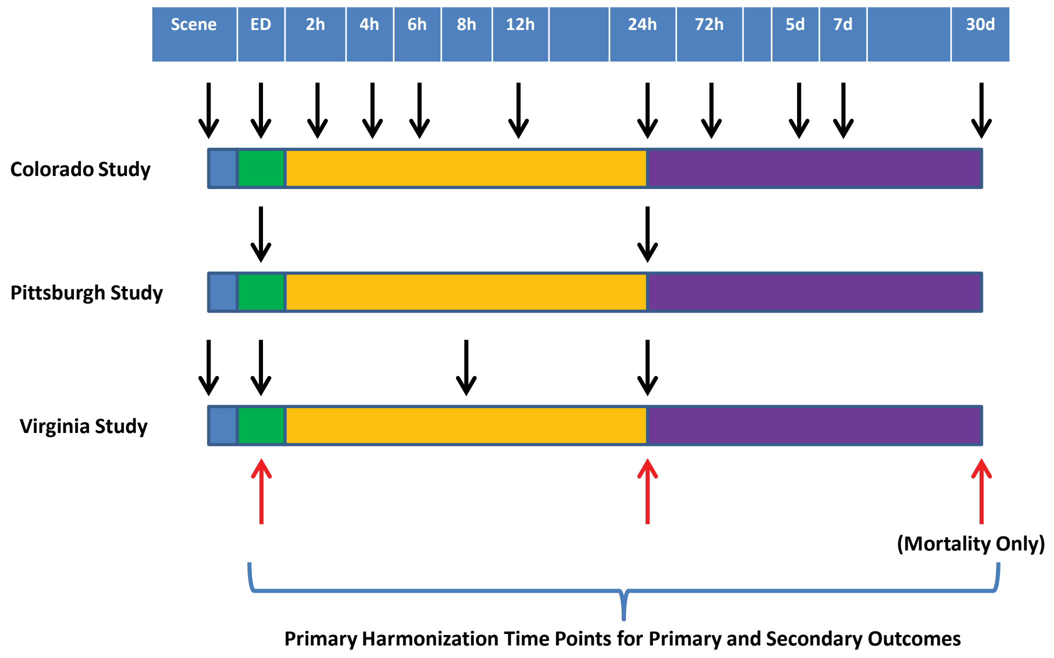

4. Adverse Events

Adverse events will be reported according to the reporting procedures established for each individual study, as described within each protocol (Appendices 1-3)

5. Methods to account for patient transport time

Time of injury will be estimated based on the time of the initial 911 call. Transport time will be calculated as the time from EMS arrival on scene to time of arrival at the ED

Figure 1. Timing of blood samples (black arrows) and data collection times for each study and for the combined harmonized study. Harmonization time points (red arrows) indicate the time points that will be harmonized across the three studies. These are the time points that support the unifying hypotheses and primary and secondary outcomes identified in the harmonization protocol.

6. Enabling language and permissions for secondary harmonization.

Each site protocol will include language that will provide permission to: archive samples, share samples with other laboratories, perform additional assays on samples, store data, transfer and share data, and perform future data analyses beyond the scope of the specific approved study and specifically delineated procedures. In addition, a maximum blood volume approval will be sought for each individual site protocol to facilitate planning for any future additional blood samples that may be included. For additional blood samples, or for any other change related to secondary harmonization or other reason, appropriate approvals will be sought. It is anticipated that each of the three studies will be included as the DoD component of the NHLBI/DoD Trans-Agency Research Consortium for Trauma-Induced Coagulopathy (TACTIC) grant program. This program will likely require the collection of samples for analysis of genetically-related parameters. Therefore, permissions will be specifically sought for collection of these types of samples. These may include additional consent procedures.

Secondary Harmonization will include the following aspects of each clinical study:

1. Selected assay procedures and reagents

2. Blood sampling and handling procedures

3. Timing of interim reviews

4. Sample processing and storage procedures

5. Timing and number of blood samples (additional harmonization beyond that stated for primary harmonization)

6. Consolidation of procedures and laboratories to run selected assays

Details for secondary harmonization will be determined later.

7. Coordinating Procedures

Communication plan. Coordination of the three separate studies will be facilitated by monthly conference calls, periodic site visits by USAMRMC personnel, and twice yearly in-person meetings. In addition, procedures will be established to report progress and for transfer data. Additional communications will be established as needed to address specific topics. Study sites will share full protocols, manuals of operations, and specific details of assays and other procedures as needed to facilitate coordination of studies. In cases where it is determined that assays or other procedures will be standardized among the three study sites, detailed procedures will be exchanged, technicians will be cross-trained, and assays (or other procedures) will be validated at each individual site.

Data consolidation plan. A specific, detailed data consolidation plan will be developed well in advance of the first interim data analysis that is planned for each study site. The time of the first interim analysis will also be the time of the first full test of the consolidated data set and all associated data transfer procedures.

Study monitoring will be conducted in accordance with USAMRMC standard procedures for monitoring human use protocols. This will include periodic site visits by study monitors, periodic progress reports, and other communications.

Data Safety Monitoring Board (DSMB) Procedures. Each individual study site will be responsible for its DSMB. Reporting will be in accordance with FDA and USAMRMC requirements.

Local protocol approvals will be the responsibility of each individual study site. All study sites will share lessons-learned with the overall team.

Investigational New Drug (IND) applications will be the responsibility of each individual study site. All study sites will share lessons-learned with the overall team.

Community consultation procedures will be the responsibility of each individual study site. All study sites will share lessons-learned with the overall team.

Secretary of the Army Approval. Each individual site protocol will require approval from the Office of the Secretary of the Army. The approvals will be facilitated by the USAMRMC Human Research Protections Office. Each individual site protocol will be submitted along with the Combined Study Harmonization Protocol to demonstrate that the studies are part of a coordinated program and to facilitate approval.

8. Data Meta-Analysis Plan

Unifying Hypotheses

The following hypotheses will be addressed by the combined study harmonization plan and meta-analysis.

It is expected that all patients enrolled in the COMBAT and PUPTH studies will be included in the meta-analyses that address the primary and secondary unifying hypotheses. The PAMPer Study includes five enrolling sites. Two of these sites will have a slightly different prehospital treatment. Procedures at these two sites include the possibility of initiating transfusion of packed red blood cells enroute, prior to ED arrival. It is anticipated that for some parameters, this will require sub-analysis. For the purpose of addressing the primary and secondary outcomes, these sites will be excluded. Overall sample size projections, with and without PAMPer sites allowing prehospital packed red blood cells, are shown in Table 7.

Primary Outcome

1. Prehospital administration of 2 units of plasma will reduce mortality at 30 days after ED arrival

Secondary Outcomes

2. Prehospital administration of 2 units of plasma will reduce mortality at time of emergency department (ED) arrival

3. Prehospital administration of 2 units of plasma will reduce mortality at 24 hours after ED arrival

4. Prehospital administration of 2 units of plasma will reduce 24 hour transfusion requirements

5. Prehospital administration of 2 units of plasma will improve standard coagulation parameters at the time of ED arrival

6. Prehospital administration of 2 units of plasma will improve clot viscoelastic properties (thromboelastograph (TEG) parameters) at the time of ED arrival

7. Prehospital administration of 2 units of plasma will improve hemodynamic parameters (systolic blood pressure (SBP) and heart rate (HR)) at the time of ED arrival

8. Prehospital administration of 2 units of plasma will improve cellular hematologic parameters (hematocrit, red cells, platelet count) at the time of ED arrival

9. Prehospital administration of 2 units of plasma will improve metabolic status (lactate, blood gases, pH, base deficit) at the time of ED arrival

10. Prehospital administration of 2 units of plasma will improve International Society on Thrombosis and Haemostasis Disseminated Intravascular Coagulation Score (ISTH DIC Score) at the time of ED arrival and at 24 hours after ED arrival

Table 7. Sample Size Projections

Site	Total Sample Size	Sample Size Excluding Sites That Transfuse Red Cells Enroute
Colorado	150	150
Pittsburgh	545	375
Virginia	270	270
Total	965	795

Meta-analysis objectives:

Table 8 shows the various planned primary (P), secondary (S), and exploratory (E) outcome measures across the three studies. Analysis of ED arrival and 24 hour mortality will provide a more fine-grained look at the mortality and may provide insights into trends in other variables (time dependency, survivor bias, etc.). In addition, analyses will be performed to support each of the secondary hypotheses (hypotheses 2-10) described above.

Table 8. Pre-planned Primary and Secondary Outcome Measures

Outcome	PAMPer	COMBAT	PUPTH
30/28 Day Mortality	P	P	S6
Multiple Organ Failure	S1	S1	S6
Post Admission Coagulopathy	S3	S2	S3
Clot Strength	S3	S3	S3
Acidosis/Shock		S4	S4
24 Hour Mortality		E1	
Blood Product Use	S1, S2	E5	S5
Nocosomial Infection	S1		S6
Lung Injury	S1	E3	
TRALI	S1		
1st 24hr Vasopressor Support	S2		
Inflammation	S4		
Ventilator (Free) Days		E3	S6

Statistical Analysis Plan

For all primary and secondary endpoints the null hypothesis that Plasma=Standard of Care (SOC) will be tested against the alternative hypothesis that Plasma<SOC (Plasma>SOC for Clot Strength) using the appropriate independent sample test at a significance cutoff of 0.05. For normally distributed continuous variables, or those which can be log transformed to normality (e.g. transfusion requirement), a t-test will be used. For non-normally distributed continuous variables the Mann-Whjtney U test will be used. Binary endpoints (e.g. 30 day mortality) will be tested using the Fisher Exact Test.
For all secondary endpoints and exploratory subgroup analysis, the significance cutoff will be Bonferroni corrected. If warranted by highly correlated endpoints which are individually significant but do not meet Bonferroni corrected

significance cutoffs, exploratory Westfall-Young Bootstrap (sampled permutation) minP based p-values and step-down null hypothesis rejection decisions will also be presented.

Size and power of pooled data analysis:

Individual participant data (IPD) from the three studies will be pooled for meta-analysis using a one-step approach. Potential study specific clustering effects will be accounted for by adding study membership as a random effects covariate. This constitutes a two level grouped design which will allow covariate analysis and adjustment at the patient and study levels. Any study terminated at or after the first interim analysis, but before planned completion due to adverse events will be included in the meta-analysis.

Figures 2 provides the power estimates for a range of potential pooled sample sizes for 30 day survival. An estimated control mortality of 22% from the PAMPer study was chosen over the slightly less conservative 26% estimate from the COMBAT study. The combined study power curves represent a best case scenario which assumes a negligible effect from covariates and an interclass correlation (ICC) of 0. We expect a low ICC based on our high degree of harmonization and demonstrated equivalency of primary treatments. Adjusting for covariates at the patient and study levels will reduce their negative impact on grouped power. The unequal number of patients expected across the three studies will, with a non-0 ICC, work to slightly decrease expected power.

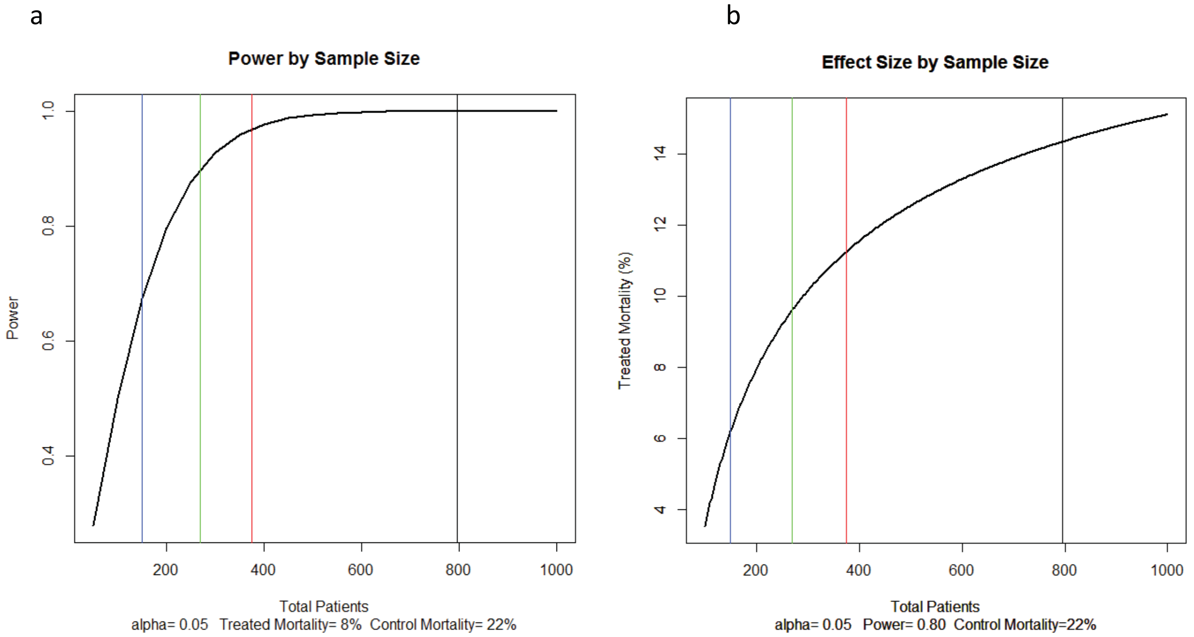

Figure 2. Power (a) and detectable effect size (b) as a function of sample size for 30 day mortality. The curved black line in each plot represents a 30 day control mortality estimate of 22% (PAMPer study). Vertical lines represent the expected number of total patients for the PAMPer (red), COMBAT (blue), and PUPTH (green) studies. Patient estimates for the PAMPer study exclude sites which allow administration of prehospital packed red blood cells and are taken before the 1.75 multiplication adjustment for grouped design. The black vertical line gives the pooled sum of these patient values. The intersection of the curved and vertical black lines represents the upper limit of meta-analysis power (a) and effect size (b). Power calculations use a two tailed test of proportions differences.

Subgroup analysis:

Table 9 shows planned subgroup analyses for the three studies. Trauma type (blunt vs. penetrating), head injury, and shock are shared across two of the three studies and will be conducted for the primary and secondary meta-analysis outcomes. Additionally, any subgroup analysis which shows treatment effects in an individual study will be repeated in the meta-analysis if permitted by collected data. Where differing metrics are planned for a single subgroup (e.g. lactate/BE vs. systolic blood pressure + heart rate for shock), the metric used for meta-analysis will be that which is present in, and most directly comparable across, the three studies.

Table 9. Pre-planned Subgroup Analysis

**Subgroup**	**PAMPer**	**COMBAT**	**PUPTH**
Blunt vs. Penetrating Trauma	yes	yes	
Brain Injury/TBI	yes	yes	
Shock/hypoperfusion		yes	yes
24hr Transfusion Req.	yes		yes
Scene vs. Hospital Referral	yes		
Number of Surgeries			yes
Vit. K Antagonist Medication	yes		
PRBC Req.	yes		yes
Antiplatelet Medication	yes		
Transport Time	yes		
Injury Severity			yes

Covariate adjustment and missing data etc:

Table 10 shows the planned covariate adjustments for the three studies. A similar approach will be taken as for subgroup analysis, using multiple regression to adjust for covariates which are planned in at least 2/3 studies or those which show significant treatment significant group imbalance in at least one study. For this analysis, a single SBP/HR shock metric will be used.

Table 10. Pre-planned Covariate Adjustment

	**Covariate**	**PAMPer**	**COMBAT**	**PUPTH**
Demographic	Age	yes	yes	
	Gender	yes	yes	
Injury	Severity Score		yes	yes
	Blunt vs. Penetrating	yes	yes	
	Brain: TBI/GCS	yes		yes
Shock	Field SBP		yes	
	Field HR		yes	
	Field Hemocrit		yes	
	Lactate+BE			yes
Coagulation	INR		yes	

Blood	Blood Transfusion Units	yes	yes
	Pre-Hospital Crystalloid	yes	
Other	Site	yes	
	Transport Times	yes	

As mentioned above, we plan to account for major study cite differences by treating study membership as a random effects covariate. In addition, we will assess study site heterogeneity of all potential covariates from table 10 including pre-hospital crystalloid volume. While not conclusive, this analysis in conjunction with planned covariate analysis may suggest predominant causes for any study cite treatment differences.

For the primary and secondary meta-analysis outcomes, missing data is expected to be very rare and will be imputed using multiple imputation. One potential issue related to missing data is that of the effect of early mortality on additive metrics such as total 24 hour blood product use. While not a longitudinal variable as such, 24 hour blood product use is likely to be correlated with time of early death. We will treat blood product use between death and 24hours as “non-ignorable missing data with known mechanism” and use a maximum likelihood modeling approach to impute the “missing” portion.

Intent to treat analysis: primary and secondary outcome data will be collected in all patients regardless of treatment received. An “intent-to-treat” approach will be used for all primary/secondary outcome analyses, i.e., we will compare the outcomes of the two groups according to the group assignment at time of randomization, regardless of what treatment participants actually received. In addition to the “intent to treat” approach, the harmonized combined data will also support exploratory analyses, which may incorporate analysis based on the treatment received including red blood cell transfusion.

9. Annexes

a. Each site protocol

c. Timeline of harmonized protocol events (TBD)

d. Combined dataset format (TBD)

e. Coordination plan with TACTIC (TBD)

Clinical Protocol Appendix 9

PAMPer Roles and Responsibilities at UPMC January 2013

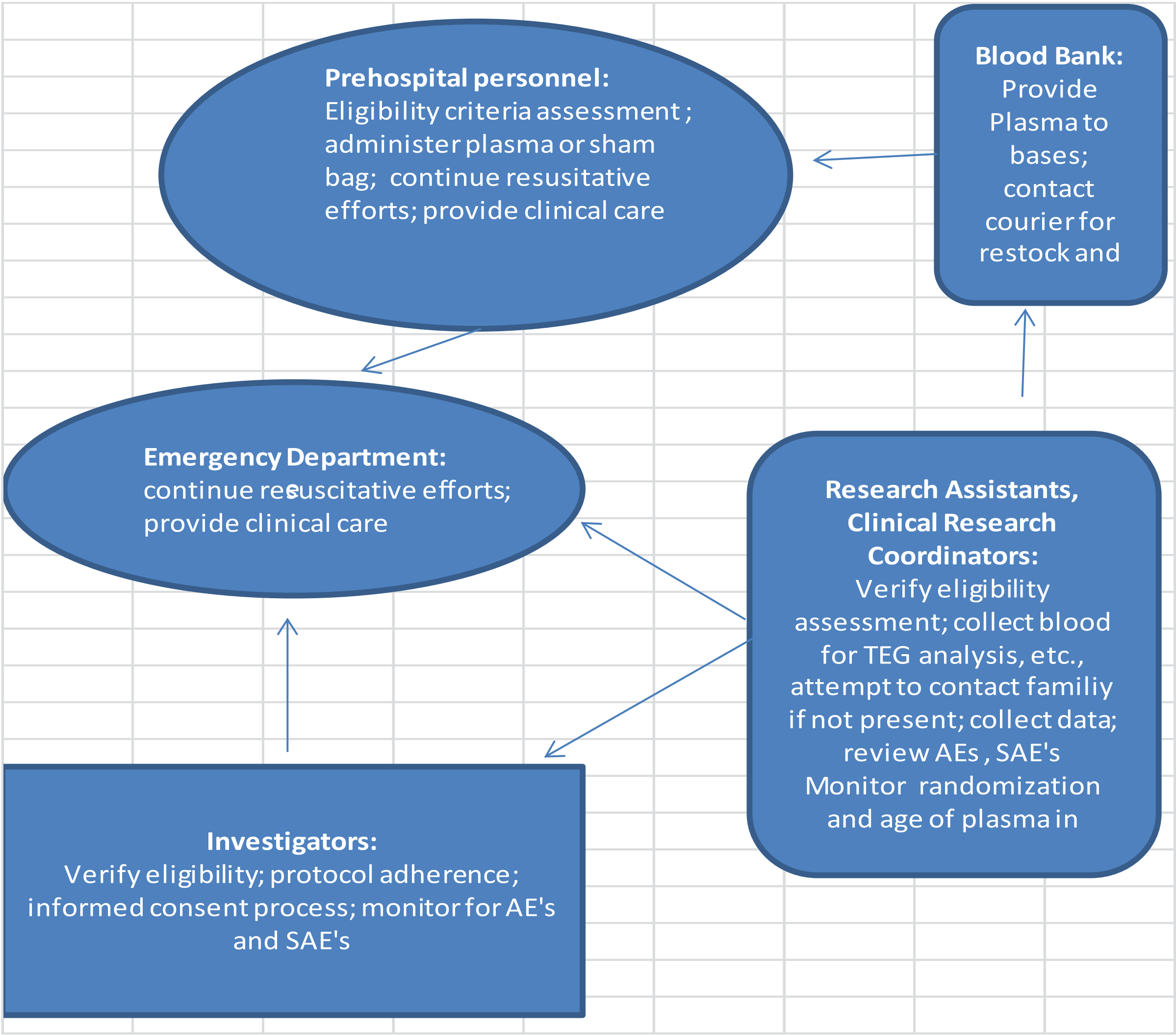

Supplement: Supplement 1. — Trial Protocol [file jamanetwopen-e2016869-s001.pdf]
