# Supplementary material for: Association of Prehospital Plasma With Survival in Patients With Traumatic Brain Injury: A Secondary Analysis of the PAMPer Cluster Randomized Clinical Trial
Source: JAMA Netw Open. 2020 Oct 15;3(10):e2016869. doi: 10.1001/jamanetworkopen.2020.16869 (PMC7563075; doi:10.1001/jamanetworkopen.2020.16869)
Supplement: Supplement 2. — eAppendix. Supplemental Methods eTable 1. Traumatic Brain Injury (TBI) Subtypes eTable 2. Causes of Death for Patients With CT-Positive Traumatic Brain Injury (TBI) eTable 3. Generalized Estimating Equations (GEE) Model Coefficients Without Adjustment for Confounders eTable 4. Generalized Estimating Equations (GEE) Model Coefficients eTable 5. Cox Proportional Hazard Model for 30-Day Mortality Among Patients With TBI eFigure 1. TBI Biomarker Concentration in Patients With and Without TBI at 0, 24, and 72 Hours Post Admission eFigure 2. TBI Biomarker Concentration Compared Across the Trial Arms at 0, 24, and 72 Hours Post Admission eFigure 3. Unadjusted Kaplan Meier 30-Day Survival Analysis Comparing Plasma and Standard Care Arms Among Patients With TBI and Grouped by Transport Origin eReferences [file jamanetwopen-e2016869-s002.pdf]

Supplementary Online Content

Gruen DS, Guyette FX, Brown JB, et al. Association of prehospital plasma with survival in patients with traumatic brain injury: a secondary analysis of the PAMPer cluster randomized clinical trial. *JAMA Netw Open*. 2020;3(9):e2016869.
doi:10.1001/jamanetworkopen.2020.16869

eAppendix. Supplemental Methods
eTable 1. Traumatic Brain Injury (TBI) Subtypes
eTable 2. Causes of Death for Patients With CT-Positive Traumatic Brain Injury (TBI)
eTable 3. Generalized Estimating Equations (GEE) Model Coefficients Without Adjustment for Confounders
eTable 4. Generalized Estimating Equations (GEE) Model Coefficients
eTable 5. Cox Proportional Hazard Model for 30-Day Mortality Among Patients With TBI
eFigure 1. TBI Biomarker Concentration in Patients With and Without TBI at 0, 24, and 72 Hours Post Admission
eFigure 2. TBI Biomarker Concentration Compared Across the Trial Arms at 0, 24, and 72 Hours Post Admission
eFigure 3. Unadjusted Kaplan Meier 30-Day Survival Analysis Comparing Plasma and Standard Care Arms Among Patients With TBI and Grouped by Transport Origin
eReferences

This supplementary material has been provided by the authors to give readers additional information about their work.

© 2020 Gruen DS et al. *JAMA Network Open*.

eAppendix. Supplemental Methods

The Prehospital Air Medical Plasma (PAMPer) trial randomized patients by air ambulance base for one- month time intervals to receive either standard care fluid resuscitation (crystalloid or crystalloid and packed red blood cells) or two units of thawed plasma, either group AB or group A with a low anti-B antibody titer (<1:100), followed by the standard care fluid resuscitation.

From May 2014 through October 2017, 7275 patients transport by 27 individual air medical bases to 9 participating trauma centers were assessed for eligibility. The PAMPer trial included 501 trauma patients that met all the inclusion criteria and none of the exclusion criteria and with approximately half of the patients randomized to each arm. We included blunt or penetrating injured patients transported from the scene of injury or a referral hospital to a PAMPer site if they had at least one episode of hypotension (systolic blood pressure<90 mm Hg) and tachycardia (heart rate>108 beats per minute) or if they had any severe hypotension (systolic blood pressure<70 mm Hg), either before the arrival of air medical transport or any time before arrival at the trauma center. We excluded patients older than 90 years of age or less than 18 years of age, those without intravenous or intraosseous access, those with an isolated fall from standing, those with documented cervical cord injury, those who were prisoners, those who were pregnant, those with traumatic cardiac arrest for greater than five minutes, those with penetrating brain injury, those with isolated drowning or hanging, those with burns with total body surface area greater than 20%, those admitted as an inpatient at an outside referral hospital, if they or a family member voiced an objection to participation in the trial at the scene of the injury, or if they were wearing an “opt-out” bracelet. The primary outcome in the PAMPer trial was 30-day mortality. Twenty patients (10 in each arm) were discharged prior to 30 days and did not have an outcome available. For these patients, discharge status was used as a proxy for 30-day survival. The cause of death for each patient was adjudicated by a site investigator using a predefined list of options. The determination of a patient’s race was based upon admission data obtained from site research nurses or coordinators and available in the electronic health record. This information was included to describe baseline characteristics of the primary analysis cohort. The PAMPer trial was not blinded, but the treatment assignments were concealed to personnel who assessed the trial outcomes.

The PAMPer trial outlined nine predefined subgroups for secondary analysis including traumatic brain injury (TBI). These subgroups included: 1a.) patients who did or did not require massive transfusion in the first 24 hours, 1b.) patients who required≥4 units of PRBCs, 2.) patients who received or did not receive prehospital PRBC transfusion, 3.) patients with traumatic brain injury, 4.) patients enrolled from the scene of injury versus those enrolled from a referral hospital, 5.) patients with a preinjury history of vitamin K antagonist medication versus those without, 6.) patients with preinjury history of antiplatelet medication, 7.) patients who suffered blunt injury as compared to those who suffered penetrating injury, and 8.) patients with high versus low field to ED transport times. The full study protocol and analysis plan is publicly available (https://clinicaltrials.gov/ct2/show/NCT01818427).

In this analysis, TBI was defined as any finding consistent with TBI as defined by a radiologist upon initial head CT, including extra-axial lesions, epidural hematoma, subdural hematoma, subarachnoid and intraventricular hemorrhage, CSF leak, intra-axial injury, intracerebral hematoma, traumatic axonal injury, and brain stem injury. We excluded skull fractures without evidence of brain injury.

We analyzed markers GFAP and UCH-L1 in order to assess possible differences in TBI across arms of the PAMPer trial. We collected blood samples from 108 TBI patients and 4 control (no

© 2020 Gruen DS et al. *JAMA Network Open.*

TBI) patients at emergency department (ED) admission (0 h), and 24 h and 72 h post admission. Sampling was not feasible in some patients due to time-sensitive procedures or early death. Sample analysis for GFAP and UCH-L1 was performed by a single laboratory (Abbott, Abbott Park, IL, USA) in blinded fashion using prototype chemiluminescent microparticle immunoassays on the ARCHITECT (Abbott, Abbott Park, IL, USA) platform. The GFAP assay is a two-step immunoassay using monoclonal antibodies for capture and detection of GFAP and GFAP breakdown products. The assay has a limit of detection (LoD) of 2 pg/mL and analytical measuring interval (AMI) from 5 to 50,000 pg/mL, based on assay linearity and imprecision.[1] The UCH-L1 assay is a two-step immunoassay using monoclonal antibodies for capture and detection. The assay has a limit of detection of 10 pg/mL and analytical measuring interval (AMI) from 30 to 25,000 pg/mL, based on assay linearity and imprecision.46 Samples greater than the AMI were diluted 1:10 (GFAP) or 1:4 (UCH-L1) and retested to obtain quantitative results.

We first evaluated 30-day mortality across groups with and without TBI (binned as a dichotomous vari- able) using a generalized estimating equations (GEE) model in the geepack v1.2-1[2] package. The plasma and TBI interaction was assessed for statistical significance, accounting for trial cluster effects and multiple confounders. Confounding variables age, head abbreviated injury scale (AIS), sex, earliest (prehospital) Glasgow Coma Scale (GCS), injury severity score (ISS), prehospital fluid resuscitation requirements, pre- hospital shock, and transport time were included based on differences between the groups and any known clinical confounders as previously reported.[3] We also tested the interaction between prehospital plasma and ISS. The prehospital plasma and ISS interaction was not significant and it was not included in our final model.

We performed an analysis of survival with the use of a Cox proportional hazard with shared frailty model to evaluate the treatment effect with adjustment for possible confounding factors and site clustering on survival.[4] We generated the model for the primary outcome and assessed prehospital differences across the groups when randomization for prehospital plasma occurred. Covariates were selected based on differences between the groups and any known clinical confounders as previously reported.[3] The model was generated for the primary outcome in patients with TBI. We included the covariates age, non-head AIS, head AIS, sex, earliest available (prehospital) GCS, prehospital crystalloid, PRBC, and plasma resuscitation (study intervention), prehospital shock (SBP<70), prehospital intubation, transport time, and transfer status. Age, head AIS, sex, GCS, crystalloid, and PRBC were assessed as continuous variables. Non-head AIS (abdomen, chest, and extremity categories <3 for all categories or abdomen, chest, and extremity categories ≥3 for one or more categories), plasma, intubation, transport time, and transfer status were treated as categorical variables.

We explored the association between prehospital plasma and both TBI severity and concomitant polytrauma by comparison of hazard ratios from a fitted Cox proportional hazard model. We defined TBI with mild to moderate traumatic injury in other body regions as the presence of brain injury on CT and AIS < 3 in abdomen, chest, and extremity AIS categories. We defined TBI with severe polytrauma by the presence of brain injury on CT scan and AIS ≥ 3 in abdomen, chest, or extremity categories. Finally, we assessed the interaction between prehospital plasma and earliest measured prehospital GCS. We calculated P values for the interaction between plasma and standard care groups with 30-day mortality as the outcome.

We analyzed survival for TBI patients transported from the scene and those patients transferred from an outside hospital as proxies for early versus late prehospital plasma fluid administration. We performed Kaplan-Meier survival analysis to compare transport origin across study arms.

© 2020 Gruen DS et al. *JAMA Network Open*.

eTable 1: Traumatic Brain Injury (TBI) Subtypes. Multiple subtypes were documented for some patients.

TBI Subtype	*n* = 166 (%)
Subdural Hematoma/Hemorrhage	86 (51.8)
Epidural Hematoma/Hemorrhage	11 (6.6)
Contusion	31 (18.7)
Other	110 (66.3)

© 2020 Gruen DS et al. *JAMA Network Open*.

eTable 2: Causes of Death for Patients With CT-Positive Traumatic Brain Injury (TBI).

Cause of Death	*n* = 77 (%)
Hemorrhage/Hypovolemic Shock	9 (11.7)
Traumatic Brain Injury/Herniation	42 (54.5)
Withdrawal of Life Sustaining Therapy	17 (22.1)
Sepsis	1 (1.3)
Acute Respiratory Distress Syndrome	1 (1.3)
Multisystem Organ Failure	2 (2.6)
Cardiac	2 (2.6)
Other	3 (3.9)

© 2020 Gruen DS et al. *JAMA Network Open*.

eTable 3: Generalized Estimating Equations (GEE) Model Coefficients Without Adjustment for Confounders. TBI=traumatic brain injury. PH Plasma refers to the study intervention. TBI*PH Plasma refers to the interaction term (between TBI and the prehospital plasma intervention).

Coefficient	*Estimate*	*P Value*
PH Plasma	-0.26	0.05
TBI	1.53	<0.001
TBI*PH Plasma	-0.63	0.03

© 2020 Gruen DS et al. *JAMA Network Open*.

eTable 4: Generalized Estimating Equations (GEE) Model Coefficients. PH=prehospital. AIS=abbreviated injury scale. GCS=Glasgow Coma Scale. ISS=Injury Severity Score. MOI=mechanism of injury. PRBC=packed red blood cells. SBP=systolic blood pressure. TBI=traumatic brain injury. Age, AIS head, ISS, PH crystalloid, and PH PRBC treated as continuous variables. MOI, PH blood, PH GCS<8, PH plasma, PH SBP<70, PH time, Sex, and TBI were treated as categorical variables. PH Plasma refers to the study intervention. TBI*PH Plasma refers to the interaction term (between TBI and the prehospital plasma intervention).

Coefficient	*Estimate*	*P Value*
Age	0.019	0.005
AIS Head	-0.0087	0.90
ISS	0.022	0.004
MOI	-0.78	0.17
PH Blood	0.68	0.14
PH Crystalloid	-0.00019	0.004
PH GCS<8	2.1	<0.001
PH Plasma	-0.31	<0.001
PH PRBC Volume	0.0030	0.99
PH SBP<70	0.40	0.007
PH Time	-0.31	0.11
Sex	0.12	0.56
TBI	0.47	0.14
TBI*PH Plasma	-0.42	0.04

© 2020 Gruen DS et al. *JAMA Network Open*.

eTable 5: Cox Proportional Hazard Model for 30-Day Mortality Among Patients With TBI. CI=confidence interval. PH=prehospital. AIS=abbreviated injury scale. GCS=Glasgow Coma Scale. ISS=Injury Severity Score. PRBC=packed red blood cells. PH Plasma refers to the study intervention. Age, AIS head, GCS, crystalloid, and PRBC are treated as continuous variables. Non-head AIS (≥3), intubation, plasma, sex, transport time, and transfer status are treated as categorical variables.

Covariate	*Hazard Ratio*	95% *CI*	*P Value*
Age	1.01	1.00-1.02	0.19
Head AIS	0.89	0.73-1.09	0.27
Non-Head AIS	0.71	0.42-1.21	0.21
PH Crystalloid	1.00	1.00-1.00	0.01
PH GCS	0.77	0.67-0.87	<0.001
PH Intubation	1.20	0.48-2.98	0.70
PH Plasma	0.55	0.33-0.94	0.03
PH PRBC	1.19	1.02-1.40	0.03
PH SBP<70	1.69	1.01-2.84	0.05
Sex	1.32	0.74-2.35	0.35
Transfer Status	0.72	0.33-1.59	0.42
Transport Time	0.96	0.58-1.59	0.89

© 2020 Gruen DS et al. *JAMA Network Open.*

Figures

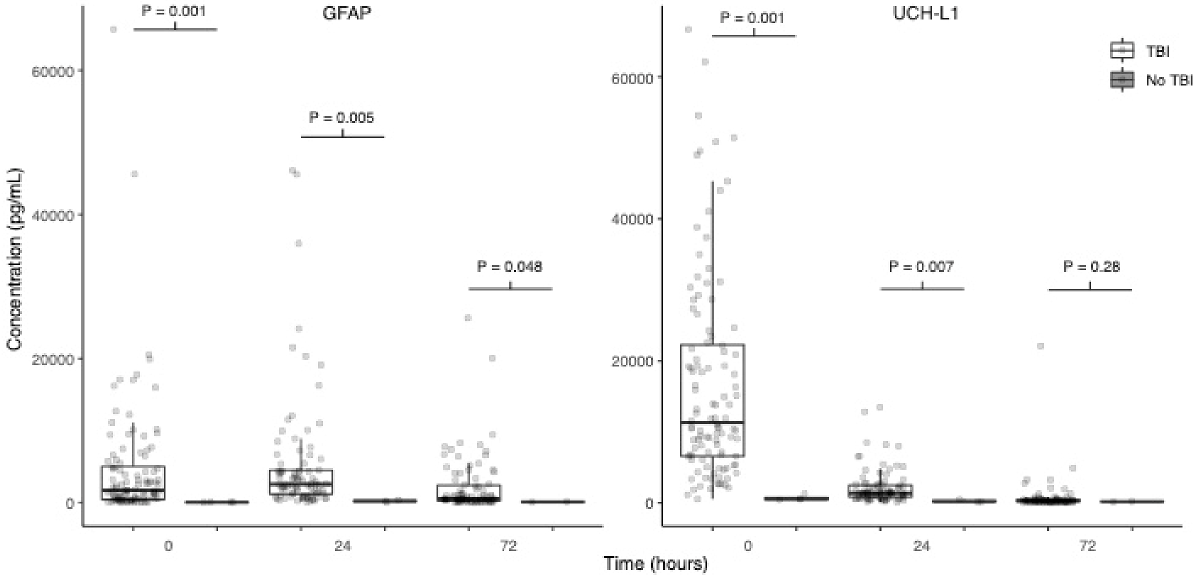

eFigure 1: TBI Biomarker Concentration in Patients With and Without TBI at 0, 24, and 72 Hours Post Admission. GFAP=glial fibrillary acidic protein. UCH-L1=Human ubiquitin C-terminal hydrolase. Lines within the bars represent medians. The lower and upper hinges correspond to the first and third quartiles (the 25th and 75th percentiles). P values are calculated by the Mann-Whitney-U test. P-values are not adjusted for multiple comparisons.

© 2020 Gruen DS et al. *JAMA Network Open.*

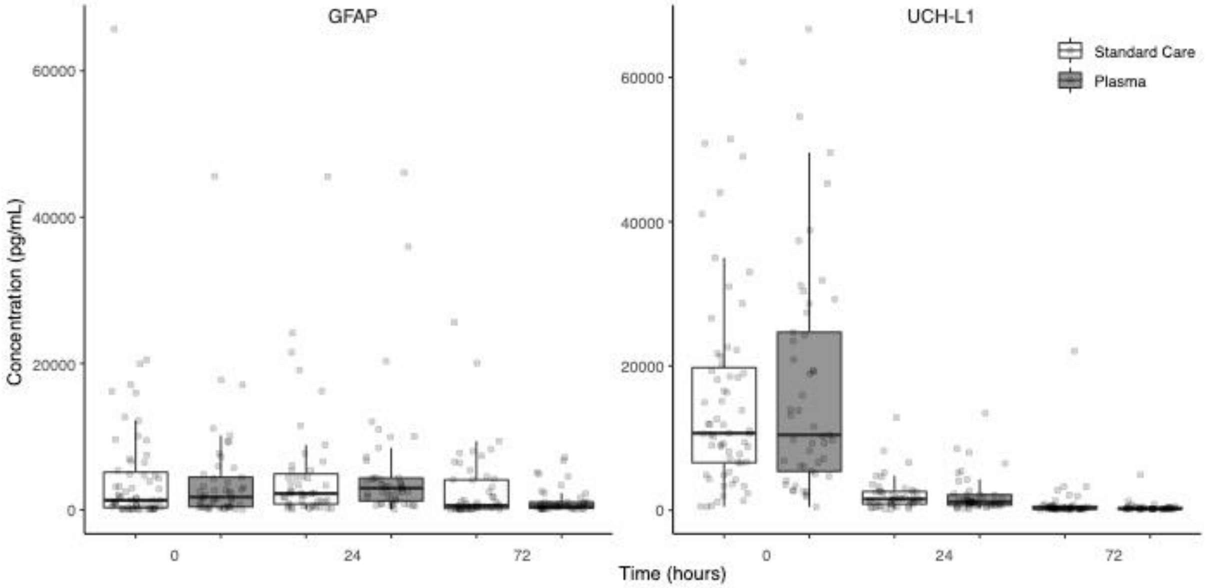

eFigure 2: TBI Biomarker Concentration Compared Across the Trial Arms at 0, 24, and 72 Hours Post Admission. GFAP=glial fibrillary acidic protein. UCH-L1=Human ubiquitin C-terminal hydrolase. Lines within the bars represent medians. The lower and upper hinges correspond to the first and third quartiles (the 25th and 75th percentiles). P>0.05 for all time points as calculated by the Mann-Whitney-U test. P-values are not adjusted for multiple comparisons.

© 2020 Gruen DS et al. *JAMA Network Open*.

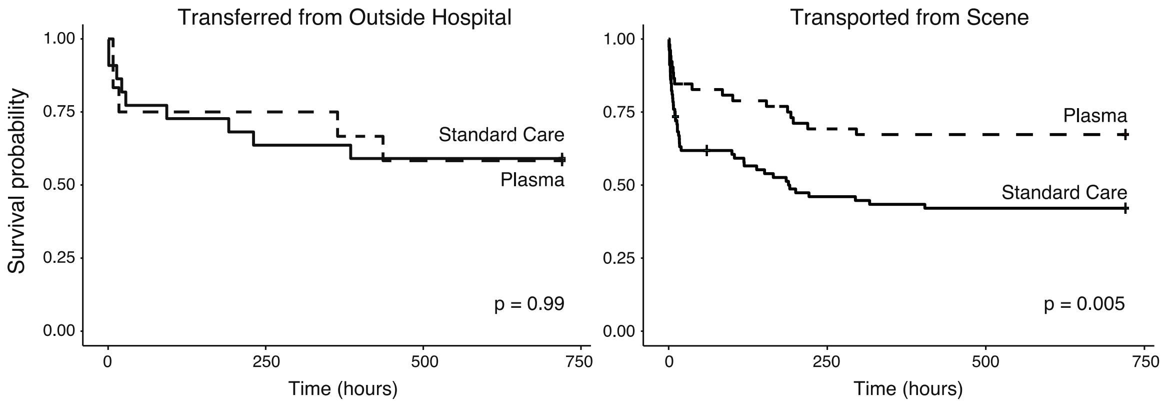

	Transferred from Outside Hospital: 0	250	500	750	Transported from Scene: 0	250	500	750
**Number at risk**								
Plasma	22	14	13	0	52	36	35	0
Standard Care	12	9	7	0	79	35	32	0
**Censored**								
Plasma	0	0	0	13	0	0	0	35
Standard Care	0	0	0	7	0	2	2	34

eFigure 3: Unadjusted Kaplan Meier 30-Day Survival Analysis Comparing Plasma and Standard Care Arms Among Patients With TBI and Grouped by Transport Origin. P values calculated with the Log-rank test.

© 2020 Gruen DS et al. *JAMA Network Open*.

eReferences

[1] Establishing and Verifying an Extended Measuring Interval Through Specimen Dilution and Spiking. 1st ed. EP34. Wayne, PA: Clinical and Laboratory Standards Institute; 2018.

[2] Halekoh U, Højsgaard S, Yan J. The R package geepack for generalized estimating equations. Journal of Statistical Software. 2006;15(2):1–11.

[3] Sperry JL, Guyette FX, Brown JB, Yazer MH, Triulzi DJ, Early-Young BJ, et al. Pre- hospital Plasma during Air Medical Transport in Trauma Patients at Risk for Hemorrhagic Shock. New England Journal of Medicine. 2018 7;379(4):315–326.

[4] Austin PC. A tutorial on multilevel survival analysis: Methods, models and applications. International Statistical Review. 2017;85(2):185–203.

© 2020 Gruen DS et al. *JAMA Network Open.*
