# Supplementary material for: Association of Prehospital Plasma With Survival in Patients With Traumatic Brain Injury: A Secondary Analysis of the PAMPer Cluster Randomized Clinical Trial
Source: JAMA Netw Open. 2020 Oct 15;3(10):e2016869. doi: 10.1001/jamanetworkopen.2020.16869 (PMC7563075; doi:10.1001/jamanetworkopen.2020.16869)
Supplement: Supplement 3. — Data Sharing Protocol [file jamanetwopen-e2016869-s003.pdf]

Data Sharing Statement

Gruen. Association of Prehospital Plasma With Survival in Patients With Traumatic Brain Injury. *JAMA Netw Open*. Published October 15, 2020. 10.1001/jamanetworkopen.2020.16869

Data
Data available: No

Additional Information
Explanation for why data not available: The full study protocol, statistical analysis plan, and supplementary material for the primary trial is publicly available (https://clinicaltrials.gov/ct2/show/NCT01818427). The analysis code for this study is available on GitHub (https://github.com/dgru/pamper-tbi). Individual participant data is not available.
